# A protein palmitoylation cascade regulates microtubule cytoskeleton integrity in *Plasmodium*

**DOI:** 10.15252/embj.2019104168

**Published:** 2020-05-12

**Authors:** Xu Wang, Pengge Qian, Huiting Cui, Luming Yao, Jing Yuan

**Affiliations:** ^1^ State Key Laboratory of Cellular Stress Biology Innovation Center for Cell Signal Network School of Life Sciences Xiamen University Xiamen China

**Keywords:** cytoskeleton, malaria parasite, microtubule, ookinete, palmitoylation, Cell Adhesion, Polarity & Cytoskeleton, Microbiology, Virology & Host Pathogen Interaction, Post-translational Modifications, Proteolysis & Proteomics

## Abstract

Morphogenesis of many protozoans depends on a polarized establishment of cytoskeletal structures. In malaria‐causing parasites, this can be observed when a round zygote develops into an elongated motile ookinete within the mosquito stomach. This morphogenesis is mediated by the pellicle cytoskeletal structures, including the inner membrane complex (IMC) and the underlying subpellicular microtubules (SPMs). How the parasite maintains the IMC‐SPM connection and establishes a dome‐like structure of SPM to support cell elongation is unclear. Here, we show that palmitoylation of N‐terminal cysteines of two IMC proteins (ISP1/ISP3) regulates the IMC localization of ISP1/ISP3 and zygote‐to‐ookinete differentiation. Palmitoylation of ISP1/ISP3 is catalyzed by an IMC‐residing palmitoyl‐S‐acyl‐transferase (PAT) DHHC2. Surprisingly, DHHC2 undergoes self‐palmitoylation at C‐terminal cysteines via its PAT activity, which controls DHHC2 localization in IMC after zygote formation. IMC‐anchored ISP1 and ISP3 interact with microtubule component β‐tubulin, serving as tethers to maintain the proper structure of SPM during zygote elongation. This study identifies the first PAT–substrate pair in malaria parasites and uncovers a protein palmitoylation cascade regulating microtubule cytoskeleton.

## Graphical abstract

Polarised localisation of inner membrane complex proteins ISP1/ISP3 controls ookinete morphogenesis in *Plasmodium* via regulation of microtubule network organization.

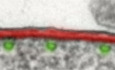

## Introduction

Malaria remains a global life‐threatening infectious disease, causing approximately half a million deaths annually (WHO, 2018). Malaria parasites have a life cycle switching between a vertebrate and a female *Anopheles* mosquito. Differentiation of gametocytes to gametes in the mosquito midgut is initiated immediately after a blood meal. Fertilization of male and female gametes results in a diploid zygote. Within 12–24 h, the spherical zygotes undergo morphological changes in “protrusion–elongation–maturation” to differentiate into crescent‐shaped mature ookinetes (Guttery *et al*, 2015; Bennink *et al*, 2016). Only polarized and elongated ookinetes possessing gliding motility are capable of traversing the mosquito midgut epithelium to colonize at basal lumen where thousands of sporozoites develop within an oocyst (Aly *et al*, 2009; Josling & Llinas, 2015).

The ookinete, as well as two other invasive “zoite” stages (merozoite and sporozoite), possesses an apicomplexan‐specific cortical structure named the pellicle (Kono *et al*, 2013). From outside to inside, the pellicle has three layers of discernible structures: parasite plasma membrane (PPM), a double‐membrane vacuolar organelle inner membrane complex (IMC), and subpellicular microtubules (SPMs) functioning as cytoskeleton (Kono *et al*, 2013). The mature invasive zoites of apicomplexan parasites also possess a specialized apical complex, which is minimally composed of secretory organelles and apical polar ring (Russell & Burns, 1984; Frenal *et al*, 2017). The apical polar ring functions as the microtubule‐organizing center (MTOC) for nucleating the assembly of SPM underling the IMC (Morrissette & Sibley, 2002). The crescent shapes of ookinete and sporozoite are presumably determined by special arrangement of SPM emanating from the apical polar ring (Khater *et al*, 2004). The IMC is composed of flattened and interconnected cisternae and is associated with SPM presumably via the intermediate filament‐like protein network (Morrissette *et al*, 1997). IMC and SPM function coordinately to maintain zoite polarity and morphology (Poulin *et al*, 2013), which are critical for host cell traversal/invasion (Khater *et al*, 2004; Volkmann *et al*, 2012; Absalon *et al*, 2016; Parkyn Schneider *et al*, 2017) and cytokinesis of progeny formation (Absalon *et al*, 2016). During *Plasmodium* life cycle, the assembly and disassembly of IMC and SPM are highly stage‐specific. Ookinete differentiation from a fertilized zygote includes assembly of IMC, apical polar ring, and SPM structures that occur initially at an apical polarity patch of zygote protrusion and extends along the periphery for zygote elongation (Kono *et al*, 2013). While zygote‐to‐ookinete differentiation has been defined by light and electron microscopy (Canning & Sinden, 1973; Raibaud *et al*, 2001), the molecular mechanisms underlying this morphogenesis remain largely unknown.

The IMC‐residing protein ISP (IMC subcompartment protein) is restricted to the phylum apicomplexa and plays key roles in parasite biology. Two ISP members, ISP1 and ISP3, are found in *Plasmodium* parasites (Poulin *et al*, 2013), and display clear apical polarity of expression in zygotes (Poulin *et al*, 2013; Gao *et al*, 2018), suggesting possible role in zygote protrusion or elongation. However, parasites with disruption of ISP1 display a modest decrease in zygote‐to‐ookinete differentiation (Gao *et al*, 2018), while ISP3 depletion has no significant effect on this process (Poulin *et al*, 2013). So far, the precise function and expression regulation of ISP1 and ISP3 in the zygote‐to‐ookinete differentiation remain unknown.

Palmitoylation, a post‐translational modification (PTM) covalently adding C‐16 fatty acyl moieties at specific cysteines, plays a crucial role in the biology of many organisms, including *Plasmodium* parasites (Cabrera *et al*, 2012; Jones *et al*, 2012; Santos *et al*, 2016; Tay *et al*, 2016; Brown *et al*, 2017), by regulating protein localization and protein–protein interactions (Fukata & Fukata, 2010; Chen *et al*, 2018). Protein palmitoylation is catalyzed by palmitoyl‐S‐acyl‐transferase (PAT) enzymes carrying a cysteine‐rich domain with a catalytic signature Asp‐His‐His‐Cys (DHHC) surrounded by four transmembrane domains. Notably, palmitoylation regulates the IMC targeting of several proteins, including ISP1 and ISP3 in *Plasmodium* schizonts (Wetzel *et al*, 2015); however, the upstream PAT(s) adding palmitate to the ISP proteins remains unknown. In this study, we show that both ISP1 and ISP3 have polarized expression in zygotes and that ISP1 and ISP3 polarization at the apical is critical for zygote‐to‐ookinete differentiation. The localization and function of ISP1 and ISP3 depend on palmitoylation of N‐terminal cysteines catalyzed by an IMC‐residing PAT DHHC2. Additionally, palmitoylation at DHHC2 C‐terminal cysteine catalyzed by its own PAT activity regulates the IMC localization of DHHC2. IMC‐anchored ISP proteins interact with the microtubule component β‐tubulin to maintain SPM structure integrity. This study discovers a protein palmitoylation cascade tethering cortical membrane with microtubule to maintain the proper cytoskeleton structure for zygote‐to‐ookinete differentiation.

## Results

### Disruption of *isp1* and *isp3* impairs zygote‐to‐ookinete differentiation

**Figure EV1:**
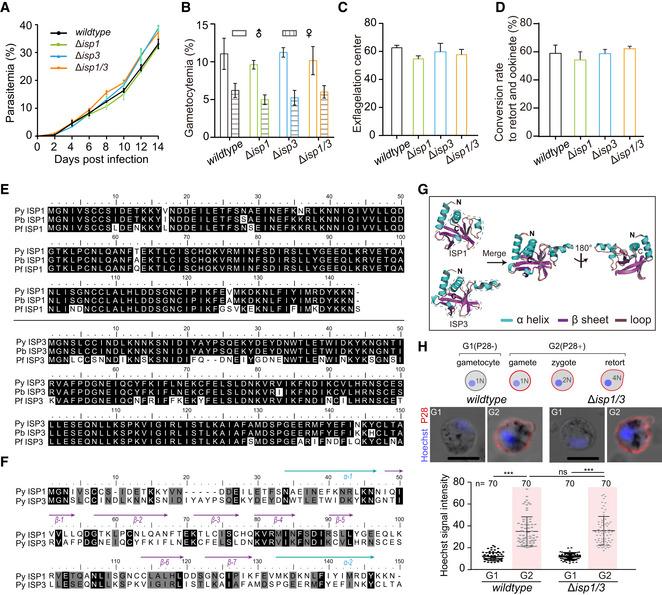
Development analysis of the Δ*isp1*, Δ*isp3*, and Δ*isp1/3* parasites and protein sequence analysis of ISP1 and ISP3 Parasitemia in mouse. Values are means ± SEM (*n* = 3 biological replicates).Gametocytemia in mouse. Values are means ± SEM (*n* = 3 biological replicates).Male gametocyte activation *in vitro* by counting the exflagellation centers formed. Values are means ± SEM (*n* = 3 biological replicates).
*In vitro* differentiation to retort and ookinete. Values are means ± SEM (*n* = 3 biological replicates).Alignment of ISP1 (up panel) or ISP3 (lower panel) protein sequences from *P. yoelii*,* P. berghei*, and *P. falciparum*. *pyisp1*: PY17X_1212600; *pbisp1*: PBANKA_1209400; *pfisp1*: PF3D7_1011000; *pyisp3*: PY17X_1328100; *pbisp3*: PBANKA_1324300; *pfisp3*: PF3D7_1460600.Second structure‐based sequence alignment of ISP1 and ISP3 protein of *P. yoelii*.Predicted structures of *P. yoelii* ISP1 and ISP3. The structures of *Plasmodium* ISP1 and ISP3 are constructed using homology modeling with SWISS‐MODEL based on protein structures of *T. gondii* ISP1 (PDB: 4chm) and ISP3 (PDB: 4chj). Both protein structures of ISP1 and ISP3 display a character of the pleckstrin homology (PH) domain (indicated by dashed line), composing one α‐helix and six β‐sheets. The α‐helix is shown in green, β‐sheet in purple, and loop in brown.Nuclei DNA content analysis of parasite. Upper panel indicates the schematic of female gametocyte–female gamete–zygote–retort/ookinete differentiation. One female gamete (1N) fertilizes with one male gamete to form zygote (2N) and further develop to retort/ookinete (4N) by meiotic DNA replication. P28 and Hoechst 33342 staining of female gametocyte, female gamete, zygote, and retort of WT and Δ*isp1/3* parasites. Zygotes and retorts were collected at 4 h post‐activation. Scale bar = 5 μm. Lower panel indicates the quantification of the Hoechst fluorescence signals. Values are mean ± SD (*n* is the number of cells measured in each group). Mann–Whitney test, ***P < 0.001. Three biological replicates performed.

**Figure 1:**
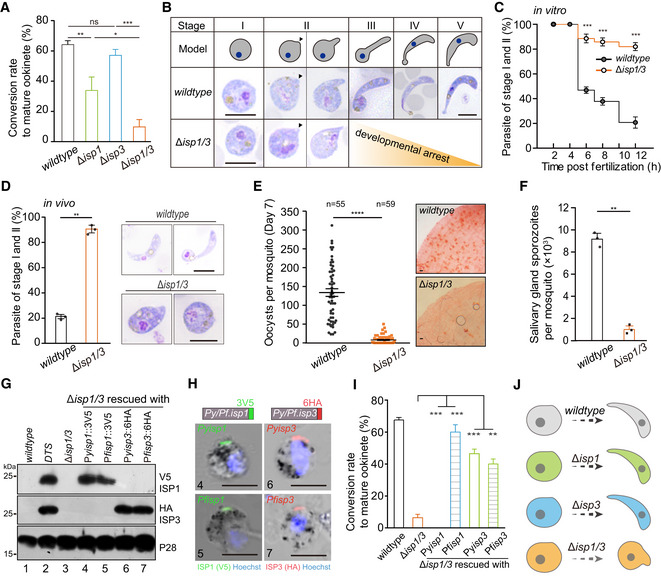
Parasites lacking both *isp1* and *isp3* genes have impaired zygote‐to‐ookinete differentiation *In vitro* differentiation to mature ookinete of wild type (WT), Δ*isp1*, Δ*isp3*, and Δ*isp1/3* parasites. Values are means ± SD (*n* = 4 biological replicates); two‐tailed *t*‐test, *P < 0.05, **P < 0.01, ***P < 0.001, ns: not significant.Giemsa staining of zygotes/ookinetes from *in vitro* culture. Upper diagrams indicate morphological changes from zygote to ookinete. Black arrow indicates the apical. Scale bar = 5 μm.Time‐course analysis of ookinete differentiation from *in vitro* culture. Values are means ± SEM (*n* = 3 biological replicates), two‐tailed *t*‐test, ***P < 0.001.
*In vivo* ookinete differentiation in midgut of the infected mosquitos. Right panel indicates the parasite smear stained with Giemsa solution. Values are means ± SEM (*n* = 3 biological replicates), two‐tailed *t*‐test, **P < 0.01. Scale bar = 5 μm.Number of oocysts in mosquito midgut 7 days post‐blood feeding. n is the number of mosquitoes in each group. The horizontal line shows the mean value of each group, Mann–Whitney test, ****P < 0.0001. Right panel shows mosquito midguts stained with 0.5% mercurochrome. Scale bar = 50 μm. Three biological replicates performed.Formation of salivary gland sporozoites in mosquitoes 14 days post‐blood feeding. In each group, at least twenty blood‐fed mosquitoes were counted. Values are means ± SEM (*n* = 3 biological replicates), two‐tailed *t*‐test, **P < 0.01.Western blot of ISP1 and ISP3 in zygote of the Δ*isp1/3* parasite episomally complemented with 3V5‐tagged *isp1* or 6HA‐tagged *isp3* genes from either *P. yoelii* or *P. falciparum*. P28 as loading control. The *DTS* (*isp1::3V5;isp3::6HA*) is a doubly tagged parasite described in Fig 2B.IFA analysis of tagged ISP1 and ISP3 in zygotes of the complemented parasites. Nuclei are stained with Hoechst 33342. Scale bar = 5 μm.
*In vitro* ookinete differentiation of the complemented parasites. Values are means ± SEM (*n* = 3 biological replicates), two‐tailed *t*‐test, **P < 0.01, ***P < 0.001.Proposed model for ISP1 and ISP3 during zygote‐to‐ookinete differentiation. Source data are available online for this figure.

To elucidate the function of *Plasmodium* ISP1 and ISP3 during ookinete development, we disrupted *isp1* or *isp3* genes in *P. yoelii* 17XNL by double cross‐over homologous replacement using the CRISPR/Cas9 method (Zhang *et al*, 2014, 2017; Appendix Fig S1). Both *∆isp1* and *∆isp3* mutants showed normal development of asexual blood stages and gametocytes *in vivo* as well as gamete formation and zygote‐to‐retort differentiation *in vitro* (Fig EV1A–D). However, the mature ookinete conversion *in vitro* was significantly reduced in *∆isp1* (conversion rate: 34%) but not in *∆isp3* (57%) compared with wild type (WT, 64%; Fig 1A). Both ISP1 and ISP3 proteins are well‐conserved among *P. yoelii, P. berghei,* and *P. falciparum* parasites (Fig EV1E). Additionally, *P. yoelii* ISP1 and ISP3 also share conserved motifs (Fig EV1F) and have similar predicted 3D structures modeled on protein structures of *Toxoplasma gondii* ISP1 and ISP3, respectively (Tonkin *et al*, 2014; Fig EV1G). The structural similarity between ISP1 and ISP3 suggests potential functional overlaps between these ISP proteins. Therefore, we further disrupted *isp3* in the *∆isp1* parasite and generated a double knockout *∆isp1/3* mutant (Appendix Fig S1). Indeed, the mature ookinete conversion rate of this *∆isp1/3* parasite (9%) was significantly lower than that of *∆isp1* or *∆isp3* (Fig 1A).

Measurement of DNA content after Hoechst 33342 stain indicated the P28‐positive gametes of the *∆isp1/3* parasite could be fertilized and further develop from diploid to tetraploid, suggesting normal DNA replication (Fig EV1H). However, time‐course (stages I–V) analysis of ookinete differentiation *in vitro* revealed developmental arrest at early stages (I and II) for the *∆isp1/3* cell (Fig 1B and C). We also isolated parasites from infected mosquito midguts and observed a similar defect of *∆isp1/3 in vivo* (Fig 1D). Consequently, the *∆isp1/3* parasite showed significantly reduced number of day 7 midgut oocysts and day 14 salivary gland sporozoites compared with the WT in the infected mosquitoes (Fig 1E and F). Together, these results demonstrate that loss of both ISP1 and ISP3 causes a severe defect in early ookinete differentiation and therefore mosquito transmission of the parasite.

To rule out the possibility that the defective ookinete phenotype was caused by the Cas9 off‐target effects, we complemented the *∆isp1/3* parasite by episomal expression of V5‐tagged PyISP1 (from *P. yoelii*) or PfISP1 (from the human malaria pathogen *P. falciparum*). Expression of V5‐tagged PyISP1 or PfISP1 was detected in zygotes of the complemented parasites by Western blotting (Fig 1G) or indirect immunofluorescence assay (IFA; Fig 1H). Both proteins had polarized localization at one site at the zygote cell periphery (Fig 1H), in agreement with the expression pattern of the endogenous proteins (see below). Notably, both complemented parasites regained the normal level of mature ookinete formation (Fig 1I), demonstrating that the ookinete developmental defect was indeed due to the disruption of ISP1/ISP3. These results also show functional exchangeability of ISP proteins between *Plasmodium* spp. We also complemented the *∆isp1/3* parasite with HA‐tagged PyISP3 and PfISP3, respectively. Both PyISP3 and PfISP3 were capable of rescuing the ookinete developmental defect of *∆isp1/3*, although the ookinete conversion rates were still lower than that of WT parasites (Fig 1I). These results demonstrate critical roles of ISP1 and ISP3 in early development during zygote‐to‐ookinete differentiation (Fig 1J).

### Polarized co‐localization of ISP1 and ISP3 at zygote apical patch

**Figure EV2:**
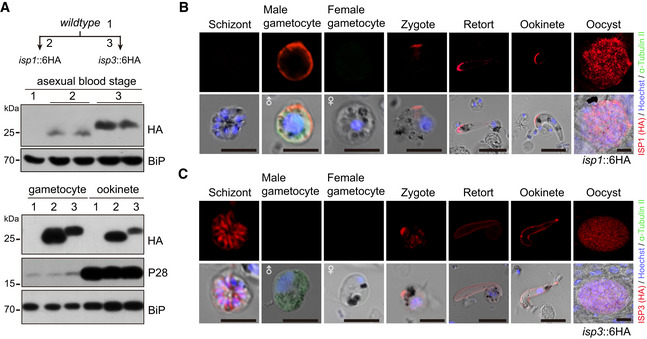
Stage expression and cellular localization of ISP1 and ISP3 AWestern blot of ISP1 and ISP3 in asexual mouse blood stages, gametocytes, and ookinetes of the *isp1::6HA* and *isp3::6HA* parasites. ER protein BiP as loading control. Two lanes in blot are replicates from the same sample.B, CIFA of ISP1 (B) and ISP3 (C) in mouse and mosquito stages of the *isp1::6HA* and *isp3::6HA* parasites, respectively. Purified gametocytes were stained with antibodies against HA and *α*‐tubulin II (male gametocyte‐specific). Scale bar = 5 μm. Source data are available online for this figure.

**Figure 2:**
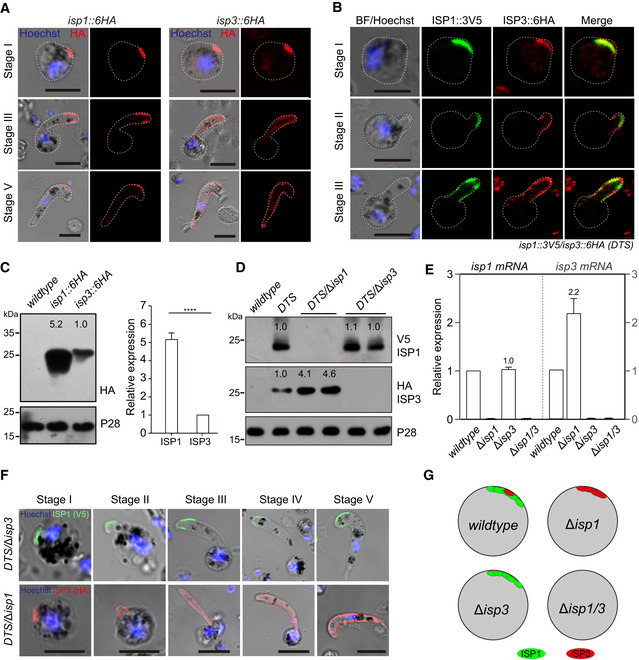
Polarized co‐localization of ISP1 and ISP3 at zygote apical patch IFA of ISP1 and ISP3 expression from zygote to ookinete of the *isp1::6HA* and *isp3::6HA* parasites, respectively. Scale bar = 5 μm.Co‐localization of ISP1 and ISP3 from zygote to ookinete in the doubly tagged strain (*DTS, isp1::3V5;isp3::6HA*). Scale bar = 5 μm.Western blot of ISP1 and ISP3 expression in zygotes of *isp1::6HA* and *isp3::6HA* parasites. The numbers are the relative intensities of band in blot. Right panel: the quantification of band intensity. Values are means ± SEM (*n* = 4 biological replicates), two‐tailed *t*‐test, ****P < 0.0001.Western blot of ISP1 and ISP3 expression in zygotes of *DTS*,* DTS*/Δ*isp1*, and *DTS*/Δ*isp3* parasites. The numbers are the relative intensities of band in blot. Three replicates performed.RT–qPCR analysis of *isp1* and *isp3* transcripts in zygotes of the WT, Δ*isp1*, Δ*isp3*, and Δ*isp1/3* parasites. The expression is normalized to the *18S rRNA*. Values are means ± SEM (*n* = 4 biological replicates).IFA analysis of ISP1 and ISP3 expression dynamic of *DTS*/Δ*isp1* and *DTS*/Δ*isp3* parasites. Scale bar = 5 μm.Proposed model of polarized co‐localization and expression compensation between ISP1 and ISP3. Source data are available online for this figure.

To confirm the polarized localization of ISP1 and ISP3 in the zygote as seen in Fig 1H, we tagged individual endogenous ISP1 or ISP3 with a sextuple HA epitope (6HA) at the C‐terminus, obtaining the *isp1::6HA* and *isp3::6HA* parasites (Appendix Fig S1). Both proteins were expressed in asexual blood stages, gametocytes, ookinetes, and oocysts (Fig EV2A–C). Time‐course expression analysis showed that both proteins were concentrated at an apical dot in zygotes (stage I), with residual ISP3 distributed at cytoplasm (Fig 2A). In stage III, both proteins are expressed at the periphery of the protrusion, but not zygote remnant (Fig 2A). These results support that ISP1/ISP3 is localized in IMC, but not PPM during this differentiation, in agreement with previous observations in *P. berghei* (Poulin *et al*, 2013). Mature ookinetes (stage V) still had peripheral distribution of both ISP1 and ISP3 proteins in whole cell (Fig 2A). We further generated a doubly tagged parasite (*DTS*), *isp1::3V5/isp3::6HA*, by tagging endogenous ISP1 with a triple V5 epitope (3V5) in the *isp3::6HA* parasite (Appendix Fig S1) and observed similar co‐localization of ISP1/ISP3 at the periphery of zygote protrusion (Fig 2B). The localization patterns of ISP1/ISP3 further suggest critical roles of these two proteins in zygote protrusion and/or elongation.

### Compensation of gene expression between *isp1* and *isp3*

To differentiate the contributions of ISP1 and ISP3 to ookinete development, we quantified the relative expression level of ISP1 and ISP3 in zygotes. Western blot analysis of protein extracts from the same amounts of zygotes of *isp1::6HA* and *isp3::6HA* revealed approximately fivefold higher ISP1 protein level than that of ISP3 in zygotes (Fig 2C). Given the facts that depletion of ISP1 or ISP3 alone causes modest defect (Fig 1A), we reasoned that ISP3 expression might be increased in the absence of ISP1. Therefore, we disrupted *isp1* in *DTS* parasite and found that ISP1 depletion indeed resulted in approximately fourfold increase in ISP3 protein level in the *DTS/*∆*isp1* zygotes (Fig 2D). In contrast, ISP3 depletion has no effect on ISP1 expression in zygote of the *DTS/*∆*isp3* (Fig 2D). Removal of ISP1 did not affect distribution dynamics of ISP3 during ookinete differentiation, and vice versa (Fig 2F), indicating the localization of ISP1 and ISP3 is mutually independent. We performed quantitative RT–PCR to investigate whether ISP3 compensation occurred at transcriptional level. Indeed, ISP1 disruption resulted in more than twofold increase in *isp3* mRNA transcript, whereas ISP3 depletion had no effect on *isp1* transcription (Fig 2E). Therefore, *isp3* is capable of compensating the loss of *isp1* by increasing its transcription (Fig 2G), which may partially explain the severe defect in *∆isp1/3*, but not *∆isp1* or *∆isp3*.

### Palmitoylation is critical for IMC localization and function of ISP1/ISP3

**Figure 3:**
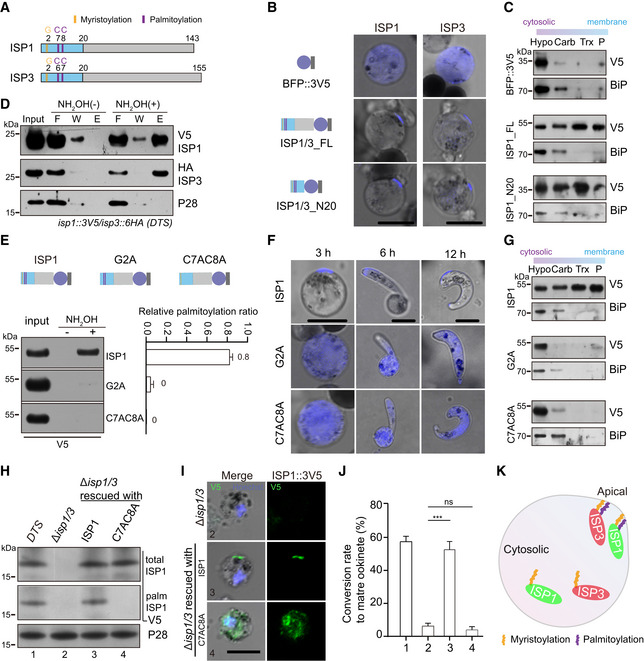
Palmitoylation is critical for ISP1/ISP3 polarization and ookinete differentiation Structure and N‐terminal residues for potential acylation of ISP1 and ISP3.Fluorescent microscopy of ISP and its N‐terminal 20 residues (N20) fused with a BFP::3V5 peptide episomally expressed in zygotes. Scale bar = 5 μm.Solubility assay detecting membrane association of ISP1_FL and ISP1_N20 using different detergents. Cytosolic soluble proteins are in hypotonic buffer (Hypo), peripheral membrane proteins in carbonate buffer (Carb), integral membrane proteins in Triton X‐100 buffer (Trx), and insoluble proteins in pellet (P). BFP::3V5 is in the soluble fraction, while ISP1_FL and ISP1_N20 are in the membrane‐associated fractions. ER protein BiP is a loading control.Acyl‐RAC method detecting palmitoylation of ISP1 and ISP3 in zygotes of the *DTS* strain. Proteins from F (flow‐through), W (wash), and E (elution) are analyzed. P28 as a loading control.Substitutions of N‐terminal cysteine or glycine residues to alanine (G2A or C7A/C8A) ablate palmitoylation of ISP1. ISP1 was fused with BFP::3V5 and episomally expressed in zygotes. Right panel shows quantification of palmitoylated protein band from two independent repeats, and values are means ± SD.G2A and C7AC8A mutations cause cytoplasmic distribution of ISP1 during zygote‐to‐ookinete differentiation. Scale bar = 5 μm.Membrane association of ISP1 with either G2A or C7AC8A substitutions.Expression and palmitoylation of ISP1 in ∆*isp1/3* parasites complemented with the 3V5‐tagged WT ISP1 or C7AC8A mutant.IFA of ISP1 at zygote of the complemented parasites. Scale bar = 5 μm.
*In vitro* differentiation to mature ookinete of the complemented parasites. Values are means ± SEM (*n* = 4 replicates), two‐tailed *t*‐test, ***P < 0.001, ns: not significant.A model illustrating palmitoylation critical for apical localization of ISP1 and ISP3 at zygote. Source data are available online for this figure.

We next investigated how ISP1 and ISP3 are directed to IMC. ISP1 and ISP3 are small proteins with 143 and 155 amino acids (aa), respectively (Figs 3A and EV1E and F). The N‐terminal 20 aa (N20) of both *P. falciparum* ISP1 and ISP3 has been shown to be sufficient for targeting the proteins to the IMC in schizonts (Wetzel *et al*, 2015). Episomal expression of BFP::3V5 peptide fused with full‐length ISP1 (ISP1_FL) or N20 of ISP1 (ISP1_N20) driven by *Pyisp1* promoter showed typical polarized localization in zygotes, while BFP::3V5 alone was expressed in the cytoplasm (Fig 3B). Using detergent extraction‐based protein solubility assay (Cabrera *et al*, 2012; Wetzel *et al*, 2015), the ISP1_FL and ISP1_N20 were detected in fractions associated with membrane or cytoskeleton, whereas BFP::3V5 was found exclusively in soluble fraction (Fig 3C). Similarly, the N20 also directed IMC localization of ISP3 in zygotes (Fig 3B). Therefore, the IMC localization signals in zygote and ookinete are within the N20 peptides of ISP1 and ISP3.

Both ISP1 and ISP3 have two N‐terminal cysteines (Fig 3A, C7/C8 in ISP1 and C6/C7 in ISP3) that can potentially be palmitoylated for IMC targeting as shown in schizonts of *P. falciparum* (Wetzel *et al*, 2015). Using resin‐assisted capture (Acyl‐RAC) method (Forrester *et al*, 2011), we detected palmitoylation of ISP1 and ISP3 in the *DTS* zygotes (Fig 3D). To study the effect of C7/C8 palmitoylation on protein IMC localization, we generated an ISP1 mutant by replacing C7/C8 with A7/A8 (alanine) and fused the mutant ISP1 with BFP::3V5 reporter. The C7A/C8A mutant protein lost palmitoylation and was localized in the cytoplasm when episomally expressed in WT parasites (Fig 3E and F, Appendix Fig S2). In addition, glycine‐to‐alanine substitution at position 2 (G2A) abolished ISP1 palmitoylation and IMC localization (Fig 3E and F; Appendix Fig S2), supporting the observations that glycine myristoylation enhances palmitoylation of the adjacent cysteine (Aicart‐Ramos *et al*, 2011). The change in subcellular localization of these palmitoylation‐deficient proteins was also supported by the loss of protein association with membrane in solubility assays (Fig 3G). To test the physiological role of ISP1 palmitoylation in ookinete differentiation, we complemented the *∆isp1/3* parasite with WT ISP1 or palmitoylation‐deficient ISP1^C7A/C8A^ via episomal expression (Fig 3H and I). Parasites complemented with WT ISP1 had normal protein polarized localization and ookinete differentiation, but parasites complemented with the C7A/C8A mutations did not (Fig 3J), confirming an essential role of palmitoylation on IMC targeting of ISP1 and ookinete differentiation. Together, these data demonstrate that palmitoylation of N‐terminal cysteines is a critical PTM that regulates the IMC localization and proper function of both ISP1 and ISP3 (Fig 3K).

### DHHC2 associates with ISP1/ISP3 in a polarity patch of zygote

**Figure EV3:**
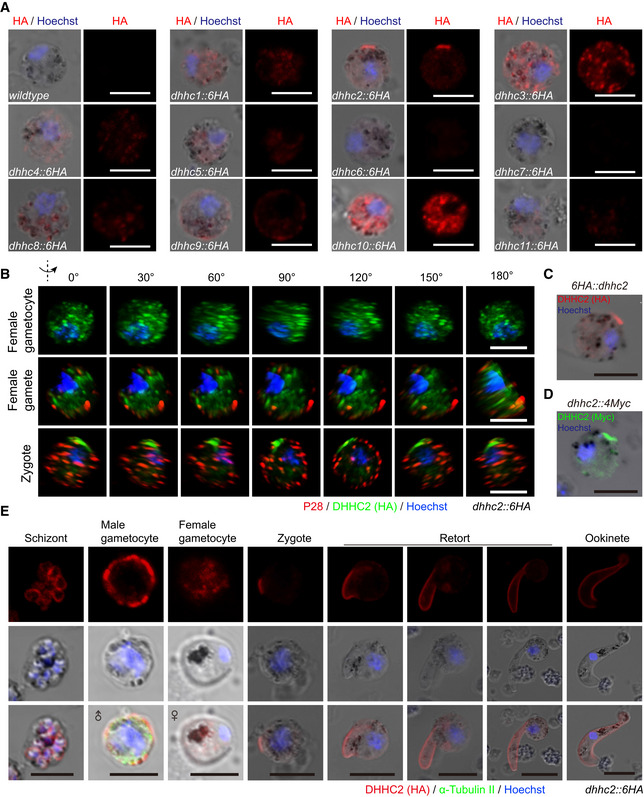
Screening and identification of DHHC2 polarizing at zygotes A
IFA of eleven *Plasmodium* palmitoyl‐S‐acyl‐transferase (PAT) proteins in zygotes. Endogenous protein is tagged C‐terminally with a 6HA tag. Only DHHC2 displays polarization at zygote of the *dhhc2::6HA* parasites. Scale bar = 5 μm.
B3D imaging of DHHC2 and P28 expression in female gametocyte, female gamete, and zygotes of the *dhhc2::6HA* parasite. Scale bar = 5 μm.C, DIFA of DHHC2 expression in zygotes of another two independent strains *6HA::dhhc2* and *dhhc2::4Myc*. Scale bar = 5 μm.EIFA of DHHC2 expression in asexual blood stages, gametocytes, and zygote to ookinete of the *dhhc2::6HA* parasite. Purified gametocytes were stained with antibodies against HA and *α*‐tubulin II (male gametocyte‐specific). Scale bar = 5 μm.

**Figure 4:**
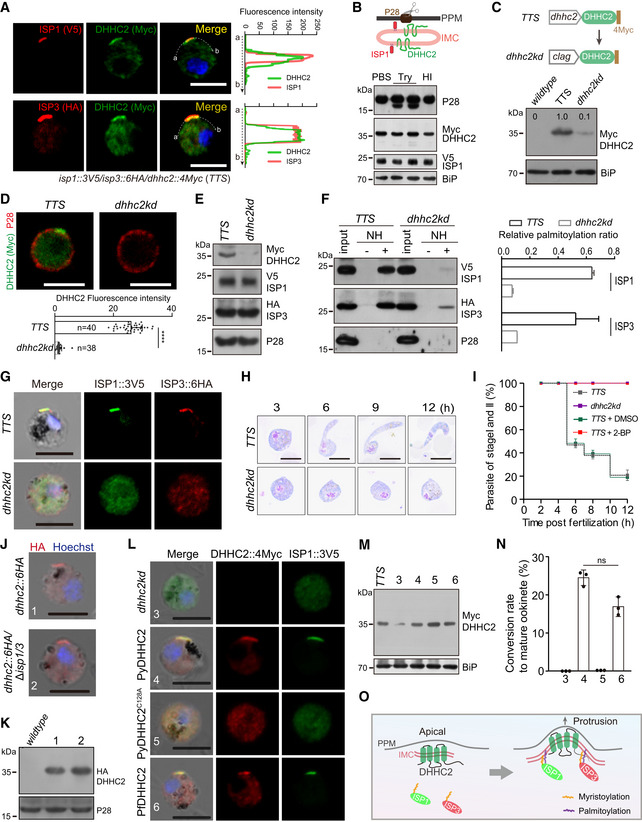
DHHC2 directs ISP1/3 palmitoylation and ookinete differentiation DHHC2 directs ISP1/3 palmitoylation and ookinete differentiation Two‐colored IFA of ISP1/DHHC2 and ISP3/DHHC2 at zygotes of the triple‐tagged strain *isp1*::*3V5/isp3*::*6HA/dhhc2::4Myc* (*TTS*). Right graph shows overlay of fluorescence peaks along cell periphery. Scale bar = 5 μm.Western blot of DHHC2, ISP1, and P28 (plasma membrane protein) of the *TTS* zygotes treated with PBS, trypsin (Try), or heat‐inactivated (HI) trypsin. Upper panel shows the PPM and IMC structures.Knockdown of *dhhc2* expression. Upper panel shows the promoter swap strategy applied in *TTS* strain, generating the *dhhc2kd* mutant with endogenous *dhhc2* promoter replaced with *clag* promoter. Western blot confirming decreased expression of DHHC2 in *dhhc2kd* zygotes. Three replicates performed.Two‐colored IFA of DHHC2 and P28 proteins in the *TTS* and *dhhc2kd* zygotes. Scale bar = 5 μm. Bottom panel, quantifications of DHHC2 fluorescence. Values are means ± SD (*n*: the number of cells analyzed). Mann–Whitney test, ****P < 0.0001.Western blot of DHHC2, ISP1, and ISP3 in the *TTS* and *dhhc2kd* zygotes. Two replicates performed.Palmitoylation of ISP1 and ISP3 in the *TTS* and *dhhc2kd* zygotes using Acyl‐RAC method. NH: NH_2_OH. Right panel indicates quantification of protein band intensity from three replicates, and values are means ± SD.IFA of ISP1 and ISP3 in zygotes of *TTS* and *dhhc2kd*. Scale bar = 5 μm.Giemsa staining of zygote/ookinete from *in vitro*‐cultured *TTS* and *dhhc2kd* parasites. Scale bar = 5 μm.Time‐course analysis of *in vitro* ookinete differentiation. Values are means ± SD from three independent tests.IFA of DHHC2 expression in the *dhhc2::6HA* and *dhhc2::6HA;*∆*isp1/3* zygotes. Scale bar = 5 μm.Western blot of DHHC2 in zygotes of *dhhc2::6HA* and *dhhc2::6HA;*∆*isp1/3*.IFA of DHHC2 and ISP1 in zygotes of *dhhc2kd* parasites complemented with 4Myc‐tagged WT PyDHHC2 of *P. yoelii*, PfDHHC2 of *P. falciparum*, or PyDHHC2 with PAT catalytic‐deficient mutation C128A. Scale bar = 5 μm.Western blot of DHHC2 in the complemented *dhhc2kd* parasites as indicated.
*In vitro* differentiation to mature ookinetes of the complemented parasites. Values are means ± SD from three independent tests, two‐tailed *t*‐test, ns: not significant.Model of DHHC2‐mediated palmitoylation of ISP1 and ISP3 in zygote. Source data are available online for this figure.

Next, we searched for the PATs that palmitoylate ISP1 and ISP3. PATs generally co‐localize with their substrate proteins (Daniotti *et al*, 2017; Rana *et al*, 2018). There are 11 predicted PATs (named DHHC1–11) in the genomes of *P. yoelii* and *P. berghei* parasites (Seydel *et al*, 2005; Hodson *et al*, 2015). We tagged all 11 *P. yoelii* endogenous PATs with 6HA and analyzed their localization in zygotes (Appendix Fig S1). Out of the 11 PATs, only DHHC2 showed polarized expression with minor cytoplasmic distribution in the *dhhc2::6HA* parasite zygotes (Fig EV3A). The polarized localization of DHHC2 was confirmed in 3D imaging of the *dhhc2::6HA* zygotes (Fig EV3B) and observed in two other independent parasites, *6HA::dhhc2* (DHHC2 tagged with N‐terminal 6HA) and *dhhc2::4Myc* (DHHC2 tagged with C‐terminal 4Myc) (Fig EV3C and D). To confirm co‐localization of DHHC2 and ISP1/ISP3, we tagged the endogenous DHHC2 with 4Myc in the *DTS* parasite, generating the triple‐tagged parasite *isp1*::*3V5/isp3*::*6HA/dhhc2::4Myc* (*TTS*) (Appendix Fig S1). DHHC2 was indeed co‐localized with ISP1 and ISP3 at a polarity patch of zygote (Fig 4A).

Similar to ISP1/ISP3, DHHC2 was expressed only at periphery of the protrusion and elongation where IMC is assembled, but not at zygote remnant during zygote‐to‐ookinete differentiation (Fig EV3E), suggesting that DHHC2, ISP1, and ISP3 are localized at IMC, but not at PPM. DHHC2 possesses typical four transmembrane domains surrounding the DHHC motif, with both N‐ and C‐termini facing the cytoplasm. We treated the *TTS* zygotes with trypsin to digest extracellular parts of proteins residing across PPM. A digested product of the PPM protein P28 but not DHHC2 or ISP1 was detected (Fig 4B). These results indicate that DHHC2 and ISP1/ISP3 are not exposed on the PPM surface, further suggesting the IMC localization of DHHC2 and ISP1/ISP3.

### DHHC2 palmitoylates ISP1/ISP3 proteins

**Figure EV4:**
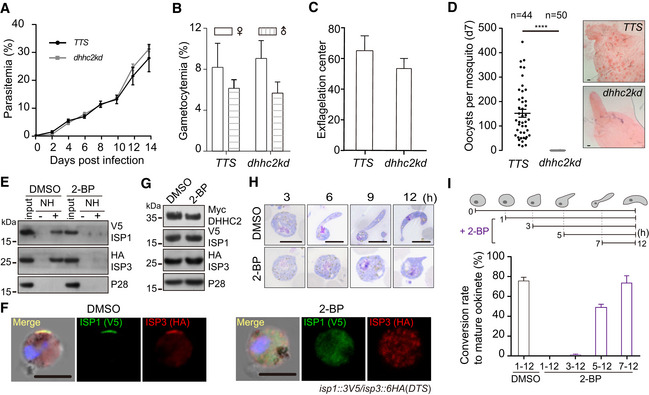
DHHC2 palmitoylates ISP1 and ISP3 Parasitemia in mouse. Values are means ± SEM (*n *= 3 biological replicates).Gametocytemia in mouse. Values are means ± SEM (*n* = 3 biological replicates).Male gametocyte activation *in vitro*. Values are means ± SEM (*n* = 6 biological replicates).Midgut oocysts in mosquitoes 7 days post‐blood feeding. n is the number of mosquitoes in each group. The horizontal line shows the mean value of each group, Mann–Whitney test, ****P < 0.0001. Right panel shows mosquito midguts stained with 0.5% mercurochrome. Scale bar = 50 μm. Three biological replicates performed.Acyl‐RAC method detecting palmitoylation of ISP1 and ISP3 in *DTS* zygotes treated with 100 μΜ 2‐BP, a potent inhibitor of protein palmitoylation. NH, NH_2_OH. The data are representative of two repeats.IFA of ISP1 and ISP3 in 2‐BP‐treated *DTS* zygotes. Scale bar = 5 μm.Western blot of DHHC2, ISP1, and ISP3 in 2‐BP‐treated *TTS* zygotes.Time‐course analysis of ookinete differentiation treated with 2‐BP. Scale bar = 5 μm. h, hour.Inhibiting effect of 2‐BP on ookinete differentiation is time‐dependent. 2‐BP treatment during early development (1–3 h) damages the ookinete differentiation. Values are means ± SEM (*n* = 3 biological replicates). Source data are available online for this figure.

To prove the role of DHHC2 in palmitoylating ISP1/ISP3, we attempted to disrupt the *dhhc2* gene but failed to obtain a viable clone, suggesting an essential role in asexual blood stage of *P. yoelii*, in agreement with previous reports in *P. berghei* (Santos *et al*, 2015; Bushell *et al*, 2017). We used alternative strategy to knockdown DHHC2 expression by replacing endogenous *dhhc2* promoter (573 bp) with the promoter (1,809 bp) from *clag1* (PY17X_1402200) gene in the *TTS* line (Fig 4C, Appendix Fig S1). The Clag1 protein is highly expressed in asexual blood stages, but is absent in gametocytes and mosquito stages including zygotes (Sebastian *et al*, 2012). Promoter replacement significantly reduced the level of DHHC2 protein in zygotes of the resulted parasite *dhhc2kd* (Figs 4C and D). The *dhhc2kd* parasite proliferated in mouse blood, produced functional male and female gametocytes (Fig EV4C), expressed ISP1 and ISP3 at levels comparable to those of the *TTS* parasite (Fig 4E), but had significantly reduced palmitoylation on ISP1 and ISP3 in zygotes (Fig 4F). Consequently, ISP1 and ISP3 lost polarized localization in the *dhhc2kd* zygotes (Fig 4G). These results indicate that DHHC2 is the major PAT palmitoylating ISP1/ISP3. The *dhhc2kd* parasite displayed a developmental arrest at early stages with no formation of mature ookinetes (Fig 4H and I), repeating the phenotype of *dhhc2kd* knockdown in *P. berghei* (Santos *et al*, 2015). No oocyst was observed in midguts from mosquitoes infected with the *dhhc2kd* parasites (Fig EV4D). Additionally, treatment of the *TTS* zygote culture with 100 μΜ 2‐bromopalmitate (2‐BP), an inhibitor of protein palmitoylation (Jennings *et al*, 2009), also impaired the palmitoylation and proper localization of ISP1 and ISP3 (Fig EV4E and F), but did not affect the protein level of ISP1 and ISP3 (Fig EV4G). 2‐BP treated parasites completely arrested ookinete differentiation at early stages (Figs 4I and EV4H), resembling the defect of *dhhc2kd* (Figs 4H). Interestingly, the defect caused by 2‐BP treatment was time‐dependent; 5 h after XA stimulation of gametocyte culture, 2‐BP had little effect on ookinete differentiation (Fig EV4I). In addition, depletion of both ISP1/ISP3 had no impact on IMC targeting and protein abundance of DHHC2 in zygotes of the *dhhc2::6HA/∆isp1/3* parasite (Figs 4J and K), indicating that the IMC localization of DHHC2 is ISP‐independent in zygotes.

To further test whether DHHC2 is the enzyme responsible for ISP1 and ISP3 palmitoylation, we introduced DHHC2 from either *P. yoelii* or *P. falciparum* back to the *dhhc2kd* parasite by episomal expression. Both PyDHHC2 and PfDHHC2 proteins showed polarized localization and restored the polarized localization of ISP1 in zygotes of the complemented parasites (Fig 4L and M). Importantly, the parasites regained the ability to differentiate into mature ookinetes (Fig 4N). The results also indicated conserved function between *P. falciparum* and *P. yoelii* DHHC2 enzymes. Replacing cysteine to alanine within DHHC motif impairs the catalytic activity of PATs (Fukata & Fukata, 2010). Compared to WT DHHC2, the catalytic‐deficient DHHC2^C128A^ failed to restore zygote polarization of ISP1 (Fig 4L and M) as well as ookinete differentiation of the *dhhc2kd* (Fig 4N), indicating that the PAT activity is essential for DHHC2 function. Surprisingly, this C128A mutation in the DHHC motif led to cytoplasmic distribution of DHHC2 (Fig 4L), indicating that its own PAT activity is also required for the proper localization of DHHC2. Together, these results demonstrate that the palmitoylation of ISP1/ISP3 by DHHC2 mediates their IMC targeting, which is essential for zygote‐to‐ookinete differentiation (Fig 4O).

### Palmitoylation of C‐terminal cysteines controls DHHC2 IMC localization

**Figure 5:**
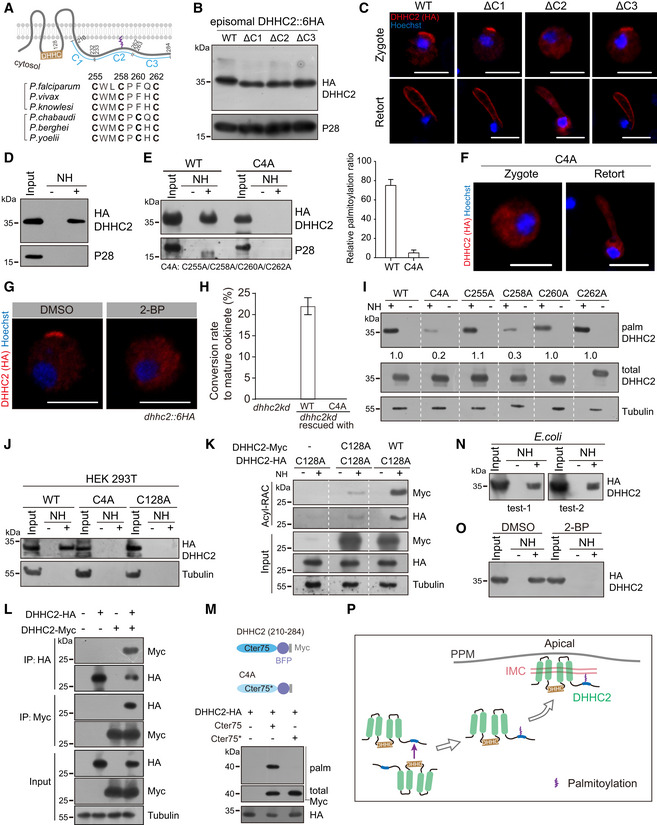
Cysteine palmitoylation in cytosolic C‐terminus, catalyzed by its own PAT activity, directs IMC targeting of DHHC2 Topology and cytosolic C‐terminus (210–284 residues) of DHHC2. Lower panel indicates the conserved cysteines (residues 255, 258, 260, and 262) of DHHC2 from six *Plasmodium* species.Western blot of episomally expressed DHHC2::6HA or variants containing truncated segment (C1:210–232, C2:233–262, C3:263–284 residues) in WT zygotes.IFA of episomally expressed DHHC2::6HA and three truncation mutants in zygotes and retorts. Scale bar = 5 μm.Acyl‐RAC method detecting palmitoylation of endogenous DHHC2 in the *dhhc2::6HA* zygotes.Palmitoylation of DHHC2::6HA and C4A mutant episomally expressed in zygotes. C4A: substitutions of C‐terminal four cysteine to alanine. Right panel indicates the quantification of protein band intensity from two replicates, and values are means ± SD.Cytoplasmic distribution of C4A mutant during zygote‐to‐ookinete differentiation. Scale bar = 5 μm.IFA of DHHC2 at the *dhhc2::6HA* zygotes treated with 100 μM 2‐BP. Scale bar = 5 μm.
*In vitro* ookinete differentiation of the *dhhc2kd* parasites complemented with either WT DHHC2 or C4A mutant. Values are means ± SD from three independent tests.Palmitoylation of the episomally expressed DHHC2::6HA with mutation in respective individual cysteine (C255A, C258A, C260A, and C262A). Representative of three independent repeats.Palmitoylation of HA‐tagged and human codon‐optimized DHHC2 ectopically expressed in human HEK293T cells. Both C4A and C128A mutations ablate DHHC2 palmitoylation.DHHC2_C128A‐HA mutant protein could be palmitoylated by the co‐transfected DHHC2WT‐Myc, but not DHHC2_C128A‐Myc in HEK293T cells. Representative of two independent repeats.Co‐immunoprecipitation of the DHHC2_WT‐HA and DHHC2_WT‐Myc co‐expressed in HEK293T cells.C‐terminus 75 residues peptide (Cter75) of DHHC2 could be palmitoylated as an independent substrate by co‐transfected DHHC2WT‐HA in HEK293T cells. Cter75 is fused with a BFP::Myc for easy detection via Western blot. Cter75* peptide contains C4A mutation.Palmitoylation of tagged DHHC2 ectopically expressed in bacteria *E. coli*.Palmitoylation of DHHC2 ectopically expressed in bacteria treated with 2‐BP.Model showing self‐palmitoylation of DHHC2 is catalyzed by its own PAT activity, which directs proteins for IMC targeting at zygotes. Source data are available online for this figure.

DHHC2 possesses a 75 aa (residue 210–284) cytosolic C‐terminus conserved among *Plasmodium* spp (Appendix Fig S3A). Hydrophilicity analysis revealed high hydrophobicity in a segment of residue 251–261 (Appendix Fig S3B). To test whether this C‐terminus regulates DHHC2 localization at zygote, we episomally expressed three 6HA‐tagged DHHC2, each with a truncated C‐terminus of different lengths (∆C1: missing residues 210–232, ∆C2: missing 233–262, and ∆C3 missing: 263–284) (Fig 5A). The expression of all three mutants was comparable to that of WT in zygotes (Fig 5B). However, only ∆C2 lost IMC targeting and polarized expression (Fig 5C), suggesting that the C2 segment is required for proper DHHC2 trafficking. Sequence alignment revealed four conserved cysteine residues (C255, C258, C260, and C262) in C2 segment (Fig 5A), and palmitoylation of these cysteines likely plays a role in IMC targeting of DHHC2. Using the Acyl‐RAC method, we indeed detected palmitoylation in both endogenous DHHC2 and episomally expressed DHHC2 in zygotes (Fig 5D and E). Importantly, replacement of all four cysteine with alanine (C255A/C258A/C260A/C262A: C4A) completely abolished the palmitoylation of DHHC2 (Fig 5E), leading to cytoplasmic distribution of the DHHC2 in zygotes and retorts (Fig 5F). Similarly, 2‐BP treatment also impaired the polarized localization of DHHC2 in the *dhhc2::6HA* zygotes (Fig 5G). These results indicate that palmitoylation of the C‐terminal cysteines is responsible for targeting DHHC2 to IMC in zygote and ookinete. We complemented the *dhhc2kd* by episomal expression of a C4A mutant protein and found that C4A could not rescue the defect of *dhhc2kd* mutant (Fig 5H), confirming the critical role of palmitoylation in DHHC2 function. To identify the palmitoylated cysteines, we generated C255A, C258A, C260A, and C262A plasmids by replacing the respective individual cysteine with alanine. The result showed that only C258A had decreased DHHC2 palmitoylation when episomally expressed in WT parasites (Fig 5I), suggesting that C258 is the main residue for DHHC2 palmitoylation and IMC targeting at zygote.

### Palmitoylation of DHHC2 is catalyzed by its own PAT activity

Since both PAT activity and palmitoylation of C‐terminal cysteines are required for proper localization of DHHC2, we hypothesize that DHHC2 undergoes self‐palmitoylation catalyzed by its own PAT activity. To test this, we transfected a construct encoding HA‐tagged human codon‐optimized DHHC2 (DHHC2‐HA) into human HEK293T cells. Palmitoylation was detected in this ectopically expressed DHHC2‐HA but not DHHC2 with the C4A mutation in HEK293 cells (Fig 5J). These results in human cells repeated the observations of DHHC2 activity in *Plasmodium* (Fig 5D and E). Moreover, the PAT catalytic‐deficient C128A mutant protein also lost palmitoylation, suggesting that its own PAT activity contributes to the cysteine palmitoylation of DHHC2 (Fig 5J). Indeed, this DHHC2/C128A‐HA protein could be palmitoylated by a co‐transfected WT DHHC2‐Myc in HEK293T (Fig 5K). In contrast, the DHHC2/C128A‐Myc mutant displayed reduced catalytic activity with a weak band of palmitoylation for DHHC2/C128A‐HA detected in two independent Western blots (Fig 5K), further confirming that DHHC2 is self‐palmitoylated by its own PAT activity. Consistent with the fact that PATs physically interact with their substrate, WT DHHC2‐HA and WT DHHC2‐Myc were mutually pulled down in co‐immunoprecipitation experiments (Fig 5L). Next, we asked whether the C‐terminal 75 residues (Cter75) of the DHHC2 could be palmitoylated as an independent substrate. Indeed, the Cter75 fused with a BFP::Myc peptide could be palmitoylated by co‐transfected WT DHHC2‐HA in HEK293 cells, while the Cter75 containing C4A mutations (Cter75*) failed to be modified (Fig 5M).

To further confirm the self‐palmitoylation of DHHC2 via its own PAT activity, the HA‐tagged DHHC2 was ectopically expressed in the bacteria *E. coli* where no endogenous PAT enzymes are encoded (Yadav *et al*, 2019). Palmitoylation was detected in the recombinant DHHC2 from total proteins of bacteria (Fig 5N), and this modification was abolished in bacterial culture treated with 2‐BP (Fig 5O). Taken together, these results indicate that DHHC2 undergoes self‐palmitoylation via its own PAT activity (Fig 5P). To the best of our knowledge, the *Plasmodium* DHHC2 is the first identified PAT enzyme possessing self‐palmitoylation outside the cysteine‐rich domain to regulate its subcellular localization.

### Cytoplasmic palmitoylated DHHC2 translocates to newly assembled IMC in fertilized zygote

**Figure 6:**
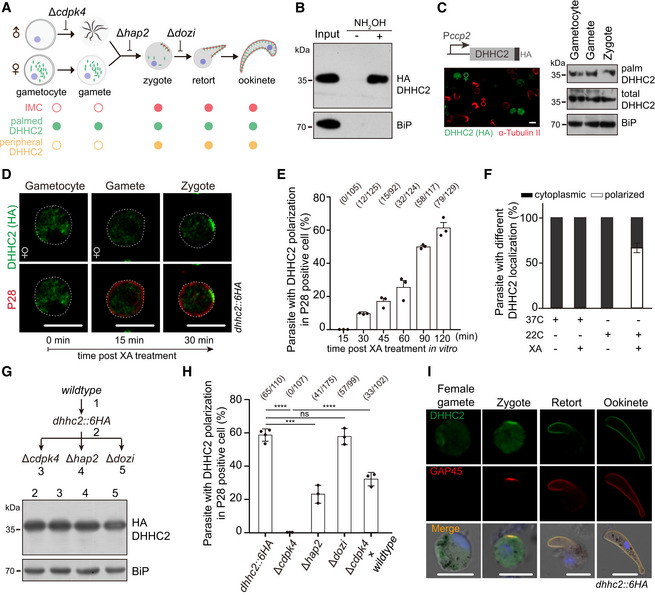
Newly assembled IMC is a polarity patch for DHHC2 targeting in fertilized zygote Schematic of IMC assembly during gametocyte to ookinete. IMC assembly initiates in apical and extends along protrusion of the fertilized zygotes.Endogenous DHHC2 is palmitoylated in the *dhhc2::6HA* gametocytes.Episomally expressed DHHC2 driven by promoter of *ccp2* gene is palmitoylated in female gametocytes, female gametes, and zygotes of WT parasites. Left panel shows the expression of HA‐tagged DHHC2 in female gametocyte by co‐staining with antibodies of HA and *α*‐tubulin II (male gametocyte‐specific). Scale bar = 5 μm.IFA of DHHC2 and P28 in female gametocytes, female gametes, and zygotes of the *dhhc2::6HA* parasite. Gametocytes were stimulated with XA and 22°C *in vitro*. P28 is expressed specifically in female gametes and zygotes. Scale bar = 5 μm.Quantification of parasites with DHHC2 polarization in P28‐positive cells during gametocyte‐to‐zygote differentiation in (D). x/y: the number of cells with DHHC2 polarization and the number of cells analyzed. Values are means ± SD from three independent tests.No polarization of DHHC2 in the *dhhc2::6HA* gametocytes with only either XA or 22°C stimulation. Three replicates are performed, and values are means ± SD.No change in DHHC2 expression in zygotes of the *dhhc2::6HA* parasite‐derived mutants with individual gene disruption of *cdpk4*,* hap2*, and *dozi*.Quantification of parasite with DHHC2 polarization in P28‐positive cells. Parasites are indicated in (G) or from a genetic cross (*dhhc2::6HA/cdpk4* and WT). x/y: the number of cells with DHHC2 polarization and the number of cells analyzed. Values are means ± SD from three tests, two‐tailed *t*‐test, ***P < 0.001, ****P < 0.0001, ns, not significant. Cell images in Appendix Fig S4.Expression and localization of DHHC2 and GAP45 during female gamete‐to‐ookinete development of *dhhc2::6HA* parasite. GAP45 is an IMC marker and is only detected in zygote after fertilization, but not gametes. Scale bar = 5 μm. Source data are available online for this figure.

In mosquito midgut, male and female gametocytes are activated and within 10‐15 min differentiate to male and female gametes that further fertilize to form a zygote (Fig 6A). Consistent with the self‐palmitoylation property of DHHC2, we detected palmitoylation of endogenous DHHC2 in purified *dhhc2::6HA* gametocytes (Fig 6B). This modification also occurred in episomally expressed DHHC2 driven by promoter of the *ccp2* gene (Fig 6C), a gene that is transcribed specifically in female gametocytes, female gametes, and zygote (Liu *et al*, 2018). However, DHHC2 was expressed in the cytoplasm of female gametocytes (P28‐negative) and female gametes (PPM localization of P28) of the *dhhc2::6HA* parasites (Figs 6D and EV3B). These results suggest that the palmitoylated DHHC2 in the cytoplasm of female gametes translocates to the membranes of newly assembled IMC only after fertilization and zygote formation. Consistently, DHHC2 polarization at cell periphery occurred in P28‐positive parasites in a time‐dependent manner, starting at 30 min and peaking at 2 h post‐XA stimulation (Fig 6E). This lag in time correlates with the time required for gamete fertilization and zygote formation after gametogenesis. In addition, no DHHC2 polarization was detected in parasites stimulated with only either XA or 22°C due to the failure of gametogenesis and lack of fertilization (Fig 6F).

We next disrupted *cdpk4* gene, which is essential for male gamete formation (Billker *et al*, 2004), and *hap2* gene, which is critical in female and male gametes fusion (Liu *et al*, 2008), in the *dhhc2::6HA* parasite. Depletion of CDPK4 or HAP2 had no effect on DHHC2 protein abundance (Fig 6G), but caused complete or severe loss of DHHC2 polarization in P28‐positive parasites post‐XA treatment (Fig 6H, Appendix Fig S4). As a control, deletion of the *dozi* gene, which is essential for ookinete differentiation post‐zygote formation (Mair *et al*, 2010), had no effect on DHHC2 polarization (Fig 6H). We crossed the DHHC2 polarization‐deficient strain *dhhc2::6HA/∆cdpk4* with WT parasite, and observed DHHC2 polarization at zygotes, presumably from fertilization between female gamete of *dhhc2::6HA/∆cdpk4* and male gamete of WT parasite (Fig 6H). Lastly, we double‐stained the *dhhc2::6HA* parasite with antibodies against HA and the IMC‐residing protein GAP45 (Frenal *et al*, 2010). DHHC2 was expressed in cytoplasm of female gametes where GAP45 was not detected (Fig 6I), in agreement with previous observations that there is no IMC present in female gamete of *P. berghei* (Mons, 1986). After fertilization, DHHC2 translocated to IMC and showed IMC localization as GAP45 during the whole zygote–retort–ookinete differentiation (Fig 6I). These results suggest that the newly assembled IMC in fertilized zygote provides a polarity patch to recruit the cytoplasmic palmitoylated DHHC2 (Fig 6A).

### ISP1/ISP3 maintains the dome‐like SPM structure in zygote elongation

**Figure EV5:**
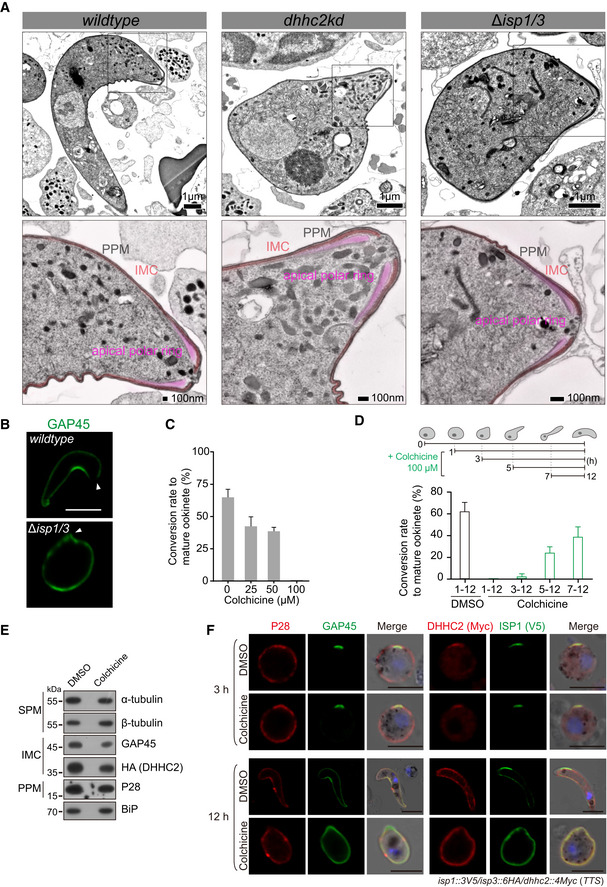
ISP1 and ISP3 have no effect on IMC formation and apical protrusion TEM longitudinal sections of WT, *dhhc2kd,* and *∆isp1/3* parasites collected from 12‐h *in vitro* ookinete culture. The IMC is formed underneath the PPM (parasite plasma membrane) in both WT and mutant parasites. Inserts in upper images are magnified to indicate the IMC and apical protrusion. PPM, black; IMC, pink; and apical polar ring, purplish red.Expression and localization of GAP45 in WT and *∆isp1/3* ookinetes from 12‐hour *in vitro* culture. Arrowheads point to cell apical. Scale bar = 5 μm.
*In vitro* ookinete differentiation of WT parasite treated with different concentrations of colchicine, an inhibitor of microtubule polymerization in *Plasmodium*. The data show quantification from two independent repeats, and values are means ± SD.Inhibiting effect of colchicine on ookinete differentiation is time‐dependent. Colchicine treatment of parasite during early development (1–3 h) blocks ookinete differentiation. The data show quantification from three independent repeats, and values are means ± SD.Western blot of α‐tubulin, β‐tubulin, GAP45, and DHHC2 expression in the *dhhc2::6HA* zygote culture treated with colchicine.IFA of P28, GAP45, ISP1, and DHHC2 expression at early (3 h) or later (12 h) ookinete of the colchicine‐treated *TTS* parasites. Scale bar = 5 μm. Source data are available online for this figure.

How does ISP1/ISP3 regulate the process of protrusion–elongation–maturation during zygote‐to‐ookinete transition? We examined the IMC and SPM structures in ookinetes of the *dhhc2kd* and *∆isp1/3* parasites. Transmission electron microscope (TEM) revealed intact IMC underlying PPM in both *dhhc2kd‐* and *∆isp1/3‐*defective ookinetes from 12‐hour *in vitro* culture, which is indistinguishable from that of WT (Fig EV5A). TEM also showed intact apical protrusion formed in defective ookinetes of both mutant parasites (Fig EV5A). Interestingly, IMC distributed along the whole‐cell periphery in either dhhc2kd‐ or *∆isp1/3‐*defective ookinetes (Fig EV5A), which is further confirmed by GAP45 staining in the *∆isp1/3* ookinetes (Fig EV5B). These results indicate that ISP1/ISP3 depletion has no effect on IMC assembly and extension. In addition, the defect may not occur in apical protrusion of zygote, but in the elongation after protrusion.

**Figure 7:**
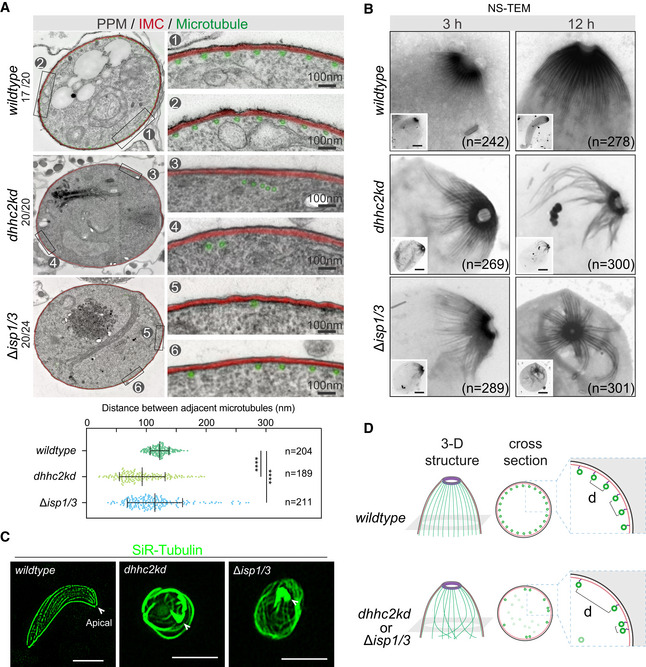
ISP1 and ISP3 maintain the dome‐like SPM structure Transmission electron microscopy (TEM) cross sections of WT, *dhhc2kd,* and *∆isp1/3* parasites showing the arrangement of SPM underneath IMC. Approximately 60 recognizable hollow microtubules (green) are associated with IMC (red), distributing evenly around circumference with an interval of 100–120 nm in WT ookinetes. Microtubules lost association with IMC in the ookinetes of *dhhc2kd* and *∆isp1/3* parasites. Left: One representative cell in each parasite is shown; right: Two areas are magnified to show the details. Lower panel is the quantification of distance between adjacent microtubules associated with IMC. Values are mean ± SD for *n* = 204 (WT), 189 (*dhhc2kd*), and 211 (*∆isp1/3*) measurements in 20 cells each group from two independent experiments; Kolmogorov–Smirnov test, ****P < 0.0001.Apical polar ring and the emanating SPM in WT, *dhhc2kd,* and *∆isp1/3* parasites examined by negative staining and TEM. A dome‐like SPM structure radiating from the apical polar ring was observed in early (3 h) or mature (12 h) WT ookinetes, but impaired in mutant parasites. n is the number of cells analyzed from three independent experiments. More images in Appendix Fig S5. Scale bar = 1 μm.SiR‐tubulin staining shows the SPM structures of WT, *dhhc2kd,* and *∆isp1/3* parasites. Arrowheads point to cell apical. Scale bar = 5 μm.Proposed role of DHHC2/ISP1/ISP3 in maintaining the dome‐like SPM structure consisting of 60 microtubules in ookinete elongation. Microtubules lost association with IMC in the ookinetes of either *dhhc2kd* or *∆isp1/3* parasites.

Detailed TEM images from cell transversal sections showed hollow microtubules closely associated with IMC in ookinetes (Fig 7A). Approximately 60 recognizable microtubules were distributed evenly around the circumference with an interval of 120 ± 15 nm (Fig 7A) in WT ookinetes, similar to those of *P. cynomolgi* and *P. falciparum* (Garnham *et al*, 1962; Bounkeua *et al*, 2010). In contrast, the microtubules associated with IMC were remarkably decreased in mutant ookinetes, with 5 ± 3 microtubules in *∆isp1/3* (88%, 20/24) and none in *dhhc2kd* (100%, 20/20) compared with 60 ± 2 in WT (85%, 17/20) (Fig 7A), suggesting microtubule detachment from IMC or no attachment to IMC in a first place. We measured the distance between adjacent IMC‐associating microtubules and found the mean distance between microtubules was 90 ± 45 nm in *dhhc2kd* and 110 ± 55 nm in *∆isp1/3* parasite (Fig 7A, lower panel). After depletion of either DHHC2 or ISP1/ISP3, the spacing of microtubules changed dramatically with smaller and larger gaps appearing more frequently. To see more details of the defects, the parasites from 3‐ and 12‐h *in vitro* culture were detergent‐extracted and examined under TEM after negative staining (NS‐TEM), a method used to observe microtubule cytoskeleton of the *T. gondii* tachyzoite (Leung *et al*, 2017; Long *et al*, 2017). Consistently, the microtubules in both early and mature WT ookinetes were arranged in a dome‐like pattern regularly radiating from the apical polar ring (Fig 7B). This is an observation of apical polar ring as MTOC linking the SPM in *Plasmodium* ookinetes. In contrast, the microtubules were clumped or twined in both *dhhc2kd* and *∆isp1/3* parasites, with the presence of intact apical polar ring (Fig 7B). Furthermore, we stained the parasites with SiR‐tubulin, a Taxol derivative that fluorescently labels microtubules in the *P. berghei* sporozoite (Spreng *et al*, 2019). The microtubules were regularly emanated from the anterior apex to the posterior end, fitting the crescent shape of WT ookinetes. However, this pattern of microtubules was absent in the elongation‐defective ookinetes of both mutant parasites (Fig 7C), suggesting the roles of DHHC2 and ISP1/ISP3 not in microtubule assembly or extension, but in structure pattern of SPM. Together, these results show that IMC‐anchored ISP1 and ISP3 function to maintain the dome‐like structure of SPM for ookinete elongation after initial apical protrusion (Fig 7D).

To test the role of intact SPM in ookinete elongation, we treated the *TTS* zygote culture with colchicine, an inhibitor of microtubule polymerization in *Plasmodium* (Kumar *et al*, 1985), and observed a dosage‐dependent inhibition of ookinete differentiation (Fig EV5C) as previously reported. Complete inhibition of mature ookinete formation was achieved at 100 μΜ colchicine (Fig EV5C), resembling the defects of the *dhhc2kd* and *∆isp1/3* mutants. The inhibitory effect of colchicine is restricted to the initial 3–4 h during differentiation (Fig EV5D), which is similar to the effect of 2‐BP treatment (Fig EV4I). Colchicine did not affect protein abundance of α‐tubulin, β‐tubulin, GAP45, and DHHC2 in the *TTS* zygote culture 12 h after treatment (Fig EV5E). In addition, colchicine treatment had no effect on apical polarization of GAP45, DHHC2, and ISP1 in 3‐h early‐stage ookinetes (Fig EV5F) or whole‐cell periphery distribution of these IMC proteins in 12‐h elongation‐defective ookinetes (Fig EV5F), further suggesting that IMC assembly and extension underneath the PPM are SPM‐independent.

### ISP1 likely interacts with β‐tubulin

**Figure 8:**
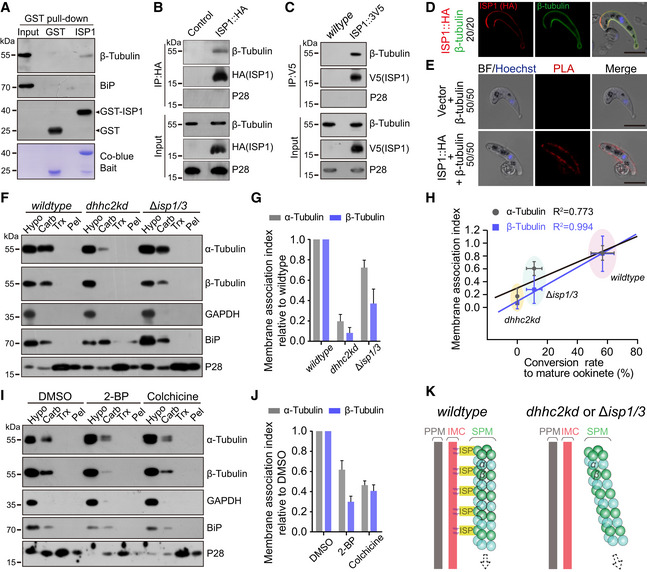
ISP1 likely interacts with β‐tubulin in ookinete GST‐ISP1 pulls down endogenous β‐tubulin from cell lysate of WT ookinetes. Lower panel indicates the Coomassie blue stain of recombinant GST and GST‐ISP1.Episomally expressed ISP1::HA immunoprecipitated with β‐tubulin in WT ookinetes. Episomal vector as control.Endogenous ISP1::3V5 immunoprecipitated with β‐tubulin in the *TTS* ookinetes.Co‐localization of ISP1::HA and β‐tubulin at ookinete periphery in IFA. Scale bar = 5 μm.Proximity ligation assay (PLA) detecting interaction of ISP1‐HA and β‐tubulin at ookinete. Episomal vector as control. Scale bar = 5 μm.Solubility assay detecting membrane association of α‐ and β‐tubulins in WT, *dhhc2kd*, and *∆isp1/3* parasites. GAPDH is a cytosolic soluble protein, and P28 is a plasma membrane integral protein.Quantification of α‐ and β‐tubulins membrane association in F. The membrane association index is defined as the ratio of Carb fraction to Hypo fraction in F and normalized to that of WT. Values are means ± SEM from three independent tests.Linear correlation of membrane association index of α‐tubulin and β‐tubulin with the conversion rate to mature ookinete among WT, *dhhc2kd,* and *∆isp1/3* parasites. Horizontal and vertical values are means ± SD.Membrane association of α‐ and β‐tubulins in WT ookinete culture after DMSO, 2‐BP, or colchicine treatment.Quantification of α‐ and β‐tubulins membrane association in I. Values are means ± SEM from three independent repeats.Model showing ISP1/ISP3 interaction with β‐tubulin for anchoring microtubules with IMC in the elongating ookinetes. Source data are available online for this figure.

The role of ISP1 and ISP3 in maintaining SPM structure prompted us to test potential interaction between ISP proteins and microtubule in developing ookinetes. Tubulins are the main component of microtubules that are composed of α‐ and β‐tubulin heterodimers in eukaryotic cells. β‐Tubulin has been shown to bind each of the two pleckstrin homology (PH) domains of human phospholipase C‐γ1 (PLC‐γ1) (Chang *et al*, 2005). Interestingly, both *T. gondii* ISP1 and ISP3 possess a PH domain (Tonkin *et al*, 2014), which is structurally conserved in both PyISP1 and PyISP3 (Fig EV1G), suggesting potential interaction between PyISP PH domains and β‐tubulin. We expressed and purified a recombinant GST‐ISP1 fusion protein in *E. coli* and the GST‐ISP1 successfully pulled down β‐tubulin from cell extract of the WT ookinetes (Fig 8A), suggesting that ISP1 interacts with β‐tubulin. This interaction was further supported by additional evidences. First, co‐immunoprecipitation of episomally expressed ISP1::HA and endogenous β‐tubulin using anti‐HA antibody in WT ookinetes (Fig 8B) or pull‐down of endogenous β‐tubulin using anti‐V5 antibody in ookinetes of the *isp1::3V5* parasite (Fig 8C) further revealed that ISP1 binds β‐tubulin. Second, two‐color IFA further showed that ISP1::HA co‐localizes with β‐tubulin at the ookinete periphery (Fig 8D). Third, we performed the proximity ligation assay (PLA) to test the interaction between ISP1 and β‐tubulin. Consistent with the IFA results, PLA signals were observed at the ookinete periphery (Fig 8E), suggesting a strong association between ISP1 and β‐tubulin. These data demonstrate that IMC‐anchored ISP1 interacts with SPM component β‐tubulin around cell periphery of whole ookinete, explaining the role of ISP protein in maintaining proper structure of SPM. Consistent with these observations, the membrane associations of α‐ and β‐tubulin were greatly reduced in DHHC2 knockdown or ISP1/ISP3‐depleted parasites, compared with those of WT parasites in protein solubility assay (Fig 8F and G). Moreover, there was a positive correlation between mature ookinete conversion rate and membrane association level of α‐tubulin (*R* ^2^ = 0.773) or β‐tubulin (*R* ^2^ = 0.994) among the WT, *∆isp1/3,* and *dhhc2kd* parasites (Fig 8H), suggesting a structural role of ISP1/β‐tubulin interaction in ookinete elongation. Consistently, decreased membrane association of α‐ and β‐tubulin was also observed in the WT zygote culture after 12‐hour treatment with either 2‐BP or colchicine (Fig 8I and J). Taken together, these results suggest that IMC‐anchored ISP proteins may interact with or get close proximity to β‐tubulins of microtubules to maintain structural integrity of SPM in the elongating ookinetes (Fig 8K).

## Discussion

**Figure 9:**
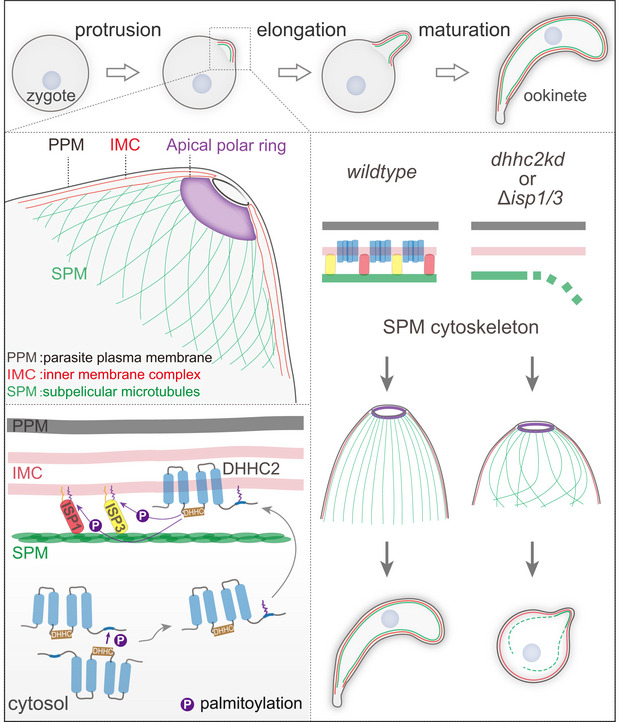
Working model of DHHC2/ISP1/ISP3 maintaining SPM structure during zygote‐to‐ookinete differentiation PPM: parasite plasma membrane, IMC: inner membrane complex, and SPM: subpellicular microtubules.

The *Plasmodium* round‐shaped zygotes undergo a remarkable cell morphogenesis, including protrusion–elongation–maturation, to form crescent‐shaped ookinetes that glide and traverse mosquito midgut for transmission. During this morphogenesis, the apical polar ring acts as a MTOC to nucleate microtubules (Morrissette & Sibley, 2002). Approximately 60 microtubules with a regular interval distance emanate from the apical polar ring toward posterior in a radial arrangement (Garnham *et al*, 1962; Bounkeua *et al*, 2010), establishing a dome‐like structure of SPM underneath IMC as cytoskeleton essential for ookinete morphogenesis and gliding movement. How the parasites establish and maintain this dome‐like structure of SPM underneath IMC is not clear. In this study, we reveal that four pellicle proteins, including DHHC2, ISP1, and ISP3 in IMC and β‐tubulin in SPM, play pivotal roles in maintaining the proper structure of SPM during zygote‐to‐ookinete differentiation; see the working model in Fig 9. This dome‐like structure of SPM is anchored and stabilized by IMC via ISP1/ISP3‐β‐tubulin tethering, providing structural support for zygote elongation after protrusion. Microtubules detached from IMC fail to form this dome‐like array as significantly reduced microtubule–IMC attachment or association was observed in the elongation‐defective ookinetes of *dhhc2kd* or *∆isp1/3* parasites.

The IMC is composed of a patchwork of flattened membrane vesicles (Morrissette & Sibley, 2002; Agop‐Nersesian *et al*, 2010) and provides a central scaffold for maintaining the polarity and morphology of apicomplexan zoites, presumably by coordinately connecting PPM and SPM via different bridging molecules. GAP45, an IMC‐residing protein, plays a role in connecting IMC and PPM (Frenal *et al*, 2010). Cryogenic electron tomography revealed that the microtubules are linked to the parasite pellicle via the IMC‐originated long tethering proteins in the *Plasmodium* sporozoites (Kudryashev *et al*, 2010). The longitudinal rows of intramembrane particles have been also observed between IMC and SPM in *T. gondii* (Morrissette *et al*, 1997), representing possible bridging molecules connecting IMC and SPM. A recent study also revealed an IMC integral membrane protein GAPM1a that functions to maintain the stability of the underneath microtubule in tachyzoites of *T. gondii* (Harding *et al*, 2019). Depletion of GAPM1a caused disorganization and depolymerization of cortical microtubule and resulted in defective morphology of tachyzoites, mimicking the structural defects of the *dhhc2kd* and *∆isp1/3* parasites in this study. Therefore, the IMC‐anchored ISP1 and ISP3 may act as a tether linking SPM via interaction with microtubule component β‐tubulin to maintain proper pellicle cytoskeleton and to support cell elongation during zygote‐to‐ookinete differentiation. Although most parasites arrest in early stages of differentiation, approximately 10–15% convert to mature ookinete after depletion of both ISP1/ISP3, suggesting other bridging molecules also contribute to the connection of IMC and SPM. The *P. berghei* parasites with disruption of PDEδ, which degrades cGMP, first develop to fully formed crescent‐shaped ookinetes but further shrink to the round or stumpy form (Moon *et al*, 2009), likely a developmental degeneration. Compared to the PDEδ mutant, this degeneration was not observed in either ∆*isp1/3* or *dhhc2kd* parasites, suggesting a structural rather than developmental defect caused by deficiency of these IMC molecules.

Palmitoylation is a lipid modification of proteins that directs protein to specific cellular membranes. Recently, the importance of protein palmitoylation in *Plasmodium* has been well recognized, including parasite development, pathogenesis, and transmission (Jones *et al*, 2012; Tremp *et al*, 2014, 2017; Hodson *et al*, 2015; Santos *et al*, 2015; Hopp *et al*, 2016; Brown *et al*, 2017). Our study demonstrates that the palmitoylation of N‐terminal cysteines directs the IMC localization of ISP1/ISP3 proteins which is crucial to the function of ISP1/ISP3 during zygote‐to‐ookinete differentiation. Furthermore, we identified DHHC2 as the PAT enzyme that localizes at IMC and palmitoylates ISP1/ISP3 during this differentiation. The DHHC2‐ISP1/ISP3 represents the first PAT–substrate pair discovered in *Plasmodium* parasites.

Palmitoyl‐S‐acyl‐transferase enzymes are localized in ER, Golgi, and plasma membrane of many organisms as well as in parasite‐specific organelles, such as IMC and rhoptry (Hodson *et al*, 2015; Daniotti *et al*, 2017). However, it remains unclear how different PATs are targeted to endomembrane compartments. DHHC2 can undergo self‐palmitoylation at its C‐terminal cysteines, which is critical for DHHC2 localization at IMC during zygote‐to‐ookinete differentiation. Strikingly, the self‐palmitoylation of DHHC2 is catalyzed by its own PAT activity, likely through intermolecular interaction of DHHC2. To the best of our knowledge, *Plasmodium* DHHC2 is the first PAT enzyme identified so far with self‐palmitoylation feature. The self‐palmitoylation is not only essential for the proper localization of DHHC2 itself, but also critical for the palmitoylation and function of the downstream substrates, ISP1 and ISP3.

DHHC2 is expressed in the cytoplasm (likely membrane‐bound vesicles) of both female gametocytes and female gametes of *P. yoelii* where no IMC forms but DHHC2 is palmitoylated, in agreement with the nature of self‐palmitoylation of DHHC2. In addition, these results indicate that DHHC2 palmitoylation exists before fertilization and zygote formation. What is the signal that triggers the polarized localization of DHHC2 in zygotes? Upon gamete fertilization to zygote, the IMC starts to assemble at a site underneath PPM and form an apical polarity patch which is able to recruit the palmitoylated DHHC2 from the cytoplasm (Fig 6I). Besides in zygotes, we also observed IMC‐like localization of DHHC2 in mature schizonts where dozen of merozoites form with intact IMC (Fig EV3E). Therefore, the assembly and formation of the apical IMC after fertilization is a trigger for the polarized localization of DHHC2 in zygotes. The specific recruitment of DHHC2 to IMC in both schizont and ookinete implies that the IMC possesses unique membrane composition or component different from that of PPM or other organelle membranes in parasite. Lipid raft, a cholesterol‐rich membrane structures, plays a role in membrane targeting of palmitoylated proteins (Yang *et al*, 2010). However, the nature of this membrane subdomains and its affinity for palmitoylated proteins is still not clear (Blaskovic *et al*, 2013; Chamberlain *et al*, 2013). To date, no study of IMC component in *Plasmodium* has been reported, likely due to technical difficulty in purifying IMC. More future work is required to fully elucidate the mechanisms by which the IMC recruits the palmitoylated DHHC2.

## Materials and Methods

### Animal use and ethics statement

All animal experiments were performed in accordance with approved protocols (XMULAC20140004) by the Committee for Care and Use of Laboratory Animals of Xiamen University. The ICR mice (female, 5 to 6 weeks old) were purchased from the Animal Care Center of Xiamen University and used for parasite propagation, drug selection, parasite cloning, and mosquito feedings. An *Anopheles stephensi* mosquito colony (strain Hor) was reared at 28°C, 80% relative humidity, and at a 12‐h light/dark cycle in the standard insect facility. Adult mosquitoes were maintained on a 10% sucrose solution.

### Plasmid construction and parasite transfection

CRISPR/Cas9 plasmid pYCm was used for parasite genomic modification. To construct plasmid vectors for gene editing, we amplified 5′ and 3′ genomic sequence (400–500 bp) of target genes as homologous arms using specific primers (Appendix Table S2) and inserted the sequences into specific restriction sites in pYCm. The sgRNAs were designed to target the coding region of a gene (Appendix Table S2) using the online program EuPaGDT. Oligonucleotides for guide RNAs (sgRNAs) were mixed in pairs, denatured at 95°C for 3 min, annealed at room temperature for 5 min, and ligated into pYCm. DNA fragments encoding 6HA, 4Myc, 3V5, and Flag tags or BFP were inserted between the left and right arms in frame with gene of interest. For each gene, two sgRNAs were designed to target sites close to the C‐ or N‐terminal part of the coding region. Infected red blood cells (iRBCs) were electroporated with 5–10 μg plasmid DNA using Lonza Nucleofector described previously (Zhang *et al*, 2014). Transfected parasites were immediately intravenously injected into a naïve mouse and were exposed to pyrimethamine (7 mg/ml) 24 h post‐transfection. Parasites with transfected plasmids usually appear after 5–6 days under drug pressure. Some modified parasites subjected for sequential modification were negatively selected to remove pYCm plasmid. 5‐Fluorouracil (5‐FC, Sigma, F6627) was prepared in water (2.0 mg/ml) and provided to the mice in drinking water in a dark bottle. A naive mouse receiving parasites with residual plasmid after pyrimethamine selection was subjected to 5‐FC pressure until PCR confirmed no residual plasmid. The primers used are provided in Appendix Table S2.

### Genotypic analysis of transgenic parasites

All transgenic parasites were generated from *P. yoelii* 17XNL strain and are listed in Appendix Table S1. Blood samples from infected mice were collected from the mouse tail tip and lysed using 1% saponin in PBS. Parasite genomic DNAs were isolated from the blood samples using DNeasy Blood Kits (Qiagen) after washing off hemoglobin. For each genetic modification, both 5′ homologous recombination and 3′ homologous recombination were detected by diagnostic PCR (Appendix Fig S1) to confirm successful integration of the homologous templates. All the primers used for genotyping are provided in Appendix Table S2. Positive clones with targeted modifications were obtained after limiting dilution. At least two clones for each genetically modified parasite were used for phenotype analysis.

### Gametocyte induction in mouse

ICR mice were treated with phenylhydrazine (80 mg/g mouse body weight) through intraperitoneal injection. Three days post‐injection, the mice were infected with 4.0 × 10^6^ parasites through intravenous injection. Gametocytemia usually peaks at day 3 post‐infection. Male and female gametocytes were counted after Giemsa staining of blood smears. Sex‐specific gametocytemia was calculated as the ratio of male or female gametocytes over iRBCs. All experiments were repeated three times independently.

### *In vitro* ookinete differentiation and characterization

*In vitro* ookinete differentiation was performed as described previously (Gao *et al*, 2018). Briefly, 1 ml mouse blood with 8–10% gametocytemia was collected via orbital sinus and immediately transferred to a 10‐cm cell culture dish (Corning, cat# 801002) containing ookinete culture medium (RPMI 1640, 25 mM HEPES, 10% FCS, 100 mM xanthurenic acid, pH 8.0) to allow gametogenesis, fertilization, and ookinete development at 22°C. For ookinete conversion analysis, samples from 12 h of culture were collected and stained by Giemsa solution (Sigma, cat# GS80). The conversion rate to retort and ookinete was calculated as the number of ookinetes (stages I–V) per 100 female gametocytes. The conversion rate to mature ookinete was calculated as the number of mature ookinetes (stage V) over that of total ookinetes (stages I–V). For time‐course analysis of ookinete differentiation, samples from 2, 4, 6, 8, and 12 h of culture were collected, and ookinetes in stages I and II were counted. Ookinetes were purified using ACK lysing method as described previously (Gao *et al*, 2018). Briefly, erythrocytes were lysed using the ACK lysis buffer (Thermo Fisher Scientific, A1049201), and ookinetes were counted under the hemocytometer. Purified ookinetes were used for further biochemical analysis.

### Chemical inhibition of zygote‐to‐ookinete differentiation

To evaluate the effects of protein palmitoylation inhibitor 2‐BP (Sigma‐Aldrich, cat# 21604) on ookinete differentiation, 2‐BP was added to *in vitro* ookinete cultures with final concentration of 100 μM 1 h after fertilization. To evaluate the effects of microtubule assembly inhibitor colchicine (MedChemExpress, cat# HY‐16569) on ookinete differentiation, colchicine was added to cultures with final concentration of 100 μM 1 h after fertilization. Ookinete cultures were harvested at different times for further analysis.

### Mosquito feeding and transmission assay

For mosquito transmission, thirty *Anopheles stephensi* mosquitoes were allowed to feed on an anesthetized mouse carrying 8–10% gametocytemia for 30 min at 22°C. Mosquito midguts were dissected at day 7 post‐blood feeding, and oocysts were stained with 0.5% mercurochrome. Twenty mosquito salivary glands were dissected to count sporozoites at day 14.

### Episomal protein expression

Coding sequence of target proteins with appropriate 5′ UTR and 3′ UTR regulatory regions were inserted into the pL0019‐derived vector with human *dhfr* for pyrimethamine selection (Gao *et al*, 2018). Firstly, schizonts were electroporated with 10 μg plasmid DNA. Transfected parasites were immediately intravenously injected into a naïve mouse and were exposed to pyrimethamine (70 μg/ml) for 5–8 days. Parasites appeared after pyrimethamine selection were transferred to a phenylhydrazine‐treated naïve mice by injecting 4.0 × 10^6^ iRBCs intravenously to induce gametocytes and were kept under pyrimethamine pressure. The mice with high gametocytemia were used for further study.

### Antibodies and antiserum

The primary antibodies used were as follows: rabbit anti‐HA (Cell Signaling Technology, cat# 3724S, 1:1,000 for immunoblotting (IB), 1:500 for immunofluorescence (IF), 1:500 for immunoprecipitation (IP)). Mouse anti‐HA (Cell Signaling Technology, cat# 2367S, 1:500 for IF), rabbit anti‐Myc (Cell Signaling Technology, cat# 2272S, 1:1,000 for IB, 1:500 for IP), mouse anti‐Myc (Cell Signaling Technology, cat# 2276S, 1:500 for IF), mouse anti‐α‐tubulin II (Sigma‐Aldrich, cat# T6199, 1:1,000 for IF, 1:1,000 for IB), mouse anti‐β‐tubulin (Sigma‐Aldrich, cat# T5201, 1:1,000 for IF, 1:1,000 for IB), mouse anti‐V5 (GeneScript, cat# A01724‐100, 1:500 for IF, 1:1,000 for IB, 1:500 for IP), rabbit anti‐Flag (Sigma‐Aldrich, cat# F2555, 1:1,000 for IB), mouse anti‐acetylated tubulin (Sigma‐Aldrich, cat# T7451, 1:1,000 for IB, 1:1,000 for IF), and mouse anti‐GAPDH (Servicebio, cat# GB12002, 1:1,000 for IB). The secondary antibodies used were as follows: HRP‐conjugated goat anti‐rabbit IgG (Abcam, cat# ab6721, 1:5,000 for IB), HRP‐conjugated goat anti‐mouse IgG (Abcam, cat# ab6789, 1:5,000 for IB), HRP‐conjugated goat anti‐mouse IgG LCS (IPKine, cat# A25012, 1:5,000 for IB), Alexa 555 goat anti‐rabbit IgG (Thermo Fisher Scientific, cat# A21428, 1:1,000 for IF), Alexa 488 goat anti‐rabbit IgG (Thermo Fisher Scientific, cat# A31566, 1:1,000 for IF), Alexa 555 goat anti‐mouse IgG (Thermo Fisher Scientific, cat# A21422, 1:1,000 for IF), and Alexa 488 goat anti‐mouse IgG (Thermo Fisher Scientific, cat# A11001, 1:1,000 for IF). The anti‐serums included rabbit anti‐GAP45 (our laboratory, 1:1,000 for IFA, 1:1,000 for IB), rabbit anti‐P28 (our laboratory, 1:1,000 for IFA, 1:1,000 for IB), and rabbit anti‐BiP (our laboratory, 1:1,000 for IB).

### Immunofluorescence assay and fluorescence microscopy

Cells were fixed with 1 ml of 4% paraformaldehyde for 15 min and rinsed with 1 ml PBS three times. The cells were then permeabilized with 0.5 ml of 0.1% Triton X‐100 for 10 min, rinsed with 1 ml PBS twice, and incubated with 5% BSA for 1 h. They were incubated with the primary antibodies overnight at 4°C, rinsed with 1 ml PBS three times, and incubated with fluorescent conjugated secondary antibodies for 1 h in the dark. After three washes with 1 ml PBS, they were stained with 0.5 ml of DNA dye Hoechst 33342 for 10 min and mounted on glass slides using mounting medium (90% glycerol). All reagents were diluted in PBS and processed under room temperature. The cells were imaged using identical settings under a Zeiss LSM 780 or LSM 880 confocal microscope. For imaging living cell, live parasites were transferred onto a slide under a confocal microscope with a 63× or 100× oil objective. Confocal microscope images were taken, and representative images were shown.

### DNA content measurement of parasite nuclei

To evaluate the nuclear DNA content of zygotes, parasites from 4 h *in vitro* ookinete differentiation cultures were fixed using 4% paraformaldehyde for 15 min, rinsed twice with 1 ml PBS, followed by incubation with 5% BSA for 1 h. The cells were then incubated with anti‐P28 antibody for 1 h. After rinsing twice with 1 ml PBS, they were incubated with fluorescent conjugated secondary antibodies for 1 h, rinsed twice with 1 ml PBS, and stained with DNA dye Hoechst 33342 for 10 min. The female gametes and fertilized zygotes could be labeled with P28. Images were captured using identical settings under a LSM880 confocal microscope. The fluorescent signal intensity was measured using ZEISS software (https://www.zeiss.com/microscopy/int/products/microscope-software/zen.html).

### Quantitative real‐time PCR

Total RNAs were extracted from about 1.0 × 10^7^ purified ookinetes using the TRIzol reagent (Invitrogen). mRNA was purified with a RNeasy Mini Kit (Qiagen). cDNA was obtained with the TransScript^®^ Two‐Step RT‐PCR SuperMix (TransGen Biotech, cat# AT401‐01) and checked for genomic DNA contaminations via RT‐PCR. Real‐time quantitative PCR was performed using SYBR Green Supermix (Bio‐Rad, cat# 1708882) in the Bio‐Rad iCycler iQ system (Bio‐Rad) with 5 μM of primer concentration. The primers used are listed in Appendix Table S2. All cycling conditions were as follows: 95°C for 20 s followed by 40 cycles of 95°C for 3 s; 60°C for 30 s. The samples were run in triplicate with three biological replicates. The 18S rRNA gene was used as a reference. Relative copy numbers in mutant parasites were calculated by applying the ΔΔ*C*_t_ methodology and normalized to that of wild‐type parasite.

### Protein extraction and immunoblotting

Parasite total proteins from asexual blood stages, gametocytes, zygotes, retorts, and ookinetes were extracted with RIPA lysis buffer (50 mM pH 7.4 Tris, 150 mM NaCl, 1% Triton X‐100, 1% sodium deoxycholate, 0.1% SDS, 1 mM EDTA) containing protease inhibitor cocktail and PMSF. After ultrasonication, the lysates were incubated on ice for 30 min before centrifugation at 12,000 *g* for 10 min at 4°C. The supernatant was then lysed in Laemmli sample buffer, stored at 4°C for immunoblotting. The protein samples were separated in SDS–PAGE and transferred to PVDF membrane that was blocked in TBST buffer with 5% skim milk and then incubated with primary antibodies. After incubation, the membrane was washed three times with TBST and incubated with HRP‐conjugated secondary antibodies. The membrane was washed four times in TBST before enhanced chemiluminescence detection.

### Protein immunoprecipitation

Parasites were lysed in IP buffer A (50 mM HEPES pH 7.5, 150 mM NaCl, 1 mM EDTA, 1 mM EGTA, 1% Triton X‐100, 0.1% sodium deoxycholate) with protease inhibitor cocktail and PMSF. Human 293T cells were lysed in IP buffer B (25 mM pH 7.5 Tris–HCl, 150 mM NaCl, 1 mM EDTA, 1 mM EGTA, 1% Triton X‐100, 10% glycerol) with protease inhibitor cocktail and PMSF. One ml of lysates was incubated with primary antibodies (rabbit anti‐HA or rabbit anti‐Myc) overnight. Protein aggregates were precleared by centrifugation at 20,000 *g* for 10 min, and protein A/G beads (1:50) were added into the lysates and mixed for another 3 h. The beads were washed with IP buffer A or IP buffer B for three times at 4°C and then mixed with an equal volume of 2× SDS sample buffer for immunoblotting.

### Detection of protein palmitoylation

Protein palmitoylation was performed using Acyl‐RAC method described previously (Forrester *et al*, 2011). Parasite cells were lysed in DHHC buffer B (2.5% SDS, 1 mM EDTA, 100 mM HEPES, pH 7.5) containing protease inhibitor cocktail and PMSF and incubated on ice for 30 min. After centrifugation at 12,000 *g* for 10 min, supernatant was collected and treated with 0.1% methyl methanethiosulfonate (MMTS, Sigma‐Aldrich, cat# 2949‐92‐0) at 42°C for 15 min. MMTS was removed by acetone precipitation followed with washing with 70% acetone three times. Protein samples were solubilized in DHHC buffer C (1% SDS, 1 mM EDTA, 100 mM HEPES, pH 7.5) and added with 20 μl Thiopropyl Sepharose 6B beads (GE Healthcare, cat# 17‐0402‐01). Palmitoylated proteins were captured on beads in the presence of 2 M hydroxylamine (Sigma‐Aldrich, cat# 467804) or 2 M NaCl (negative control). Loading controls (input) were collected before addition of Thiopropyl Sepharose 6B beads. After five times of washing with DHHC wash buffer (8 M urea in DHHC buffer C), the captured proteins were eluted from beads using 60 μl DHHC elution buffer (50 mM DTT in DHHC buffer C) and mixed with 15 μl 5× Laemmli sample buffer for further immunoblotting.

### Mammalian cell culture and transient transfection

HEK293T cells were maintained in Dulbecco's modified Eagle's medium (DMEM) supplemented with 10% fetal bovine serum (FBS), 100 IU penicillin, and 100 mg/ml streptomycin at 37°C in a humidified incubator containing 5% CO_2_. TurboFect transfection reagent (Thermo Fisher Scientific, cat# R0532) was used for cell transfection. Total DNA for each plate was adjusted to the same amount by using relevant empty vector. Transfected cells were harvested at 36 h after transfection for further analysis.

### Culture of bacterial *Escherichia coli* strain

*Escherichia coli* strain BL21(DE3) was used and cultured in LB media. After 4 h culture at 37°C, 50 ml culture was added with isopropyl β‐D‐thiogalactopyranoside (IPTG) at a final concentration of 1 mM. The culture was allowed to grow for another 18 h at 22°C. For chemical inhibitor treatment, 2‐BP (Sigma‐Aldrich, cat# 21604) at a final concentration of 100 μM was concurrently added to the culture with the IPTG. All samples were used for protein palmitoylation analysis.

### Electron microscope analysis

Transmission electron microscope (TEM) experiments were performed using the protocol as described previously (Ferguson *et al*, 2005). Briefly, purified parasite was prefixed with 4% glutaraldehyde in 0.1 M phosphate buffer at 4°C overnight, rinsed three times with PBS, post‐fixed with 1% osmium acid for 2 h, and rinsed three times with PBS. The samples were dehydrated with concentration gradient acetone. After embedding and slicing, thin sections were stained with uranyl acetate and lead citrate prior to imaging. For observing parasite cytoskeleton, we used a protocol of negative staining prior to TEM as described previously (Long *et al*, 2017). Fleshly harvested parasites were treated with 0.5 mM deoxycholate at room temperature for 10 min. Detergent insoluble cytoskeleton was pelleted by centrifugation at 800 *g* for 10 min and resolubilized in distilled water. Twenty μl homogeneous suspension was transferred onto the top side of copper (ZXBaiRui, cat# T150), absorbed for 5 min, stained with 1% aqueous phosphotungstic acid (Sigma‐Aldrich, cat# 455970) for 30 s, and then air‐dried. All samples were imaged under the HT‐7800 electron microscope.

### Protein solubility assay

All buffers used for solubility assay contained a protease inhibitor cocktail (MedChemExpress, cat# HY‐K0010). If not otherwise indicated, all steps were carried out on ice. Approximately 1 × 10^6^ purified parasites were prepared for solubility assay as described with minor optimization (Cabrera *et al*, 2012). Different fractions were sequentially extracted by distinct buffers. Briefly, parasites were lysed in 200 μl of hypotonic buffer (10 mM HEPES, 10 mM KCl, pH 7.4), frozen, and thawed twice (−80 to 37°C). The lysates were centrifuged at 20,000 *g* for 5 min at 4°C, and the supernatants containing cytosolic soluble proteins (Hypo) were collected. The pellet after hypotonic lysis was rinsed with 1 ml of ice‐cold PBS, suspended in 200 μl of freshly prepared carbonate buffer (0.1 M Na_2_CO_3_ in deionized water), kept on ice for 30 min, and then centrifuged at 20,000 *g* for 5 min at 4°C. The supernatants containing peripheral membrane proteins (Carb) were collected. The pellet after carbonate extraction was rinsed with 1 ml of ice‐cold PBS, suspended in 200 μl of freshly prepared Triton X‐100 buffer (1% Triton X‐100 in deionized water), kept on ice for another 30 min, and centrifuged at 20,000 *g* for 5 min at 4°C. The supernatants containing integral membrane proteins (Trx) were collected. The final pellet (P) including insoluble proteins and non‐protein materials was resolubilized in 1× Laemmli sample buffer. Equal volume of 2× Laemmli sample buffer was added into the Hypo/Carb/Trx fractions, respectively. All samples were boiled at 95°C for 10 min and centrifuged at 12,000 *g* for 5 min. Equal volume of supernatants from each sample was used for immunoblotting.

### GST pull‐down assay

Plasmids expressing GST‐tagged protein or GST control were transformed and amplified in BL21(DE3) *E. coli* strain, and protein expression was induced by 1 mM IPTG. GST‐tagged proteins (as bait) were purified using glutathione agarose (Thermo Fisher Scientific, cat# 16100). Ookinete cell lysates were incubated with the beads containing GST‐tagged proteins to bind the pray proteins in pull‐down buffer (20 mM Tris, 1 mM EDTA, 1% Triton X‐100, protease inhibitors) for 12 h at 4°C. Beads were rinsed three times in 1 ml wash buffer (20 mM Tris, 1 mM EDTA, 0.2% Triton X‐100, protease inhibitors) and then added with 50 μl 1× Laemmli sample buffer for immunoblotting.

### Proximity Ligation Assay (PLA)

The PLA assay detecting *in situ* protein interaction was performed using the kit (Sigma‐Aldrich: DUO92008, DUO92001, DUO92005, and DUO82049). Ookinetes were fixed with 4% PFA for 30 min, permeabilized with 0.1% Triton X‐100 for 10 min, and blocked with a blocking solution overnight at 4°C. The primary antibodies were diluted in the Duolink Antibody Diluent, added to the cells, and then incubated in a humidity chamber overnight at 4°C. The primary antibodies were removed, and the slides were washed with wash buffer A twice. The PLUS and MINUS PLA probe were diluted in Duolink Antibody Diluent, added to the cells, and incubated in a preheated humidity chamber for 1 h at 37°C. Next, cells were washed with wash buffer A and incubated with the ligation solution for 30 min at 37°C. Then, cells were washed with wash buffer A twice and incubated with the amplification solution for 100 min at 37°C in the dark. Cells were washed with 1X wash buffer B twice and 0.01X wash buffer B once. Finally, cells were incubated with Hoechst 33342 and washed with PBS. Images were captured and processed using identical settings on a Zeiss LSM 780 confocal microscope.

### Bioinformatic method and tool

The genomic sequences of target genes were downloaded from PlasmoDB database (http://plasmodb.org/plasmo/; Aurrecoechea *et al*, 2009). The sgRNAs of a target gene were designed using EuPaGDT (http://grna.ctegd.uga.edu/; Aurrecoechea *et al*, 2017). The transmembrane domains of proteins were identified using the Protter Server (http://wlab.ethz.ch/protter/start/; Omasits *et al*, 2014). Amino acid sequence alignment was performed using MEGA5.0 (Stecher *et al*, 2014). The palmitoylation sites in protein were predicted using CSS‐Palm 4.0 (http://csspalm.biocuckoo.org/; Ren *et al*, 2008). Protein structure modeling was performed using the SWISS‐MODEL (https://www.swissmodel.expasy.org/; Biasini *et al*, 2014). The codon usage was optimized using JCat (http://www.prodoric.de/JCat; Grote *et al*, 2005). The hydrophobicity of peptides was analyzed using ProtScale (https://web.expasy.org/protscale/; Wilkins *et al*, 1999).

### Quantification and statistical analysis

Protein band intensity on Western blot was quantified using Fiji software (Schindelin *et al*, 2012). Signals of target proteins were normalized with that of control protein. Distance between adjacent microtubules associated with IMC in TEM images was also quantified using Fiji software. In IFA analysis, images were acquired under identical parameters of fluorescence microscopy and the fluorescent signals of proteins were quantified using ZEISS software. Statistical analysis was performed using GraphPad Prism 8.0. Values are shown as mean ± SEM or mean ± SD. Two‐tailed *t*‐test, Mann–Whitney test, and Kolmogorov–Smirnov test were used to compare differences between groups. Statistical significance is shown as *P < 0.05, **P < 0.01, ***P < 0.001, and ****P < 0.0001; ns, not significant. Experimental replication and sample size information was indicated within the figure or legend.

## Author contributions

XW and PQ generated the modified parasites, conducted the phenotype analysis, IFA assay, image analysis, mosquito experiments, and biochemical experiments, LY conducted the TEM experiments, and JY and HC supervised the work. XW, PQ, HC, and JY analyzed the data, and JY wrote the manuscript.

## Conflict of interest

The authors declare that they have no conflict of interest.

## Supporting information



Appendix

Expanded View Figures PDF

Source Data for Expanded View

Review Process File

Source Data for Figure 1

Source Data for Figure 2

Source Data for Figure 3

Source Data for Figure 4

Source Data for Figure 5

Source Data for Figure 6

Source Data for Figure 8
