# Supplementary material for: A protein palmitoylation cascade regulates microtubule cytoskeleton integrity in Plasmodium
Source: EMBO J. 2020 May 12;39(13):e104168. doi: 10.15252/embj.2019104168 (PMC7327484; doi:10.15252/embj.2019104168)
Supplement: Supplementary file 1 — Appendix [file EMBJ-39-e104168-s001.pdf]

Appendix.

A Protein Palmitoylation Cascade Regulates Microtubule Cytoskeleton integrity in *Plasmoidum*

Xu Wang[1, 2], Pengge Qian [1, 2], Huiting Cui [1, 2], Luming Yao [1], Jing Yuan[1, 3, *]

Table of contents

1. Appendix Supplementary Figures 1- 5 and figure legends
2. Appendix Table S1 List of genetically modified parasite strains in this study
3. Appendix Table S2 Primers and oligonucleotides used in this study

Appendix Figure S1

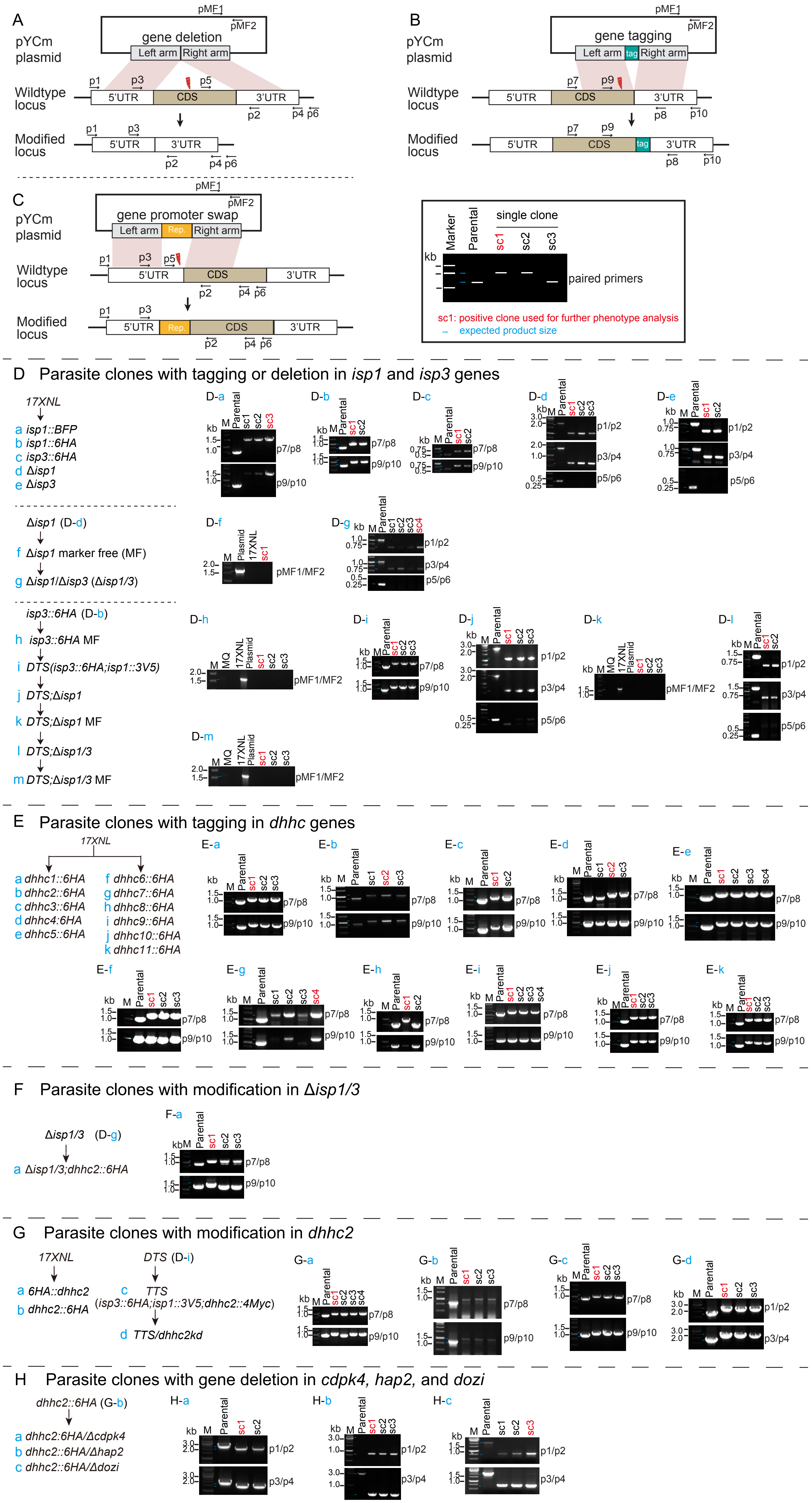

Appendix Figure S1. PCR genotyping of the genetic modified parasite strains in this study.

A-C Schematic for CRISPR/Cas9 mediated gene editing, including gene deletion **(A)**, gene tagging with epitope tag or fluorescence protein **(B)**, and gene promoter swap **(C)** via double cross homologous recombination. Red thunderbolt indicate the DNA site for sgRNA targeting.

D-H In each modification, both the 5' and 3' homologous recombination was detected using gene specific PCR pair (in Appendix Table S2) to confirm correct integration of homologous template. Two single clones (sc) for most mutants and one clone in D-f, E-h, and F-a were obtained with targeted modifications (positive). One positive clone (red letter) was used for phenotype analysis. All genetically modified parasite strains generated in this study are listed in Appendix Table S1. PCR primers sequence and gene ID information are provided in the Appendix Table S2.

Appendix Figure S2

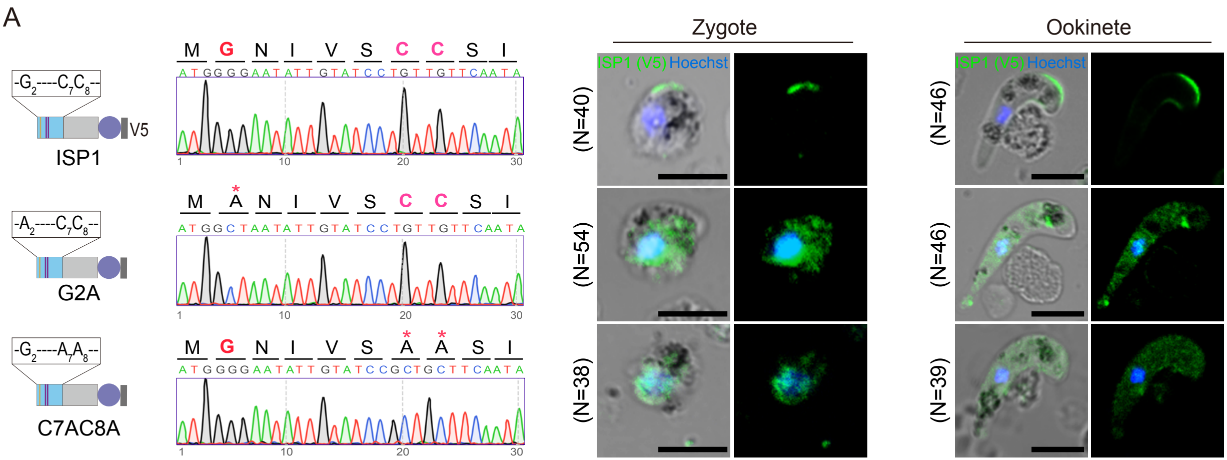

Appendix Figure S2. N-terminal residues of ISP1 are critical for IMC targeting during zygote to ookinete differentiation

Left panel: ISP1 expressing constructs with substitutions of N-terminal cysteine or glycine residues to alanine (G2A or C7A/C8A). ISP1 was fused with BFP::3V5 peptide; middle panel: DNA sequencing confirming substitutions at the targeting residues; right panels: IFA analysis of ISP1 and mutant proteins that episomally expressed in zygotes and ookinetes. Scale bar = 5 μm. N is the number of cells analyzed in each group.

Appendix Figure S3

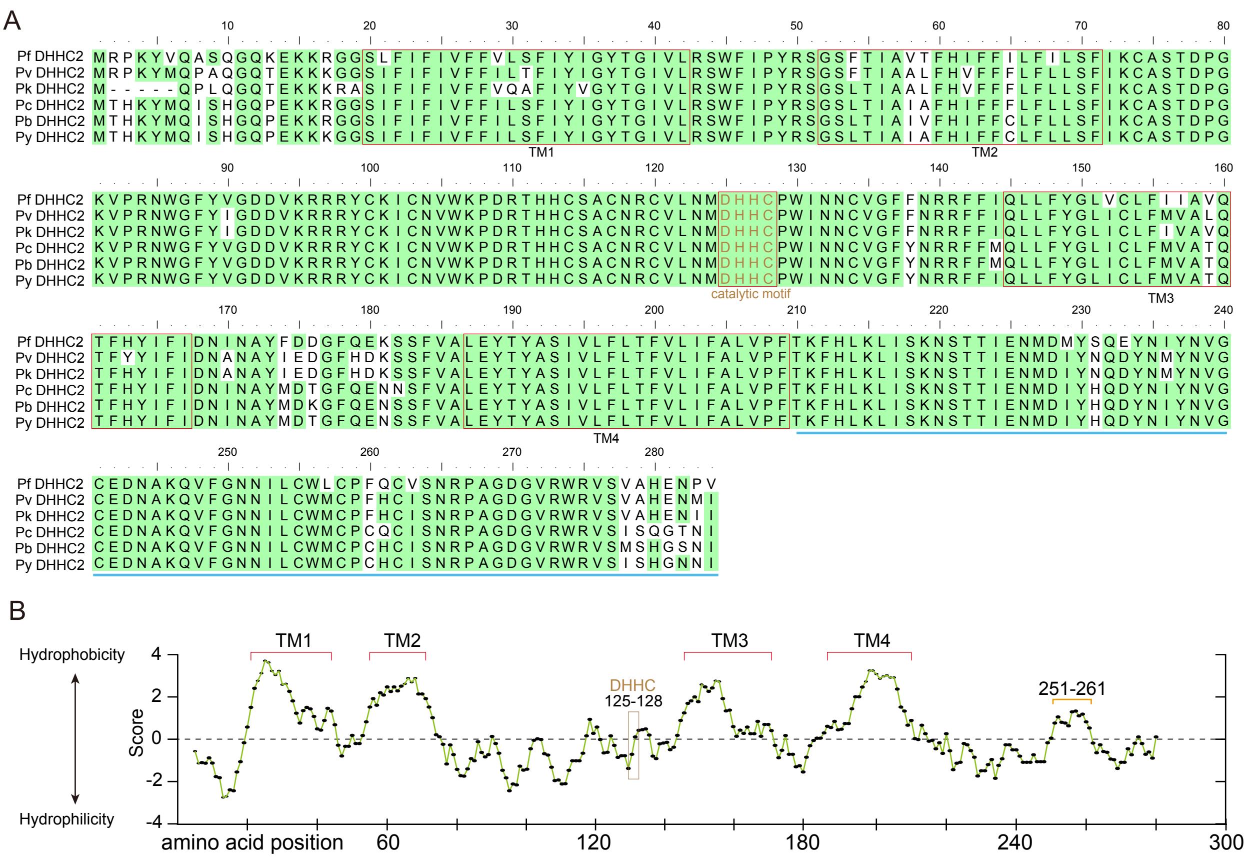

Appendix Figure S3. Protein sequence analysis of *Plasmodium* DHHC2

A Alignment of DHHC2 amino acid sequences from *P. falciparum*, *P. vivax*, *P. knowlesi*, *P. chabaudi*, *P. berghei*, and *P. yoelii* (GeneID: PF3D7_0609800, PVP01_1139200, PKNH_1140400, PCHAS_0108900, PBANKA_0108300, and PY17X_0109900). Four transmembrane domains (TM1-4) and catalytic motif (DHHC) are labeled with red box. C-terminal residues are highlighted with blue line.

B Hydrophilicity and hydrophobicity analysis of DHHC2 along the amino acid sequence. Four TM (TM1-TM4) and a C-terminal segment of residues 251-261 display high hydrophobicity.

Appendix Figure S4

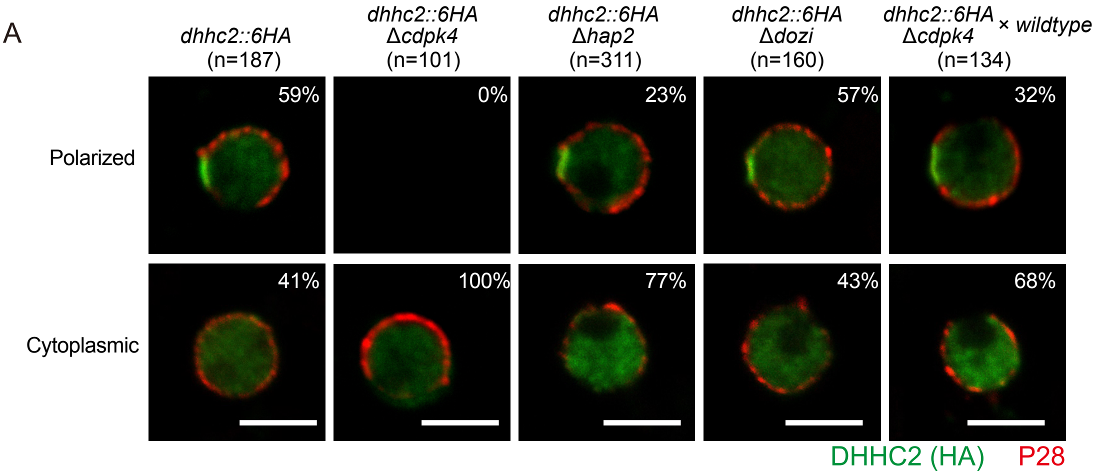

Appendix Figure S4. DHHC2 polarization occurs after gamete fertilization

IFA of DHHC2 and P28 expression in zygotes of the *dhhc2::6HA* derived mutants (including Δ*cdpk4*, Δ*hap2*, and Δ*dozi*), and parasites from a genetic cross between *dhhc2::6HA/*Δ*cdpk4* and WT. n is the number of cells analyzed in each group. Scale bar = 5 μm.

Appendix Figure S5

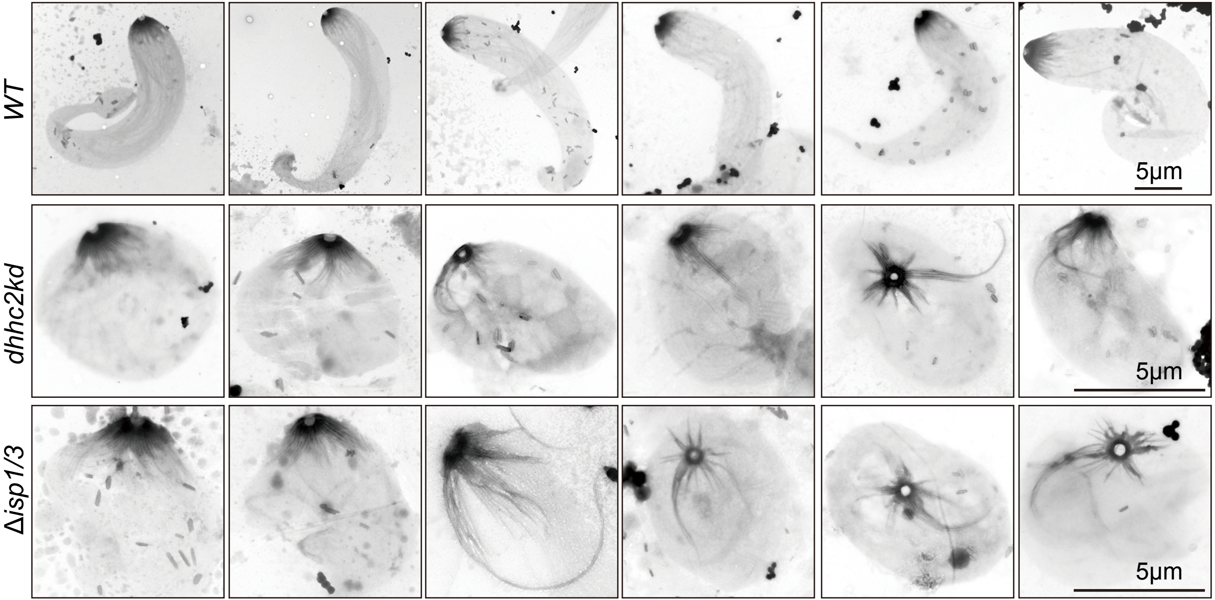

Appendix Figure S5. ISP1/ISP3/DHHC2 maintain the dome-like SPM structure

Gallery of images showing apical cytoskeleton including apical polar ring and the emanating microtubules in ookinetes examined by negative staining and Transmission electron microscopy (TEM). A dome-like SPM structure radiating from the apical polar ring was observed in WT ookinetes, but impaired in *dhhc2kd* and Δ*isp1/3* mutants. Scale bar = 5 μm.

Appendix Table S1. List of genetically modified parasite strains in this study

Strain	Parental strain	Description	Resource	Asexual growth in mouse	Gametocytemia (% wildtype)	Ookinete conversion (Average ± SD)	Ookinete morphology	Average day 7 oocysts per mosquito (% wildtype)	Average day 14 salivary gland sporozoites per mosquito (% wildtype)	Transmission to naïve mice (Mouse bitten/Mouse infected)
17XNL	/	Plasmodium yoelii	NIH	normal	/	63.93±5.81	normal	/	/	3/3
**Parasites with gene tagging**										
isp1::6HA	17XNL	Pyisp1 C-terminally tagged with 6HA	supplymentary SI1	normal	115.45	71.2±2.44	normal	99.56	101.36	3/3
isp1::BFP	17XNL	Pyisp1 C-terminally tagged with BFP	supplymentary SI1	normal	125.11	80.3±3.45	normal	nt	nt	3/3
isp1::3V5	17XNL	Pyisp1 C-terminally tagged with 3V5	supplymentary SI1	normal	113.62	68.1±2.43	normal	nt	nt	3/3
isp3::6HA	17XNL	Pyisp3 C-terminally tagged with 6HA	supplymentary SI1	normal	94.53	65.07±5.96	normal	118.77	98.31	3/3
dhhc2::6HA	17XNL	Pydhhc2 C-terminally tagged with 6HA	supplymentary SI1	normal	91.21	65.13±5.15	normal	94.87	nt	3/3
6HA::dhhc2	17XNL	Pydhhc2 N-terminally tagged with 6HA	supplymentary SI1	normal	94.27	71.07±7.13	normal	nt	nt	nt
dhhc2::4Myc	DTS	Pydhhc2 C-terminally tagged with 4Myc	supplymentary SI1	normal	92.61	65.23±3.9	normal	89.67	91.62	3/3
isp1::3V5/isp3::6HA (DTS)	TTS	Pyisp3 C-terminally tagged with 6HA	supplymentary SI1	normal	92.61	65.47±4.49	normal	nt	nt	3/3
isp1::3V5/isp3::6HA/dhhc2::4Myc (TTS)	DTS	Pydhhc2 C-terminally tagged with 4Myc	supplymentary SI1	normal	90.4	58.5±6.14	normal	nt	nt	3/3
dhhc1::6HA	17XNL	Pydhhc1 C-terminally tagged with 6HA	supplymentary SI1	normal	nt	nt	normal	nt	nt	nt
dhhc3::6HA	17XNL	Pydhhc3 C-terminally tagged with 6HA	supplymentary SI1	normal	nt	nt	normal	nt	nt	nt
dhhc4::6HA	17XNL	Pydhhc4 C-terminally tagged with 6HA	supplymentary SI1	normal	nt	nt	normal	nt	nt	nt
dhhc5::6HA	17XNL	Pydhhc5 C-terminally tagged with 6HA	supplymentary SI1	normal	nt	nt	normal	nt	nt	nt
dhhc6::6HA	17XNL	Pydhhc6 C-terminally tagged with 6HA	supplymentary SI1	normal	nt	nt	normal	nt	nt	nt
dhhc7::6HA	17XNL	Pydhhc7C-terminally tagged with 6HA	supplymentary SI1	normal	nt	nt	normal	nt	nt	nt
dhhc8::6HA	17XNL	Pydhhc8 C-terminally tagged with 6HA	supplymentary SI1	normal	nt	nt	normal	nt	nt	nt
dhhc9::6HA	17XNL	Pydhhc9 C-terminally tagged with 6HA	supplymentary SI1	normal	nt	nt	normal	nt	nt	nt
dhhc10::6HA	17XNL	Pydhhc10 C-terminally tagged with 6HA	supplymentary SI1	normal	nt	nt	normal	nt	nt	nt
dhhc11::6HA	17XNL	Pydhhc11 C-terminally tagged with 6HA	supplymentary SI1	normal	nt	nt	normal	nt	nt	nt
dhhc2kd	17XNL	Pydhhc2 promoter replaced with Pyclag	supplymentary SI1	normal	nt	nt	stumpy and round	nt	nt	nt
dhhc2::6HA/dhhc2kd	dhhc2::6HA	Pydhhc2 promoter replaced with Pyclag	supplymentary SI1	normal	nt	nt	stumpy and round	nt	nt	nt
isp1::3V5/isp3::6HA/dhhc2::4Myc/dhhc2kd	TTS	Pydhhc2 promoter replaced with Pyclag	supplymentary SI1	normal	98.35	63.9±6.95	stumpy and round	0	0	3/0
**Parasites with gene knockout**										
Δisp1	17XNL	Deleted the whole coding sequences of Pyisp1	supplymentary SI1	normal	92.83	69.87±3.82	normal form reduced	10	8	3/1
Δisp1	DTS	Deleted the whole coding sequences of Pyisp1	supplymentary SI1	normal	97.22	60.53±7.51	normal form reduced	nt	nt	nt
Δisp1	ANKA	Deleted the whole coding sequences of Pbisp1	supplymentary SI1	normal	nt	nt	nt	nt	nt	nt
Δisp3	17XNL	Deleted the whole coding sequences of Pyisp3	supplymentary SI1	normal	83.71	65.63±4.06	normal	78	82	3/2
Δisp3	DTS	Deleted the whole coding sequences of Pyisp3	supplymentary SI1	normal	nt	nt	normal	nt	nt	nt
Δisp1/3	Δisp1	Deleted the whole coding sequences of Pyisp3	supplymentary SI1	normal	107.95	74.23±4.74	stumpy and round	0	0	3/0
Δcdpk4	dhhc2::6HA	Deleted the whole coding sequences of Pycdpk4	supplymentary SI1	normal	80.9	65.47±4.49	no zygote	nt	nt	nt
Δhap2	dhhc2::6HA	Deleted the whole coding sequences of Pyhap2	supplymentary SI1	normal	79.26	75.50±2.71	stumpy and round	nt	nt	nt
Δdozi	dhhc2::6HA	Deleted the whole coding sequences of Pydozi	supplymentary SI1	normal	76.29	65.83±6.55	round	0	0	nt
**Parasites with gene compelementation**										
Δisp1/3/Pyisp1::3V5	Δisp1/Δisp3	Plasmid based complemtation of 3V5	supplymentary SI1	normal	nt	nt	normal	nt	nt	nt
Δisp1/3/Pfyisp1::3V5	Δisp1/Δisp3	Plasmid based complemtation of 3V5	supplymentary SI1	normal	nt	nt	normal	nt	nt	nt
Δisp1/3/Pyisp3::6HA	Δisp1/Δisp3	Plasmid based complemtation of 6HA	supplymentary SI1	normal	nt	nt	normal	nt	nt	nt
Δisp1/3/Pfisp3::6HA	Δisp1/Δisp3	Plasmid based complemtation of 6HA	supplymentary SI1	normal	nt	nt	normal	nt	nt	nt
Δisp1/3/Pyisp1^{C7AC8A}::3V5	Δisp1/Δisp3	Plasmid based complemtation of 3V5	supplymentary SI1	normal	nt	nt	stumpy and round	nt	nt	nt
isp1::3V5/isp3::6HA/dhhc2::4Myc/dhhc2kd /Pydhhc2::4Myc	TTS/dhhc2kd	Plasmid based complemtation of 4Myc tagged pydhch2 of pfdhhc2 sequence	supplymentary SI1	normal	nt	nt	normal	nt	nt	nt
isp1::3V5/isp3::6HA/dhhc2::4Myc/dhhc2kd /Pfdhhc2::4Myc	TTS/dhhc2kd		supplymentary SI1	normal	nt	nt	normal	nt	nt	nt
isp1::3V5/isp3::6HA/dhhc2::4Myc/dhhc2kd /Pydhhc2^{C128A}::4Myc	TTS/dhhc2kd		supplymentary SI1	normal	nt	nt	stumpy and round	nt	nt	nt
isp1::3V5/isp3::6HA/dhhc2::4Myc/dhhc2kd /Pydhhc2-C4A::4Myc	TTS/dhhc2kd		supplymentary SI1	normal	nt	nt	stumpy and round	nt	nt	nt
**Parasite with transient transfection**										
BFP::3V5	17XNL	episomal expression	supplymentary SI1	normal	nt	nt	normal	nt	nt	nt
ISP1::BFP::3V5	17XNL	episomal expression	supplymentary SI1	normal	nt	nt	normal	nt	nt	nt
ISP1_N20::BFP::3V5	17XNL	episomal expression	supplymentary SI1	normal	nt	nt	normal	nt	nt	nt
ISP3::BFP::3V5	17XNL	episomal expression	supplymentary SI1	normal	nt	nt	normal	nt	nt	nt
ISP3_N20::BFP::3V5	17XNL	episomal expression	supplymentary SI1	normal	nt	nt	normal	nt	nt	nt
ISP1^{G2A}::BFP::3V5	17XNL	episomal expression	supplymentary SI1	normal	nt	nt	normal	nt	nt	nt
ISP1^{C7AC8A}::BFP::3V5	17XNL	episomal expression	supplymentary SI1	normal	nt	nt	normal	nt	nt	nt
ISP1^{G2AC7AC8A}::BFP::3V5	17XNL	episomal expression	supplymentary SI1	normal	nt	nt	normal	nt	nt	nt
DHHC2::6HA	17XNL	episomal expression	supplymentary SI1	normal	nt	nt	normal	nt	nt	nt
DHHC2::6HA/ΔC1:210-232	17XNL	episomal expression	supplymentary SI1	normal	nt	nt	normal	nt	nt	nt
DHHC2::6HA/ΔC2:233-262	17XNL	episomal expression	supplymentary SI1	normal	nt	nt	normal	nt	nt	nt
DHHC2::6HA/ΔC3:263-284	17XNL	episomal expression	supplymentary SI1	normal	nt	nt	normal	nt	nt	nt
DHHC2::6HA/C4A	17XNL	episomal expression	supplymentary SI1	normal	nt	nt	normal	nt	nt	nt
DHHC2::6HA/C128A	17XNL	episomal expression	supplymentary SI1	normal	nt	nt	normal	nt	nt	nt
DHHC2::6HA/C255A	17XNL	episomal expression	supplymentary SI1	normal	nt	nt	normal	nt	nt	nt
DHHC2::6HA/C258A	17XNL	episomal expression	supplymentary SI1	normal	nt	nt	normal	nt	nt	nt
DHHC2::6HA/C260A	17XNL	episomal expression	supplymentary SI1	normal	nt	nt	normal	nt	nt	nt

DHHC2::6HA/C262A	17XNL	episomal expression	supplymentary SI1	normal	nt	nt	normal	nt	nt	nt
DHHC2::6HA/C258AC255A	17XNL	episomal expression	supplymentary SI1	normal	nt	nt	normal	nt	nt	nt
DHHC2::6HA/C258AC260A	17XNL	episomal expression	supplymentary SI1	normal	nt	nt	normal	nt	nt	nt
DHHC2::6HA/C258AC262A	17XNL	episomal expression	supplymentary SI1	normal	nt	nt	normal	nt	nt	nt
ISP1::HA	17XNL	episomal expression	supplymentary SI1	normal	nt	nt	normal	nt	nt	nt

nt: not tested

Appendix Table S2. Primers and oligonucleotides used in this study

Oligo sequence for gene knockout plasmid construction

Gene name	Gene ID	Gene size (bp)/deleted gene size (bp)	Left homologous arm		Right homologous arm		Target site of sgRNA	
			Forward primer	Reverse primer	Forward primer	Reverse primer	Oligo (Forward)	Oligo (Reverse)
isp1	PY17X_1212600	1021/1021	CATGCCATGGGAAATATATGATACATTAGCATAACATG	CCGGAATTCCCCATTTTGTCGTTATCTGAT	CCGGAATTCGCCAAATTAATAAATATTTGCTTTATC	CCCCTTAAGTGTTTCTATAGACATATTCTCCTAT	TATTGCGTTACATTTAGATGATAG	AAACCTATCATCTAAATGTAACGC
isp1	PBANKA_1209400	1006/1006	CCCGGTACCCACAGAAAATAAAGCAATGT	CCCCCATGGCCATTTTGTCGTTATCTGAT	CCGCTCGAGCCACTATTTTCGCTACAGT	CCCGAATTCGTTTAAAAATGCATGAAGCT	TATTGCGTTACATTTAGATGATAG	AAACCTATCATCTAAATGTAACGC
isp3	PY17X_1328100	453/453	CCCAAGCTTTACATACATTGTTTAAGTCC	CATGCCATGGTGTGTGTAAAGTGCGTATCG	CCGCTCGAGGCACAGAAAATAATAATTCACAC	CCGGAATTCGACAAATAATAAGGATAGGA	TATTGCCTTTGCAATGGATAGTCC	AAACGGACTATCCATTGCAAAGGC
dhhc2	PY17X_0109900	1218/1218	CGGGGTACCCCATAATCCATAATATTTACAGCTCT	CATGCCATGGTGGGGGTGAATTTATTTTATCTGT	CCGCTCGAGGAGCTTATAACATTTCATAA	CCGGAATTCGTGTGTGTGTGTGTATAAATA	TATTGGAAAAGTTCCGAGAAATTG	AAACCAATTTCTCGGAACTTTTCC
cdpk4	PY17X_0617900	1792/1792	CCCAAGCTTTTCGTTACCTGTGCGTAAAGT	CATGCCATGGAATGCTTGGCAAAAGATGATTT	CCGCTCGAGGGTTTTCAGCCCAATTTCAT	CCGGAATTCAATGCTTGGCAAAAGATGATTT	TATTGAGGTTGATCAGAATAACGA	AAACTCGTTATTCTGATCAACCTC
hap2	PY17X_1215800	2698/2698	CGGGGTACCGATCATGTTGGAGAATAAATGG	CATGCCATGGCAATTTATATATGTGCTTCGACC	CCGCTCGAGCATGGAATAATATTTGCAAGTTTG	CCCCTTAAGTAGGTGTGCAATTTTGTATGC	TTAAAAGTGATGAGAAAAGG	CCTTTTCTCATCACTTTTAA
dozi	PY17X_1220900	1507/1507	CGGGGTACCCCTGTTTAGGCTTTATATTGTT	CATACCATGGATTAGCATTTGAAGCGACACAAT	CCGCTCGAGGCAGTATACTACATCATAATGA	CCCCTTAAGTCTGGATATATAAGTTGCACTAT	TATTAGTATTCCTGTGGCTTTGGC	AAACGCCAAAGCCACAGGAATACT

Oligo sequence for gene knockdonw plasmid construction (promoter swap method)

Gene name	Gene ID	putative promoter(bp) replaced/deleted	Left homologous arm		Right homologous arm		Target site of sgRNA	
			Forward primer	Reverse primer	Forward primer	Reverse primer	Oligo (Forward)	Oligo (Reverse)
dhhc2	PY17X_0109900	1808/1319	CCCAAGCTTTGTCGTTTGATAGTGGTTTAT	CGGGTACCAGTGCATACGAATATATGCAT	CCCCATGGCAATGACACATAAATATATGCAGAT	CCCCTTAAGTTCTATCTGGCTTCCATACAT	TATTGGAAAAGTTCCGAGAAATTG	AAACCAATTTCTCGGAACTTTTCC

Primers for PCR-genotyping parasite with gene knockout and knockdown

Gene name	Gene ID	P1	P2	P3	P4	P5	P6	
isp1	PY17X_1212600	AATAATATTATAGAGCAATTTAAAATAG	ATAATAGTAGTAGTAGTAGTAATAGCA	CAGATAACGACAAAATGGGG	GTGCTTATTCTTTTGGAGTTAGTCA	AATAATATTATAGAGCAATTTAAAATAG	AAACCATATTATCTACTAATATAC	
isp1	PBANKA_1209400	AAAGCTATTCATGTTTCTAT	AATAAGCAAATAAAGTTGGT	GATAATCAGATAACGACAAA	TCTTTTGGAGTTATAGTCAT	ATGTATATCCTGCCACCAAA	CGAAACTGTAGCGAAAATAG	
isp3	PY17X_1328100	GTAGCACAATTCAATATGGT	CACCACTTGGACACCATTGT	TTCGATACGCACTTTACACAC	ATTGGATATAATACATGCTG	TGGATAGATAAATATAAGAA	GGACTATCCATTGCAAAGGC	
dhhc2(KO)	PY17X_0109900	CTTGGATAAAGATATTTTGGAAT	GTGTGTGTGTGTAAATGAAA	CATAATCCATAATATTTACAGCTCT	TATTCATTTGGCTTTCTGTG	ATTAATGCATATATGGATAC	GTGTGTGTGTGTAAATGAAA	
dhhc2(KD)	PY17X_0109900	TGTTTGTGTACCTTGCCTAT	GAATCGTAGCACATATGCTT	GGAGAACAAATAAGATCGT	ACATTTGCAAAGCTTTAGAT	GGAGAACAAATAAGATCGT	CATTGATTTGGGGGTGAATT	
cdpk4	PY17X_0617900	GTATCACTGTATATGTAATAT	GTATTGAATAGTGGATGCAAAT	CCATAAATCATCTTTTGCCA	CATTATTTTATTATCATGGA	GTATCACTGTATATGTAATAT	GAACTTAAAATTTGATCAACT	
hap2	PY17X_1215800	TATTTCAAGACAAAACACCT	GAAGAGGACGATTTTTCTTA	AATTTAAGGTCGAAGCACAT	TTTGGTTTTTAGAGGACCTA	TTTATATAAAGACAAAAACG	CGTGTATATTCATGAAATTT	
dozi	PY17X_1220900	TCTGTACACATAAAACTCGT	ATTGAAGCCGCAATGTGTAT	ATACTATCCACACGTTTCAT	ATATTCTCCTATTTGCACGT	TCTGTACACATAAAACTCGT	AGTAACGGAATAGCAAATGC	

Oligo sequences for gene tagging plasmid construction

Gene name	Tag	Gene ID	Left homologous arm		Right homologous arm		Target site of sgRNA	
			Forward primer	Rerverse primer	Forward primer	Reverse primer	Oligo (Forward)	Oligo (Reverse)
isp1	C-terminal 6HA	PY17X_1212600	CGGGGTACCCAACAGAATAATGATAACAT	CATGCCATGGATTTTTTTTATAATCTCTCAT	CCGGAATTCGCCAAATTAATAAATATTTGCTTTATC	CCCCTTAAGTGTTTCTATAGACATATTCTCCTATT	TATTCCATTCCATTAAATTATATA	AAACTATATAATTTAATGGAATGG
isp1	C-terminal 3V5	PY17X_1212600	CGGGGTACCCAACAGAATAATGATAACAT	CATGCCATGGATTTTTTTTATAATCTCTCAT	CCGGAATTCGCCAAATTAATAAATATTTGCTTTATC	CCCCTTAAGTGTTTCTATAGACATATTCTCCTATT	TATTCCATTCCATTAAATTATATA	AAACTATATAATTTAATGGAATGG
isp1	C-terminal BFP	PY17X_1212600	CGGGGTACCCAACAGAATAATGATAACAT	CATGCCATGGATTTTTTTTATAATCTCTCAT	CCGGAATTCGCCAAATTAATAAATATTTGCTTTATC	CCCCTTAAGTGTTTCTATAGACATATTCTCCTATT	TATTCCATTCCATTAAATTATATA	AAACTATATAATTTAATGGAATGG
isp3	C-terminal 6HA	PY17X_1328100	CGGGGTACCTGGGAAACAGCTTGTGCTGC	CATGCCATGGAGCAGTTAAGCAATATTTGT	CCGCTCGAGGCACAGAAAATAATAATTCACAC	CCGGAATTCGACAAATAATAAGGATAGGA	TATTTGGATAGATAAATATAAGAA	AAACGGCTTATATTTATCTATCC
dhhc1	C-terminal 6HA	PY17X_0404200	CGGGGTACCGCACCCCTATATATTAATGC	CATGCCATGGTACGGTATCTGGAACTAGC	CCGCTCGAGGGGGTTACCCAATCTTATT	CCCCTTAAGCGGTACGGTATTAAAACGAA	TATTGGAAAGTGGAAAGTGGAAAG	AAACCTTTCCACTTTCCACTTTCC
dhhc2	C-terminal 6HA	PY17X_0109900	CGGGGTACCGCTACCCAAACTTTTCATTATAT	CATGCCATGGGTATATTATTTCCATGTGATATGC	CCGCTCGAGGAGCTTATAACATTTCATAA	CCGGAATTCGTGTGTGTGTGTGTATAAATA	TATTTGGAGAGTTAGCATATCACA	AAACTGTGATATGCTAACTCTCCA
dhhc3	C-terminal 6HA	PY17X_0929300	CGGGGTACCGTGGTTATTTTACGGCATAT	CATGCCATGGTATTTTATAGACATTTCAGTTTCCAC	CCGCTCGAGCGAAATATGATCAATAATATATATGG	CCCCTTAAGGAAGCTACAAAATGAAGTTA	TATTGGATATAGAAAACCCCAAAG	AAACCTTTGGGGTTTTCTATATCC
dhhc4	C-terminal 6HA	PY17X_1422600	CGGGGTACCGATTCAAAAATATCACTAGCATATC	CATGCCATGGATATTCGTTATCATTATTAGATGG	CCGCTCGAGTCTTAAATAAACTTTCTATGTTAT	CCCCTTAAGTTGGGGCACATTTTATCTTT	TATTTCTTTCCCAAATAAATAATA	AAACTATTATTTATTTGGGAAAGA
dhhc5	C-terminal 6HA	PY17X_1342500	CGGGGTACCGTATGGGCATAGATAATGCG	CATGCCATGGATCATGTTTCTCATAAAATTGGC	CCGCTCGAGTGAAAAATACCAAAGCGTTGA	CCCCTTAAGAACACTCATTTGTTATGCTCA	TATTCAAGTAAGACAGTTTGTAGA	AAACTCTACAAACTGTCTTACTTG
dhhc6	C-terminal 6HA	PY17X_0836700	CGGGGTACCGTTCTCTTCTATTTTATCAGGT	CATGCCATGGAAATTGAGAAATAACACAAAGTTTT	CCGCTCGAGGAACCAAATCATCAAGTAATT	CCCCTTAAGCTCTGTTAGAAGCTGTTTAT	TATTATGGGATGTGTACATACATA	AAACTATGTATGTACACATCCCAT
dhhc7	C-terminal 6HA	PY17X_1246400	CGGGGTACCCCATAACCAGTCTTACTCAG	CATGCCATGGATTATTTATGTTTATTGGCATTG	CCGCTCGAGGCTTTAAATAGGCTTTTGAA	CCCCTTAAGGATATATATTAGTTGGACGGAT	TATTTTAGTATATAGTAACAAGAA	AAACTTCTTGTTACTATATACTAA
dhhc8	C-terminal 6HA	PY17X_1421400	CGGGGTACCCCACATTTTGGCATTTGTT	CATGCCATGGCAATGTTACTTTAACACATTCT	CCGCTCGAGATGAAAACACAGAAACTTTT	CCCCTTAAGGATACGCCACCATGTTTAAA	TATTACGACATATGAAGATATTCA	AAACTGAATATCTTCATATGTCGT
dhhc9	C-terminal 6HA	PY17X_0934100	CGGGGTACCCCAGAATCCCTATGACATTG	CATGCCATGGATCGTCTTCTTTCAAATTAAAAT	CCGCTCGAGGATCAAGCACGATCCATATA	CCCCTTAAGCGTATATTCATGTAGGGGATT	TATTACAAATTGTCCGGATTGAAC	AAACGTTCAATCCGGACAATTTGT
dhhc10	C-terminal 6HA	PY17X_0513100	CGGGGTACCGTATGCCCATTTAATAGCTAGC	CATGCCATGGTAATGTTTTATAAAATAGTCCATTCCC	CCGCTCGAGATCACATTTTTGTGTTGGA	CCCCTTAAGACAAGTTGCATATGCAGTT	TATTGGAAAACTTTAAGCAAGTTT	AAACAAACTTGCTTAAAGTTTTCC
dhhc11	C-terminal 6HA	PY17X_0313100	CGGGGTACCTTGGGGAATAAAAACCAACAC	CATGCCATGGTATTTTTTTTTTATTTTCATTAAAATCGTC	CCGCTCGAGATACCAGGTAGCTAGTTTGT	CCCCTTAAGCCAAGCATATAAAAAGGAAGA	TATTCCCAGTTGATGCAAAAATCA	AAACTGATTTTTGCATCAACTGGG
dhhc2	C-terminal 4Myc	PY17X_0109900	CGGGGTACCGCTACCCAAACTTTTCATTATAT	CATGCCATGGGTATATTATTTCCATGTGATATGC	CCGCTCGAGGAGCTTATAACATTTCATAA	CCGGAATTCGTGTGTGTGTGTGTATAAATA	TATTTGGAGAGTTAGCATATCACA	AAACTGTGATATGCTAACTCTCCA
dhhc2	N-terminal 6HA	PY17X_0109900	CGGGGTACCTCGTAATACATTTATTATTTAAGT	CATGCCATGGTTTTTATATTGGTTGTGGTT	CCGCTCGAGCAATGACACATAAATATATGCAGAT	CCCCTTAAGTTCTATCTGGCTTCCATACAT	TATTGGAAAAGTTCCGAGAAATTG	AAACCAATTTCTCGGAACTTTTCC

Primer sequence for PCR-genotyping parasite with gene tagging

Gene name	Tag	Gene ID	P7	P8	P9	P10		
isp1	C-terminal 6HA	PY17X_1212600	GCATACGTTACAATTTTGAG	CGAAATTGAGCATGTAAAATA	CGTTACATTTAGATGATAGT	GTGCTTATTCTTTTGGAGTTA		
isp1	C-terminal BFP	PY17X_1212600	GCATACGTTACAATTTTGAG	CGAAATTGAGCATGTAAAATA	CGTTACATTTAGATGATAGT	GTGCTTATTCTTTTGGAGTTA		
isp1	C-terminal 3V5	PY17X_1212600	GCATACGTTACAATTTTGAG	CGAAATTGAGCATGTAAAATA	CGTTACATTTAGATGATAGT	GTGCTTATTCTTTTGGAGTTA		
isp3	C-terminal 6HA	PY17X_1328100	TTCGATACGCACTTTACACA	CACCACTTGGACACCATTGTAA	CCTGGAGAGGAAAGAATGTT	ATTGGATATAATACATGCTG		
dhhc1	C-terminal 6HA	PY17X_0404200	CTTATTTGCACGTAACACAA	CGGTACGGTATTAAAACGAA	GCACCCCTATATATTAATGC	TAAAACGGGCTTGTAGTTAT		
dhhc2	C-terminal 6HA	PY17X_0109900	CCAGATAGAACACATCATTG	GAAAGGGAATTGAAAACTTC	TTTCGAATAGACCAGCAGGA	TATTCATTTGGCTTTCTGTG		
dhhc3	C-terminal 6HA	PY17X_0929300	CCGATCTAACGTACGAATAA	GAAGCTACAAAATGAAGTTA	GTGGTTATTTTACGGCATA	CAATTGATGCAAATTCTTCT		
dhhc4	C-terminal 6HA	PY17X_1422600	GGAATGCAAAAATGTTATCT	TTGGGGCACATTTTATCTTT	GATTCAAAAATATCACTAGCATATC	GCAAAACTAGTTAATTATCA		
dhhc5	C-terminal 6HA	PY17X_1342500	GCATTGATATATTTGATCAT	AACACTCATTTGTTATGCTCA	GTATGGGCATAGATAATGCG	TTCTACACTATTTGCAATCT		
dhhc6	C-terminal 6HA	PY17X_0836700	CATACCTACCTTTGTATGGT	CTCTGTTAGAAGCTGTTTAT	GTTCTCTTCTATTTTATCAGGT	CATCTTTTCGAAGAGTTAGT		
dhhc7	C-terminal 6HA	PY17X_1246400	TTTAGACCTAATTTGGCAAT	GATATATATTAGTTGGACGGAT	CCATAACCAGTCTTACTCAG	GCAAAGGACACAAATATTCT		
dhhc8	C-terminal 6HA	PY17X_1421400	CATATAGACAAAACACCATC	GATACGCCACCATGTTTAAA	CCACATTTTGGCATTTGTT	GTGTGATTGTTATTCTTCAT		
dhhc9	C-terminal 6HA	PY17X_0934100	TGTAGAAACCTGAAACCAAT	CGTATATTCATGTAGGGGATT	CCAGAATCCCTATGACATTG	GTTCCATTCGAAGTATACAT		
dhhc10	C-terminal 6HA	PY17X_0513100	CCTAGAAATTGTTTTATCGGCTG	ACAAGTTGCATATGCAGTT	GCCTTGGATATATGTATATGC	GCCTTGGATATATGTATATGC		

dhhc11	C-terminal 6HA	PY17X_0313100	GGTCGTTTCTATATTTTCAT	CCAAGCATATAAAAAGGAAGA	TTGGGGAATAAAAACCAACAC	TATTATATTATGGTGCGCAT		
dhhc2	C-terminal 4Myc	PY17X_0109900	CCAGATAGAACACATCATTG	GAAAGGGAATTGAAAACTTC	TTTCGAATAGACCAGCAGGA	TATTCATTTGGCTTTCTGTG		
dhhc2	N-terminal 6HA	PY17X_0109900	TATGCATATGCATGTATAT	GAATCGTAGCACATATGCTT	GGAAATTAGTTATACTGCTA	ACATTTGCAAAGCTTTAGAT		
**Oligo sequences for dhhc2 mutant plasmid construction**								
**Modification**		**Description**	**PCR based site-mutangenesis forward primer**		**PCR based site-mutangenesis reverse primer**			
dhhc2_ΔC1		deletion in C terminal 210-232 residues	GTTTTAATTTTTGCTTTAGTTCCATTTATATATCATCAAGACTATAA		CATCCTACATTATAAATATTATAGTCTTGATGATATATAAATGGAACTAAAGCAAA			
dhhc2_ΔC2		deletion in C terminal 233-262 residues	TGAAAATATGGATATATATCATCAAGACATGTGCCCATGCC		ATACAATGGCATGGGCACATGTCTTGATGATATATATC			
dhhc2_ΔC3		deletion in C terminal 263-284 residues	TGTGCCCATGCCATTGTATTATATCACATGGAAATAA		gctagcTATATTATTTCCATGTGATATAATACAATGGCATG			
dhhc2_C128A		DHHC(125-128 residues) replaced with DHHA	AGATGTGTATTAAATATGGATCATCATgcTCCATGGATAAAT		ATCCAACACAATTATTTATCCATGGAgcATGATGATCCAT			
dhhc2_C255A		CWMCPCHC(255-262) replaced with AWMCPCHC	AAGCAGGTTTTTGGAAATAACATTTTAgcCTGGATGTGC		GCATGGGCACATCCAGgcTAAAATGTTATTTCC			
dhhc2_C258A		CWMCPCHC(255-262) replaced with CWMAPCHC	GGAAATAACATTTTATGCTGGATGgcCCCATGCCAt		TTCGAAATACAATGGCATGGGgcCATCCAGCAT			
dhhc2_C260A		CWMCPCHC(255-262) replaced with CWMCPAHC	TTATGCTGGATGTGCCCAgcCCATTGTATTTCG		GCTGGTCTATTCGAAATACAATGGgcTGGGCACATC			
dhhc2_C262A		CWMCPCHC(255-262) replaced with CWMCPCHA	GATGTGCCCATGCCATgcTATTTCGAATAGAC		CTCCTGCTGGTCTATTCGAAATAgcATGGCATGGG			
humanized dhhc2_C128A		DHHC(125-128 residues) replaced with DHHA	GAACATGGACCACCACTcCCCTTGGATCA		GCAGTTATTGATCCAAGGGgAGTGGTGGTC			
humanized hdhhc2_C255A		CWMCPCHC(255-262) replaced with AWMCPCHC	TTCGGCAACAACATTCTGTcCTGGATGTGC		GCAAGGGCACATCCAGgACAGAATGTTGT			
humanized dhhc2_C258A		CWMCPCHC(255-262) replaced with CWMAPCHC	AACATTCTGTGCTGGATGTcCCCTTGCCAC		GATGCAGTGGCAAGGGgACATCCAGCAC			
humanized dhhc2_C260A		CWMCPCHC(255-262) replaced with CWMCPAHC	GATGCAGTGGCAAGGGgACATCCAGCAC		GTCTATTGGAGATGCAGTGGgAAGGGCACATC			
humanized dhhc2_C262A		CWMCPCHC(255-262) replaced with CWMCPCHA	ATGTGCCCTTGCCACTcCATCTCCAATAGAC		GGCGGGTCTATTGGAGATGgAGTGGCAAGG			
humanized dhhc2_Cter75		DHHC2 C terminal 210-284 residues	GAGCTGTACAAGctcgagACAAAGTTCCACCT		AGCTTGAGGTGGAACTTTGTctcgagCTTGT			
**Primers for RT-qPCR**								
**Gene name**	**Gene ID**	**Forward Primer**	**Reverse Primer**	**Product Size(bp)**				
18s rRNA	PY17X_0522400	GGTTTTATAATTGGAATGATGGGAAT	ACGCTATTGGAGCTGGAATTACC	101				
Arg_tRNAs	PY17X_1436600	TGCAAACAAGCAGAATGGGTTG	TTCTTTGCCCTTTCAGTGCC	155				
isp1	PY17X_1212600	GATGGAACCAAATTACCTTGT	ACGCCAAACAACAATTGCCA	184				
isp3	PY17X_1328100	ATGGGAAACAGCTTGTGCT	TCAGGAAATGCTACTCTAAT	167				
**Primers for plasmid complementation**								
**Gene name**	**Gene ID**	**CDS**		**5'UTR**		**3'UTR**		
		**Forward Primer**	**Reverse Primer**	**Forward Primer**	**Reverse Primer**	**Forward Primer**	**Reverse Primer**	
Pyisp1	PY17X_1212600	CATGCCATGGATGGGGAATATTGTATCCTG	CTAGCTAGCATTTTTTTTATAATCTCTCA	GCGGGATCCTGTCTAAAGGAAGAGCTTGT	CATGCCATGGTTTGTCGTTATCTGATTATCTT	CTAGCTAGCAAAATTGATAAGTTAACAGC	CCCCTTAAGCGACGAATGTATGGCCCTACAT	
Pfisp1	PF3D7_1011000	CATGCCATGGATGGGGAATATTGTATCATG	CTAGCTAGCCGAATTTTTTTTATAATCTT	GCGGGATCCTGTCTAAAGGAAGAGCTTGT	CATGCCATGGTTTGTCGTTATCTGATTATCTT	CTAGCTAGCAAAATTGATAAGTTAACAGC	CCCCTTAAGCGACGAATGTATGGCCCTACAT	
Pyisp3	PY17X_1328100	ctaGCTAGCATGGGAAACAGCTTGT	ccgGAATTCAGCAGTTAAGCAATATTTGT	CCCCATGGCCTAGTTGACGAAACTACAACT	CCGGTACCTTTGAAAATTAAAAAAAAAAAAAGAGTT	CCGAATTCgCCGGTTCTGCTGCTAGATA	CCCTTAAGGCACCAGTTTATGCGAATGCAT	
Pfisp3	PF3D7_1460600	ctaGCTAGCATGGGAAATTTATGTTGTAGT	ccgGAATTCTGCATTCAAACAATATTTTTGT	CCCCATGGCCTAGTTGACGAAACTACAACT	CCGGTACCTTTGAAAATTAAAAAAAAAAAAAGAGTT	CCGAATTCgCCGGTTCTGCTGCTAGATA	CCCTTAAGGCACCAGTTTATGCGAATGCAT	
Pydhhc2	PY17X_0109900	CATGCCATGGATGGGGAATATTGTATCCTG	CTAGCTAGCATTTTTTTTATAATCTCTCA	GCGGGATCCTGTCTAAAGGAAGAGCTTGT	CATGCCATGGTTTGTCGTTATCTGATTATCTT	CTAGCTAGCAAAATTGATAAGTTAACAGC	CCCCTTAAGCGACGAATGTATGGCCCTACAT	
Pfdhhc2	PF3D7_0609800	CATGCCATGGATGAGACCTAAATATGTTCAAGCT	CTAGCTAGCAACAGGATTTTCATGTGCTA	GCGGGATCCTGTCTAAAGGAAGAGCTTGT	CATGCCATGGTTTGTCGTTATCTGATTATCTT	CTAGCTAGCAAAATTGATAAGTTAACAGC	CCCCTTAAGCGACGAATGTATGGCCCTACAT	
