# Supplementary material for: A protein palmitoylation cascade regulates microtubule cytoskeleton integrity in Plasmodium
Source: EMBO J. 2020 May 12;39(13):e104168. doi: 10.15252/embj.2019104168 (PMC7327484; doi:10.15252/embj.2019104168)
Supplement: Supplementary file 2 — Expanded View Figures PDF [file EMBJ-39-e104168-s002.pdf]

# Expanded View Figures

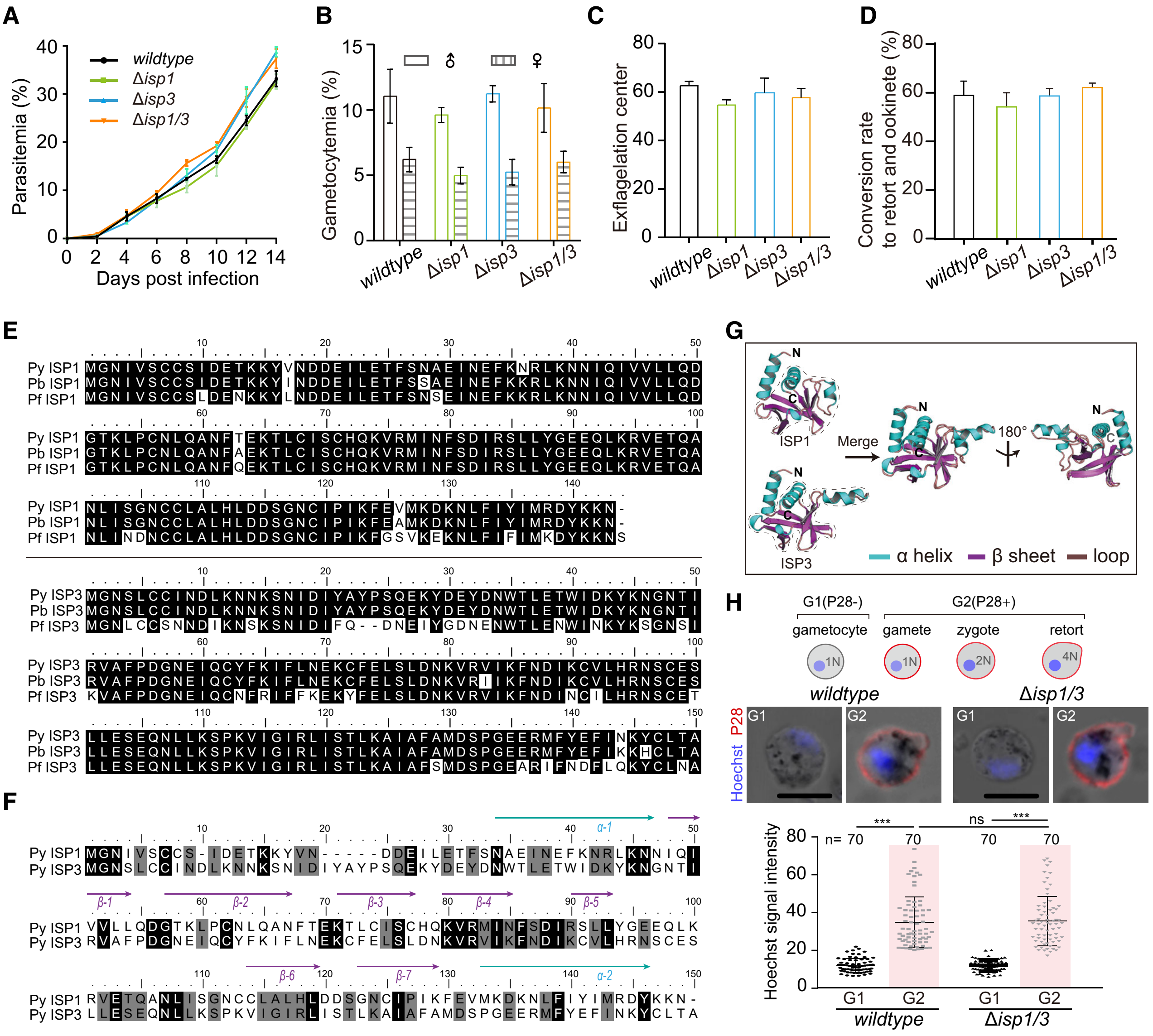


**Figure EV1. Development analysis of the Δ*isp1*, Δ*isp3*, and Δ*isp1/3* parasites and protein sequence analysis of ISP1 and ISP3.**

A Parasitemia in mouse. Values are means ± SEM ($n$ = 3 biological replicates).

B Gametocytemia in mouse. Values are means ± SEM ($n$ = 3 biological replicates).

C Male gametocyte activation *in vitro* by counting the exflagellation centers formed. Values are means ± SEM ($n$ = 3 biological replicates).

D *In vitro* differentiation to retort and ookinete. Values are means ± SEM ($n$ = 3 biological replicates).

E Alignment of ISP1 (up panel) or ISP3 (lower panel) protein sequences from *P. yoelii*, *P. berghei*, and *P. falciparum*. *pyisp1*: PY17X_1212600; *pbisp1*: PBANKA_1209400; *pfisp1*: PF3D7_1011000; *pyisp3*: PY17X_1328100; *pbisp3*: PBANKA_1324300; *pfisp3*: PF3D7_1460600.

F Second structure-based sequence alignment of ISP1 and ISP3 protein of *P. yoelii*.

G Predicted structures of *P. yoelii* ISP1 and ISP3. The structures of *Plasmodium* ISP1 and ISP3 are constructed using homology modeling with SWISS-MODEL based on protein structures of *T. gondii* ISP1 (PDB: 4chm) and ISP3 (PDB: 4chj). Both protein structures of ISP1 and ISP3 display a character of the pleckstrin homology (PH) domain (indicated by dashed line), composing one α-helix and six β-sheets. The α-helix is shown in green, β-sheet in purple, and loop in brown.

H Nuclei DNA content analysis of parasite. Upper panel indicates the schematic of female gametocyte–female gamete–zygote–retort/ookinete differentiation. One female gamete (1N) fertilizes with one male gamete to form zygote (2N) and further develop to retort/ookinete (4N) by meiotic DNA replication. P28 and Hoechst 33342 staining of female gametocyte, female gamete, zygote, and retort of WT and Δ*isp1/3* parasites. Zygotes and retorts were collected at 4 h post-activation. Scale bar = 5 μm. Lower panel indicates the quantification of the Hoechst fluorescence signals. Values are mean ± SD ($n$ is the number of cells measured in each group). Mann–Whitney test, ***$P$ < 0.001. Three biological replicates performed.

© 2020 The Authors

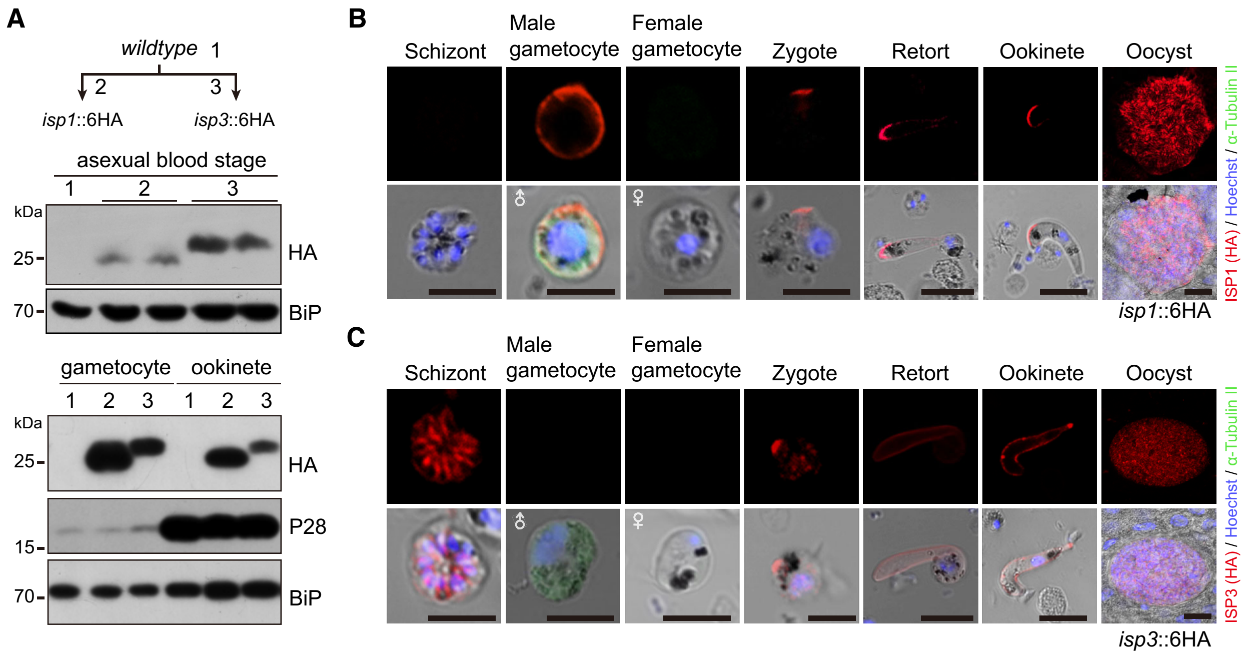


**Figure EV2. Stage expression and cellular localization of ISP1 and ISP3.**

A Western blot of ISP1 and ISP3 in asexual mouse blood stages, gametocytes, and ookinetes of the *isp1::6HA* and *isp3::6HA* parasites. ER protein BiP as loading control. Two lanes in blot are replicates from the same sample.

B, C IFA of ISP1 (B) and ISP3 (C) in mouse and mosquito stages of the *isp1::6HA* and *isp3::6HA* parasites, respectively. Purified gametocytes were stained with antibodies against HA and α-tubulin II (male gametocyte-specific). Scale bar = 5 μm.

Source data are available online for this figure.

 © 2020 The Authors

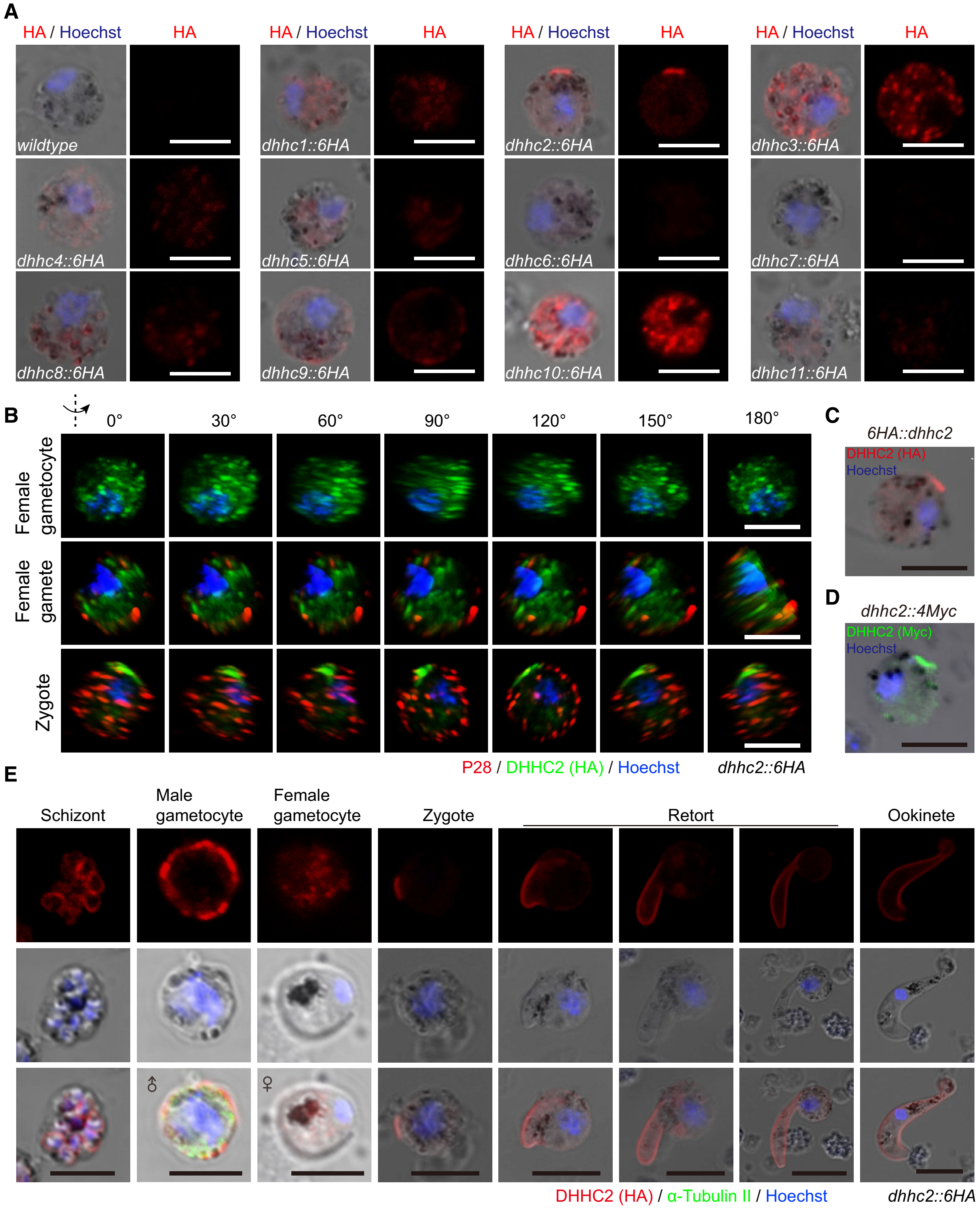
A
HA / Hoechst
HA
wildtype
dhhc1::6HA
dhhc2::6HA
dhhc3::6HA
dhhc4::6HA
dhhc5::6HA
dhhc6::6HA
dhhc7::6HA
dhhc8::6HA
dhhc9::6HA
dhhc10::6HA
dhhc11::6HA
B
0°
30°
60°
90°
120°
150°
180°
Female gametocyte
Female gamete
Zygote
P28 / DHHC2 (HA) / Hoechst
dhhc2::6HA
C
6HA::dhhc2
DHHC2 (HA)
Hoechst
D
dhhc2::4Myc
DHHC2 (Myc)
Hoechst
E
Schizont
Male gametocyte
Female gametocyte
Zygote
Retort
Ookinete
♂
♀
DHHC2 (HA) / α-Tubulin II / Hoechst
dhhc2::6HA

**Figure EV3.**

**Figure EV3. Screening and identification of DHHC2 polarizing at zygotes.**

A IFA of eleven *Plasmodium* palmitoyl-S-acyl-transferase (PAT) proteins in zygotes.
Endogenous protein is tagged C-terminally with a 6HA tag. Only DHHC2 displays polarization at zygote of the *dhhc2::6HA* parasites. Scale bar = 5 μm.

B 3D imaging of DHHC2 and P28 expression in female gametocyte, female gamete, and zygotes of the *dhhc2::6HA* parasite. Scale bar = 5 μm.

C, D IFA of DHHC2 expression in zygotes of another two independent strains *6HA::dhhc2* and *dhhc2::4Myc*. Scale bar = 5 μm.

E IFA of DHHC2 expression in asexual blood stages, gametocytes, and zygote to ookinete of the *dhhc2::6HA* parasite. Purified gametocytes were stained with antibodies against HA and α-tubulin II (male gametocyte-specific). Scale bar = 5 μm.

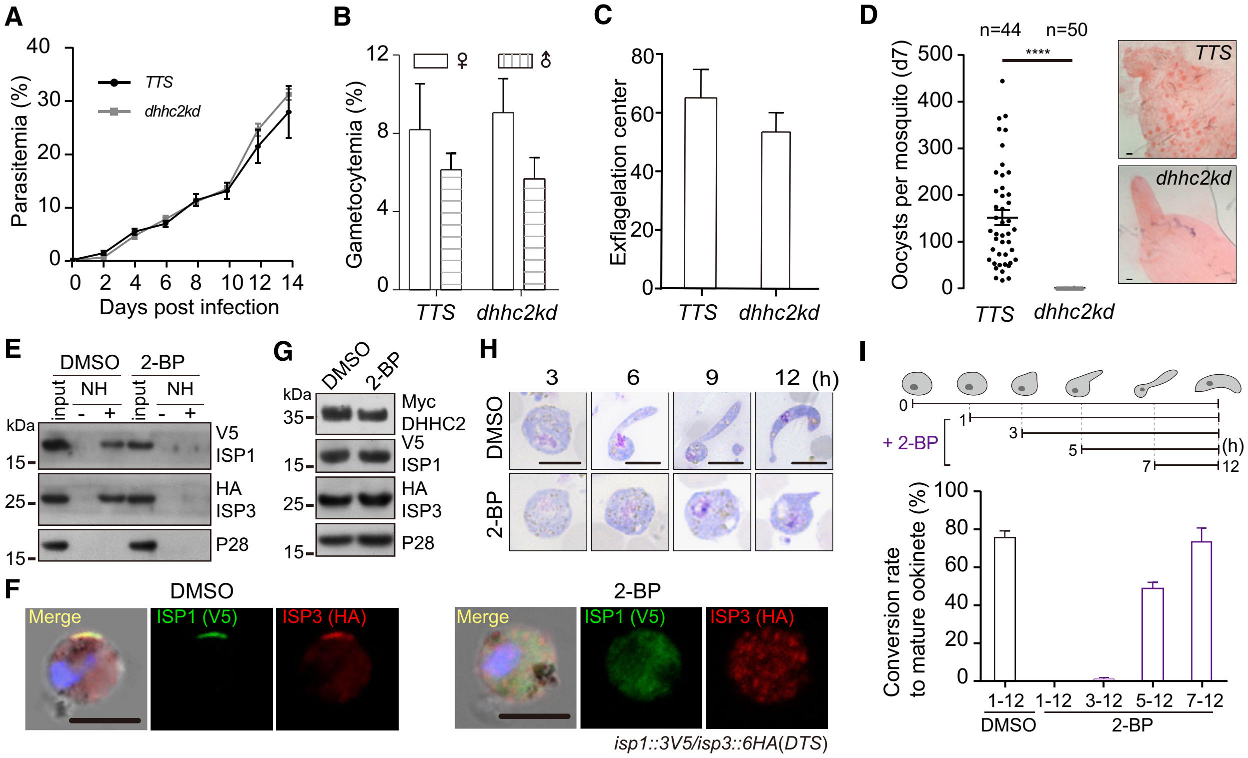


**Figure EV4. DHHC2 palmitoylates ISP1 and ISP3.**

A Parasitemia in mouse. Values are means ± SEM ($n$ = 3 biological replicates).

B Gametocytemia in mouse. Values are means ± SEM ($n$ = 3 biological replicates).

C Male gametocyte activation *in vitro*. Values are means ± SEM ($n$ = 6 biological replicates).

D Midgut oocysts in mosquitoes 7 days post-blood feeding. n is the number of mosquitoes in each group. The horizontal line shows the mean value of each group, Mann–Whitney test, ****$P < 0.0001$. Right panel shows mosquito midguts stained with 0.5% mercurochrome. Scale bar = 50 μm. Three biological replicates performed.

E Acyl-RAC method detecting palmitoylation of ISP1 and ISP3 in *DTS* zygotes treated with 100 μM 2-BP, a potent inhibitor of protein palmitoylation. NH, $NH_2OH$. The data are representative of two repeats.

F IFA of ISP1 and ISP3 in 2-BP-treated *DTS* zygotes. Scale bar = 5 μm.

G Western blot of DHHC2, ISP1, and ISP3 in 2-BP-treated *TTS* zygotes.

H Time-course analysis of ookinete differentiation treated with 2-BP. Scale bar = 5 μm. h, hour.

I Inhibiting effect of 2-BP on ookinete differentiation is time-dependent. 2-BP treatment during early development (1–3 h) damages the ookinete differentiation. Values are means ± SEM ($n$ = 3 biological replicates).

Source data are available online for this figure.

 © 2020 The Authors

**Figure EV5. ISP1 and ISP3 have no effect on IMC formation and apical protrusion.**

A TEM longitudinal sections of WT, *dhhc2kd*, and Δ*isp1/3* parasites collected from 12-h *in vitro* ookinete culture. The IMC is formed underneath the PPM (parasite plasma membrane) in both WT and mutant parasites. Inserts in upper images are magnified to indicate the IMC and apical protrusion. PPM, black; IMC, pink; and apical polar ring, purplish red.

B Expression and localization of GAP45 in WT and Δ*isp1/3* ookinetes from 12-hour *in vitro* culture. Arrowheads point to cell apical. Scale bar = 5 μm.

C *In vitro* ookinete differentiation of WT parasite treated with different concentrations of colchicine, an inhibitor of microtubule polymerization in *Plasmodium*. The data show quantification from two independent repeats, and values are means ± SD.

D Inhibiting effect of colchicine on ookinete differentiation is time-dependent. Colchicine treatment of parasite during early development (1–3 h) blocks ookinete differentiation. The data show quantification from three independent repeats, and values are means ± SD.

E Western blot of α-tubulin, β-tubulin, GAP45, and DHHC2 expression in the *dhhc2::6HA* zygote culture treated with colchicine.

F IFA of P28, GAP45, ISP1, and DHHC2 expression at early (3 h) or later (12 h) ookinete of the colchicine-treated *TTS* parasites. Scale bar = 5 μm.

Source data are available online for this figure.

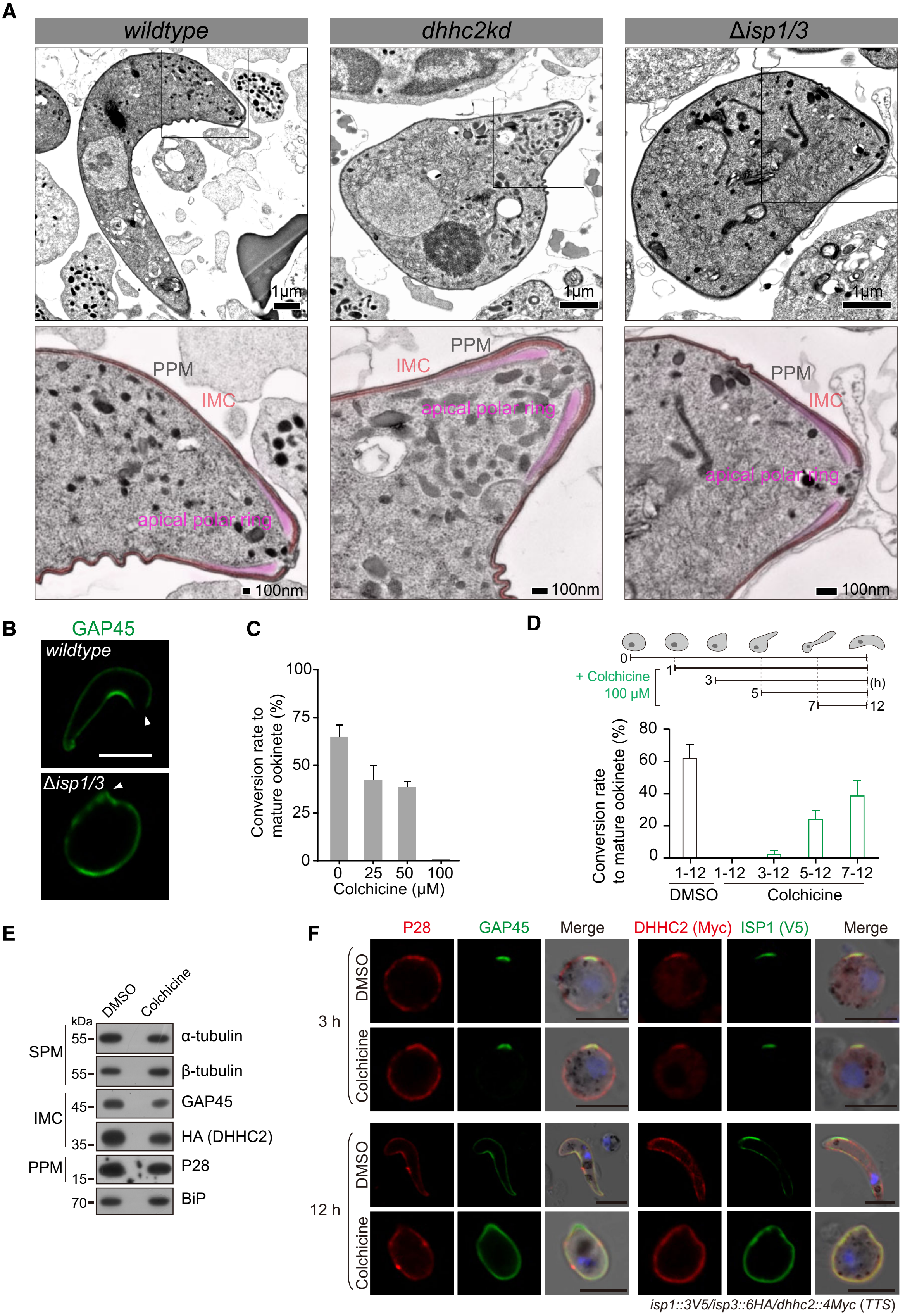


**Figure EV5.**

 © 2020 The Authors
