# Supplementary material for: A protein palmitoylation cascade regulates microtubule cytoskeleton integrity in Plasmodium
Source: EMBO J. 2020 May 12;39(13):e104168. doi: 10.15252/embj.2019104168 (PMC7327484; doi:10.15252/embj.2019104168)
Supplement: Supplementary file 3 — Source Data for Expanded View [file EMBJ-39-e104168-s010.zip › embj2019104168-sup-0010-SDataFigEV/embj2019104168-sup-0011-SDataFigEV4.pdf]

Figure EV4

EV4-E

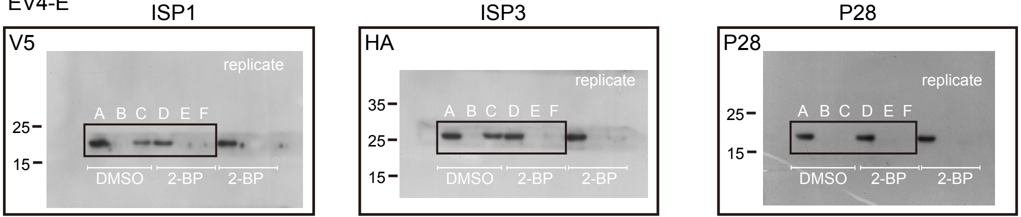

Lane A: DMSO-input
Lane B: DMSO-NH2OH(-)
Lane C: DMSO-NH2OH(+)
Lane D: 2-BP-input
Lane E: 2-BP-NH2OH(-)
Lane F: 2-BP-NH2OH(+)

EV4-G

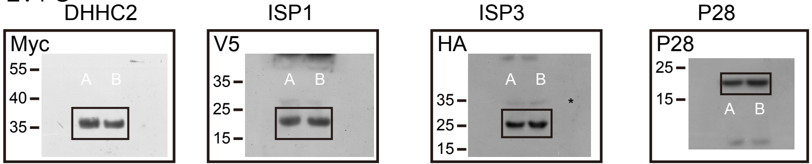

Lane A: TTS+DMSO
Lane B: TTS+2-BP
