# Supplementary figures and images for: A protein palmitoylation cascade regulates microtubule cytoskeleton integrity in Plasmodium
Source: EMBO J. 2020 May 12;39(13):e104168. doi: 10.15252/embj.2019104168 (PMC7327484; doi:10.15252/embj.2019104168)

Figure EV2

EV2-A ISP1/3

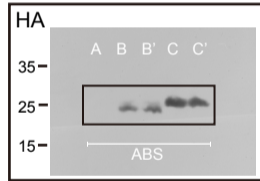

BiP

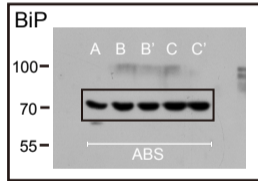

ISP1/3

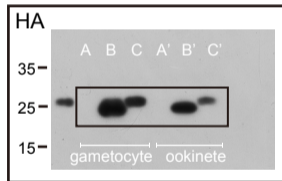

P28

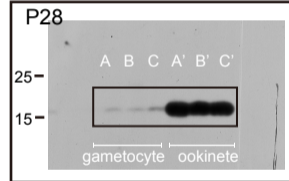

BiP

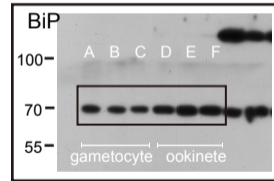

Lane A/A': wildtype  
Lane B/B': *isp1::6HA*  
Lane C/C': *isp3::6HA*

Supplement: Supplementary file 3 — Source Data for Expanded View [file EMBJ-39-e104168-s010.zip › embj2019104168-sup-0010-SDataFigEV/embj2019104168-sup-0010-SDataFigEV2.pdf]

# Figure EV5

EV5-E P28

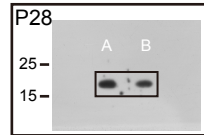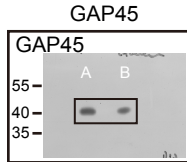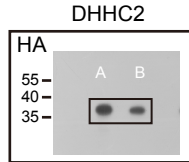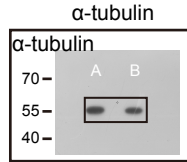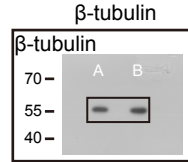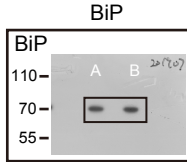

Lane A: TTS+DMSO  
Lane B: TTS+Colchicine

Supplement: Supplementary file 3 — Source Data for Expanded View [file EMBJ-39-e104168-s010.zip › embj2019104168-sup-0010-SDataFigEV/embj2019104168-sup-0012-SDataFigEV5.pdf]

Figure 1

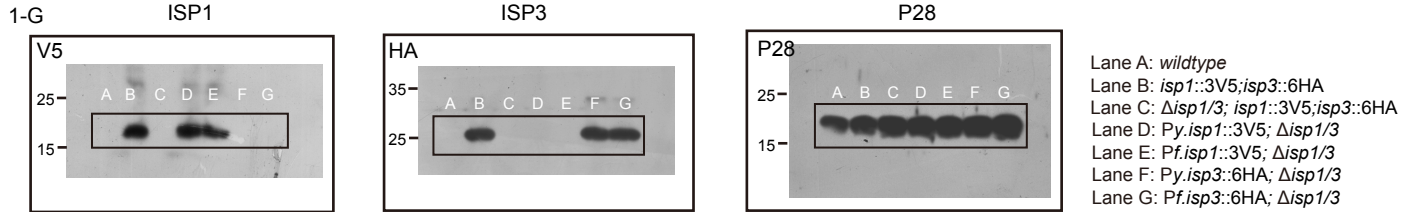

Supplement: Supplementary file 5 — Source Data for Figure 1 [file EMBJ-39-e104168-s003.pdf]

Figure 2

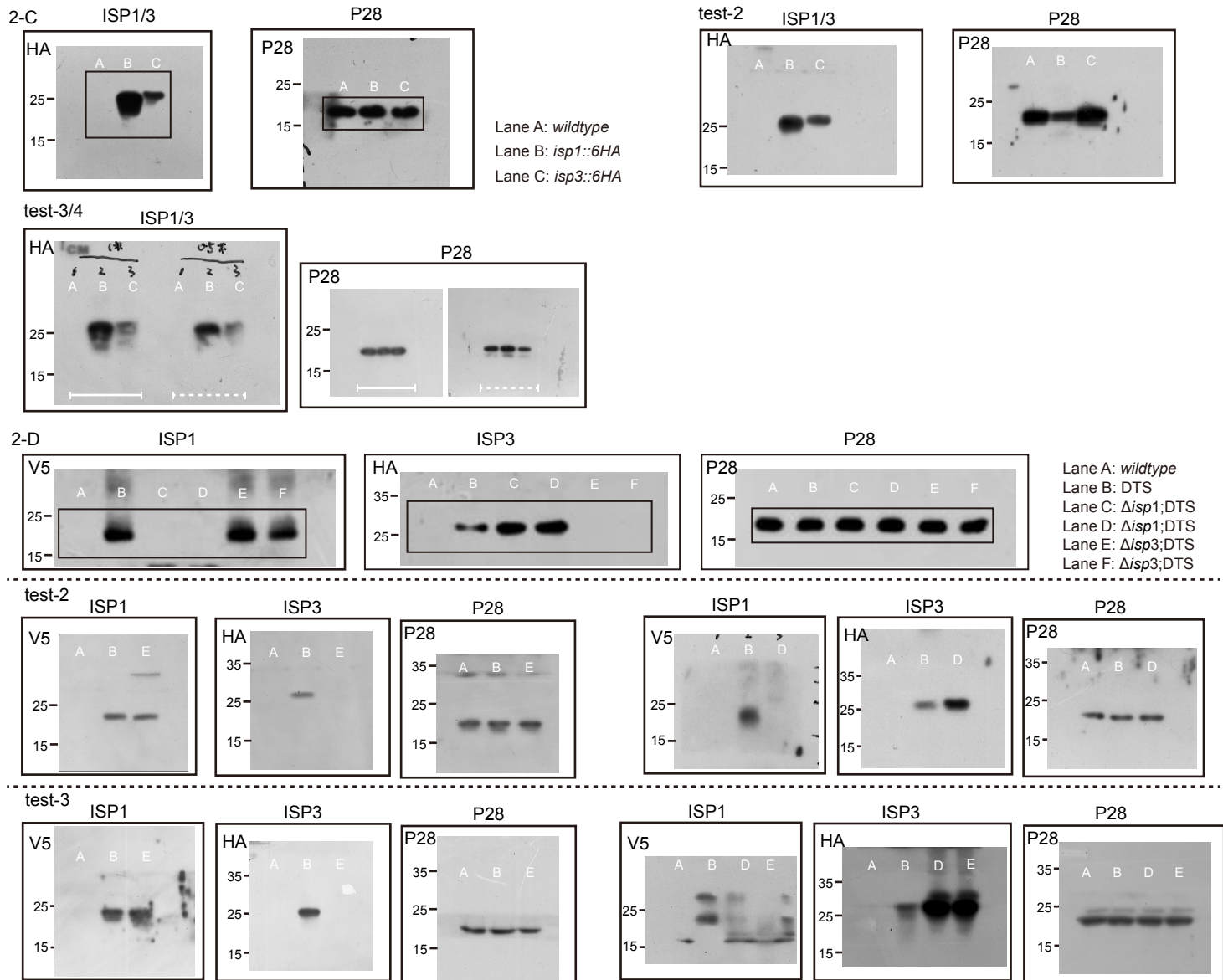

Supplement: Supplementary file 6 — Source Data for Figure 2 [file EMBJ-39-e104168-s004.pdf]

Figure 3

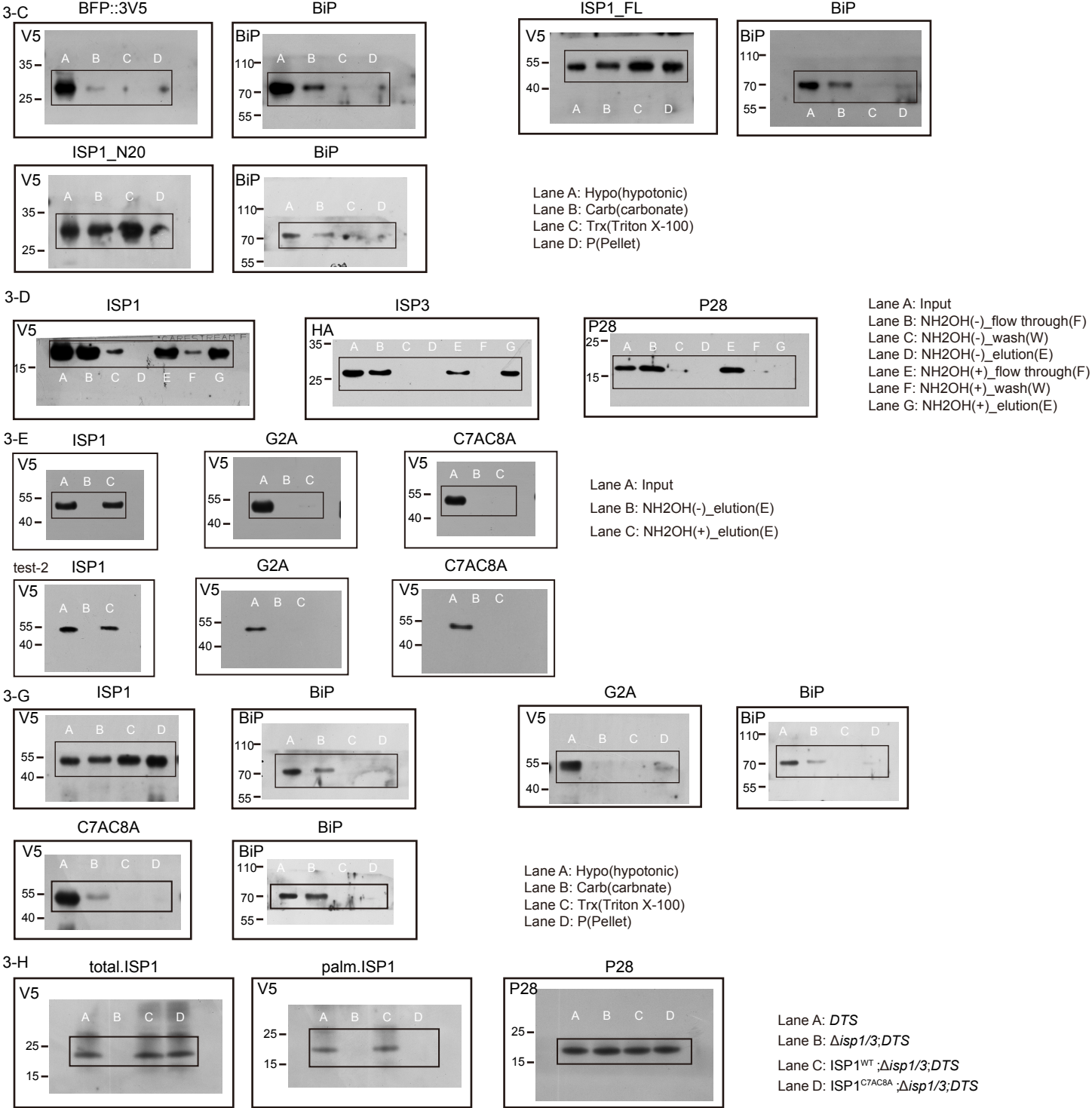

Supplement: Supplementary file 7 — Source Data for Figure 3 [file EMBJ-39-e104168-s005.pdf]

Figure 4

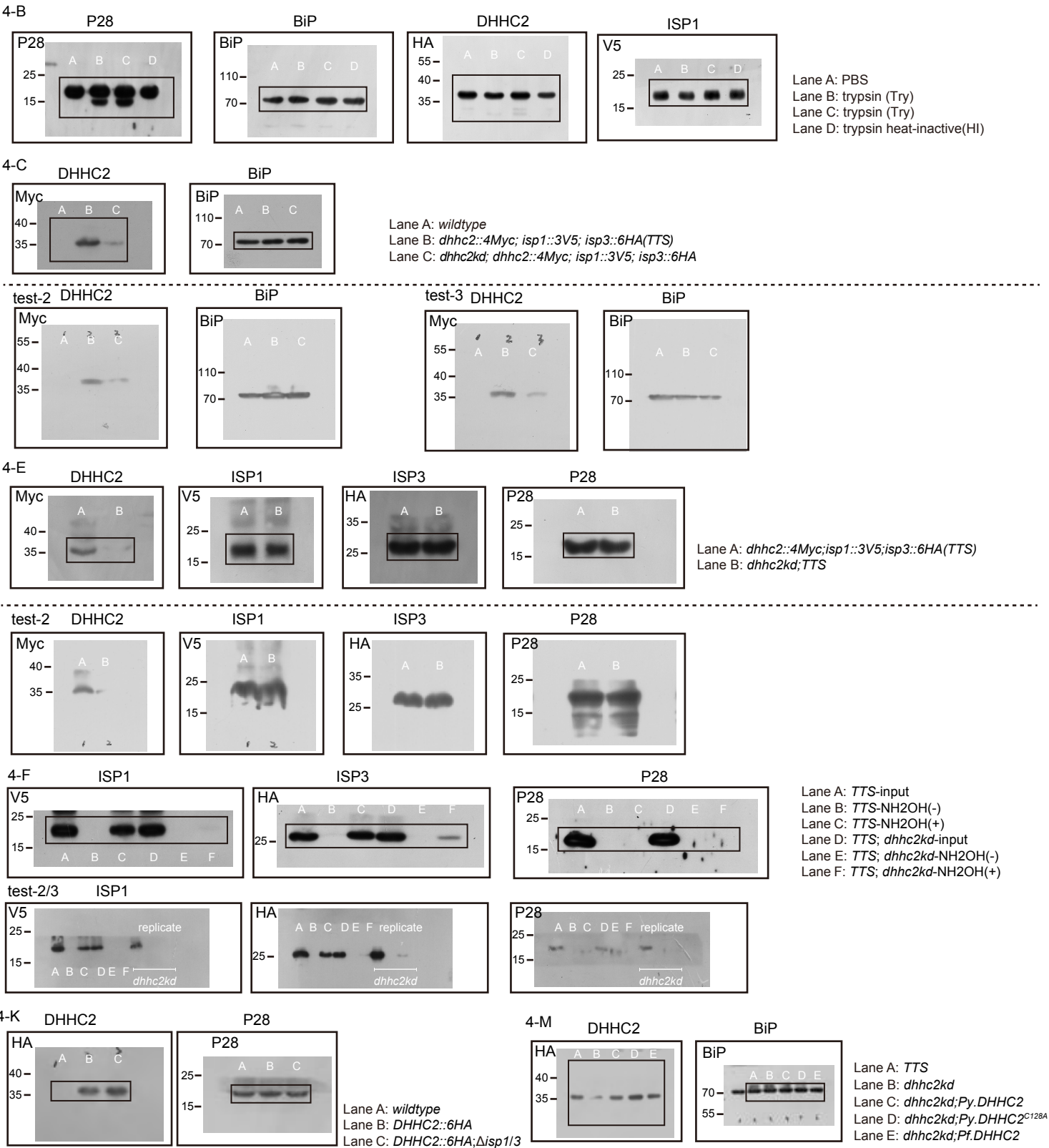

Supplement: Supplementary file 8 — Source Data for Figure 4 [file EMBJ-39-e104168-s006.pdf]

Figure 5

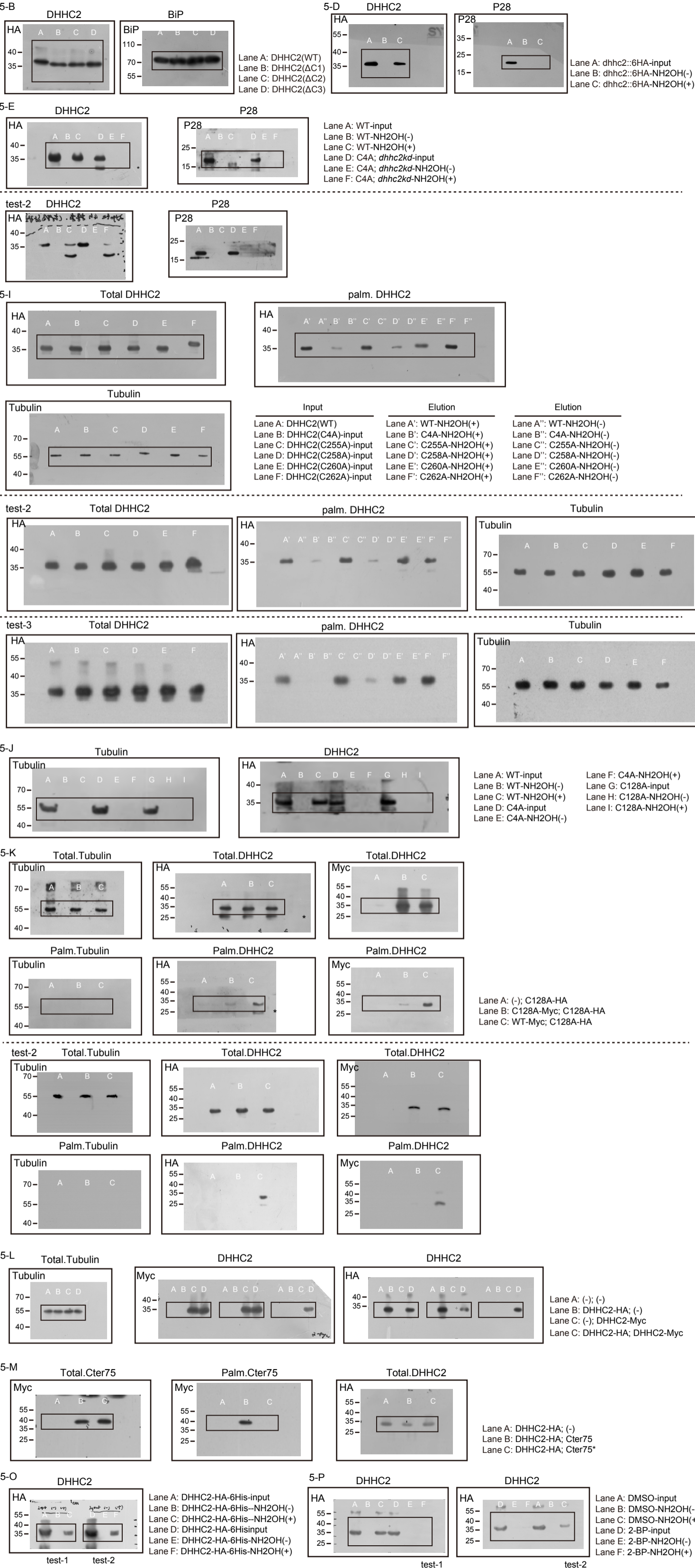

Supplement: Supplementary file 9 — Source Data for Figure 5 [file EMBJ-39-e104168-s007.pdf]

Figure 8

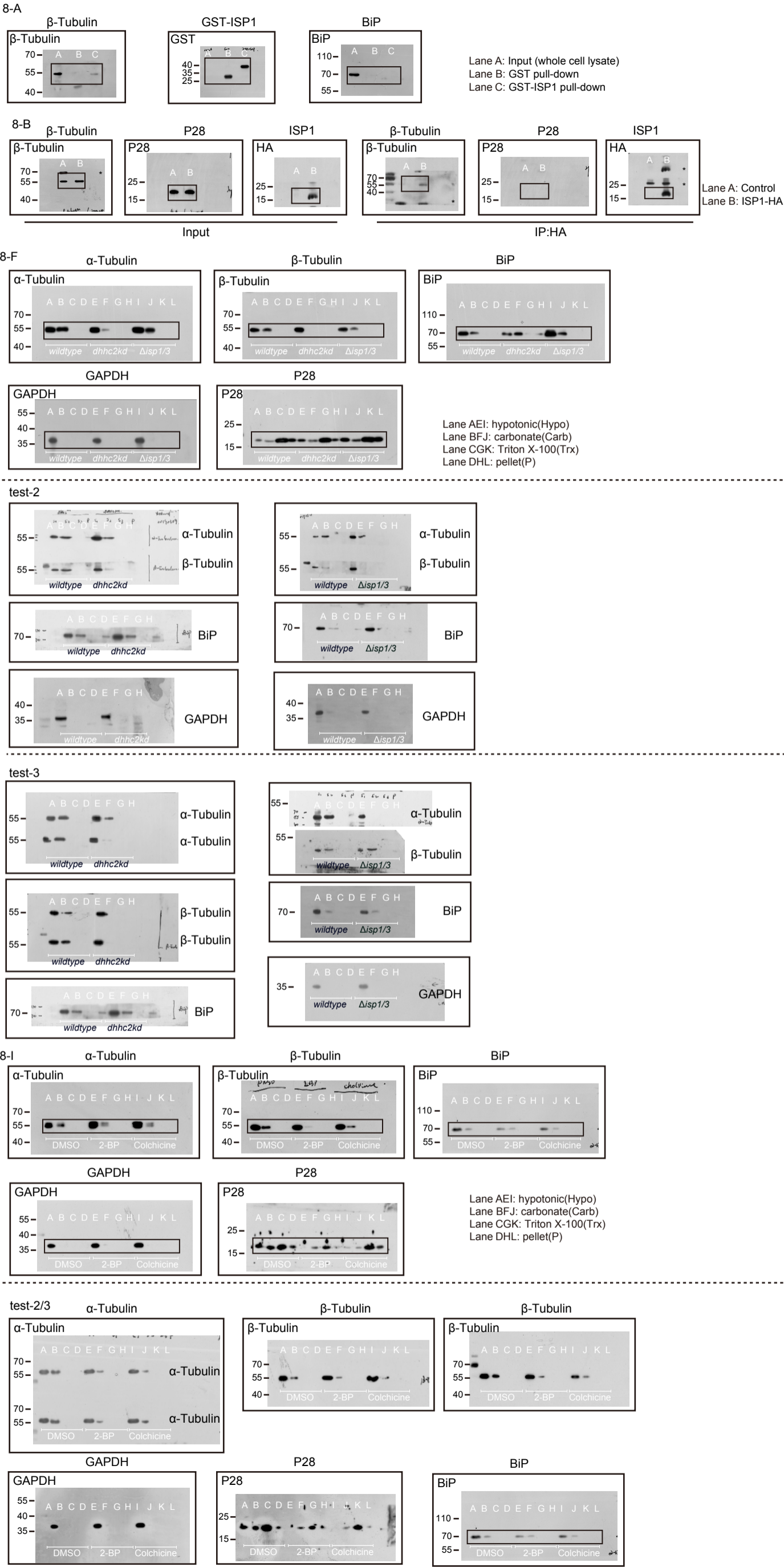

Supplement: Supplementary file 11 — Source Data for Figure 8 [file EMBJ-39-e104168-s009.pdf]
