# Supplementary material for: A protein palmitoylation cascade regulates microtubule cytoskeleton integrity in Plasmodium
Source: EMBO J. 2020 May 12;39(13):e104168. doi: 10.15252/embj.2019104168 (PMC7327484; doi:10.15252/embj.2019104168)
Supplement: Supplementary file 10 — Source Data for Figure 6 [file EMBJ-39-e104168-s008.pdf]

Figure 6

6-B

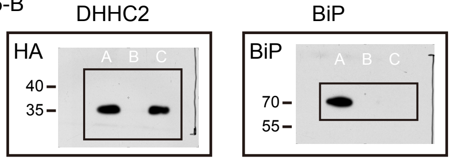

Lane A: *dhhc2::6HA*-input
Lane B: *dhhc2::6HA*-NH2OH(-)
Lane C: *dhhc2::6HA*-NH2OH(+)

6-C

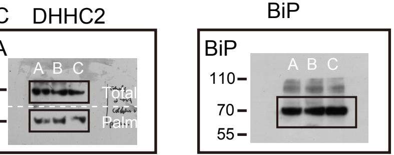

Lane A: Gametocyte
Lane B: Gamete
Lane C: Zygote

6-G

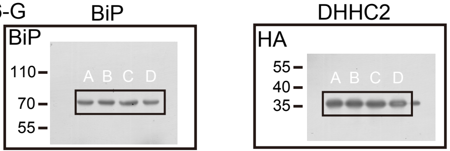

Lane A: *dhhc2::6HA*
Lane B: Δ*cdpk4;dhhc2::6HA*
Lane C: Δ*hap2;dhhc2::6HA*
Lane D: Δ*dozi;dhhc2::6HA*
