# Supplementary material for: A protein palmitoylation cascade regulates microtubule cytoskeleton integrity in Plasmodium
Source: EMBO J. 2020 May 12;39(13):e104168. doi: 10.15252/embj.2019104168 (PMC7327484; doi:10.15252/embj.2019104168)
Supplement: Supplementary file 4 — Review Process File [file EMBJ-39-e104168-s011.pdf]

THE
EMBO
JOURNAL

A Protein Palmitoylation Cascade Regulates Microtubule Cytoskeleton Integrity in *Plasmodium*

Xu Wang, Pengge Qian, Huiting Cui, Luming Yao, Jing Yuan

Review timeline:

Submission date:	2nd Dec 2019
Editorial Decision:	15th Jan 2020
Revision received:	15th Mar 2020
Editorial Decision:	2nd Apr 2020
Revision received:	3rd Apr 2020
Accepted:	16th Apr 2020

Editor: Ieva Gailite

Transaction Report:

(Note: No Peer Review Process File is available with this article, as the authors have chosen not to make the review process public in this case.)

EMBO PRESS

YOU MUST COMPLETE ALL CELLS WITH A PINK BACKGROUND

PLEASE NOTE THAT THIS CHECKLIST WILL BE PUBLISHED ALONGSIDE YOUR PAPER

Corresponding Author Name: Jing Yuan
Journal Submitted to: EMBO Journal
Manuscript Number: EMBOJ-2019-104168

Reporting Checklist For Life Sciences Articles (Rev. June 2017)

This checklist is used to ensure good reporting standards and to improve the reproducibility of published results. These guidelines are consistent with the Principles and Guidelines for Reporting Preclinical Research issued by the NIH in 2014. Please follow the journal's authorship guidelines in preparing your manuscript.

A- Figures
1. Data
The data shown in figures should satisfy the following conditions:

- the data were obtained and processed according to the field's best practice and are presented to reflect the results of the experiments in an accurate and unbiased manner.
- figure panels include only data points, measurements or observations that can be compared to each other in a scientifically meaningful way.
- graphs include clearly labeled error bars for independent experiments and sample sizes. Unless justified, error bars should not be shown for technical replicates.
- if n< 5, the individual data points from each experiment should be plotted and any statistical test employed should be justified
- Source Data should be included to report the data underlying graphs. Please follow the guidelines set out in the author ship guidelines on Data Presentation.

2. Captions

Each figure caption should contain the following information, for each panel where they are relevant:

- a specification of the experimental system investigated (eg cell line, species name).
- the assay(s) and method(s) used to carry out the reported observations and measurements
- an explicit mention of the biological and chemical entity(ies) that are being measured.
- an explicit mention of the biological and chemical entity(ies) that are altered/varied/perturbed in a controlled manner.

- the exact sample size (n) for each experimental group/condition, given as a number, not a range;
- a description of the sample collection allowing the reader to understand whether the samples represent technical or biological replicates (including how many animals, litters, cultures, etc.).
- a statement of how many times the experiment shown was independently replicated in the laboratory.
- definitions of statistical methods and measures:
  - common tests, such as t-test (please specify whether paired vs. unpaired), simple χ2 tests, Wilcoxon and Mann-Whitney tests, can be unambiguously identified by name only, but more complex techniques should be described in the methods section;
  - are tests one-sided or two-sided?
  - are there adjustments for multiple comparisons?
  - exact statistical test results, e.g., P values = x but not P values < x;
  - definition of 'center values' as median or average;
  - definition of error bars as s.d. or s.e.m.

Any descriptions too long for the figure legend should be included in the methods section and/or with the source data.

In the pink boxes below, please ensure that the answers to the following questions are reported in the manuscript itself. Every question should be answered. If the question is not relevant to your research, please write NA (non applicable). We encourage you to include a specific subsection in the methods section for statistics, reagents, animal models and human subjects.

USEFUL LINKS FOR COMPLETING THIS FORM

http://www.antibodypedia.com
http://1degreebio.org
http://www.equator-network.org/reporting-guidelines/improving-bioscience-research-repor
http://grants.nih.gov/grants/olaw/olaw.htm
http://www.mrc.ac.uk/Ourresearch/Ethicsresearchguidance/Useofanimals/index.htm
http://ClinicalTrials.gov
http://www.consort-statement.org
http://www.consort-statement.org/checklists/view/32-consort/66-title
http://www.equator-network.org/reporting-guidelines/reporting-recommendations-for-tum
http://datadryad.org
http://figshare.com
http://www.ncbi.nlm.nih.gov/gap
http://www.ebi.ac.uk/ega
http://biomodels.net/
http://biomodels.net/miriam/
http://jjj.biochem.sun.ac.za
http://oba.od.nih.gov/biosecurity/biosecurity_documents.html
http://www.selectagents.gov/

B- Statistics and general methods

Please fill out these boxes (Do not worry if you cannot see all your text once you press return)

1.a. How was the sample size chosen to ensure adequate power to detect a pre-specified effect size?	We used different sample sizes for different experiments. For analysis of zygote to ookinete differentiation, we are usually investigating more than 300 cells in each condition for each strain, while for determination of microtubule number by electron microscopy we aimed at 20 parasites. To determine parasite numbers in mosquitoes we are usually investigating morue than 40 mosquitos. Depending on the phenotype of the mutant not all these numbers could be matched, while usually they were exceeded. As we used inbred mice in this study to investigate parasite proliferation in mouse and mouse to mosquito transmission, we aimed to investigate at least 3 animals in each condition for each strain.
1.b. For animal studies, include a statement about sample size estimate even if no statistical methods were used.	We used animals (mice) here for parasite proliferation in mouse and parasite infection by mosquito bites. For the use of inbred mice the number is generally 3 mice each group in a single experiment.
2. Describe inclusion/exclusion criteria if samples or animals were excluded from the analysis. Were the criteria pre-established?	In this study no sample or animals were excluded from the analysis. One criteria to do so would have been a notification from the animal facility about potential secondary infections detected during the course of the study or a problem during injection of the animal with parasites.
3. Were any steps taken to minimize the effects of subjective bias when allocating animals/samples to treatment (e.g. randomization procedure)? If yes, please describe.	In critical experiments the data were blinded and counted by a separate student. These included the determination of microtubule numbers in EM images and zygote to ookinete differentiation rate.
For animal studies, include a statement about randomization even if no randomization was used.	Gametocyte containing parasite samples were randomized for in vitro phenotype assay. Mosquitoes were housed in the same cage using different compartments allowing simultaneous blood feeding from the blood containing WT and Knockout strain to avoid feeding bias.
4.a. Were any steps taken to minimize the effects of subjective bias during group allocation or/and when assessing results (e.g. blinding of the investigator)? If yes please describe.	The investigators were not blinded for experiments comprising objective measurements such as western, qPCR,IFA, and in vitro assays. In these cases, the analysis should not be influenced by a potential investigators bias.
4.b. For animal studies, include a statement about blinding even if no blinding was done	The investigators were blinded for analysis of the oocyst number per mosquito. The mosquito infection experiments were performed by two different graduate students coming to the same conclusion.
5. For every figure, are statistical tests justified as appropriate?	Yes, statistics were performed uisng two-tailed unpaired Student's t test, Mann–Whitney test, and Kolmogorov-Smironv test, which is appropriate for these kind of experiments.

Do the data meet the assumptions of the tests (e.g., normal distribution)? Describe any methods used to assess it.	To test for normal distribution, we used the Pearson omnibus normality test. Most data are normal distributed.
Is there an estimate of variation within each group of data?	Yes, the data are shown as mean ± SD or mean ± SEM in the figures.
Is the variance similar between the groups that are being statistically compared?	Not in all groups. The numbers of parasites decrease in mosquitoes the variance becomes larger between individual mosquitoes.

C- Reagents

6. To show that antibodies were profiled for use in the system under study (assay and species), provide a citation, catalog number and/or clone number, supplementary information or reference to an antibody validation profile. e.g., Antibodypedia (see link list at top right), 1DegreeBio (see link list at top right).	The details of the antibodies including primary and secondary are provided in the Material and Method.
7. Identify the source of cell lines and report if they were recently authenticated (e.g., by STR profiling) and tested for mycoplasma contamination.	The parasite P. yoelii 17XNL strain were used. Human 293T cell line is purchased from the ATCC.

* for all hyperlinks, please see the table at the top right of the document

D- Animal Models

8. Report species, strain, gender, age of animals and genetic modification status where applicable. Please detail housing and husbandry conditions and the source of animals.	All this info is stated in the materials and methods. ICR mice (female, 5 to 6 weeks old) were housed in the Animal Care Center of Xiamen University and kept at room temperature under a 12 h light/dark cycle at a constant relative humidity of 45%. Mice are housed in cages of 5 initially and upon increasing weight get split into cages housing 3 mice. The Anopheles stephensi mosquito (strain Hor) was reared at 28 ℃, 80% relative humidity and at a 12 h light/dark cycle. Mosquitoes were fed on a 10% sucrose solution.
9. For experiments involving live vertebrates, include a statement of compliance with ethical regulations and identify the committee(s) approving the experiments.	Animal experiments were performed in accordance with the approved protocols (XMULAC20140004) by the Committee for Care and Use of Laboratory Animals of Xiamen University.
10. We recommend consulting the ARRIVE guidelines (see link list at top right) (PLoS Biol. 8(6), e1000412, 2010) to ensure that other relevant aspects of animal studies are adequately reported. See author guidelines, under 'Reporting Guidelines'. See also: NIH (see link list at top right) and MRC (see link list at top right) recommendations. Please confirm compliance.	We confirme compliance.

E- Human Subjects

11. Identify the committee(s) approving the study protocol.	NA
12. Include a statement confirming that informed consent was obtained from all subjects and that the experiments conformed to the principles set out in the WMA Declaration of Helsinki and the Department of Health and Human Services Belmont Report.	NA
13. For publication of patient photos, include a statement confirming that consent to publish was obtained.	NA
14. Report any restrictions on the availability (and/or on the use) of human data or samples.	NA
15. Report the clinical trial registration number (at ClinicalTrials.gov or equivalent), where applicable.	NA
16. For phase II and III randomized controlled trials, please refer to the CONSORT flow diagram (see link list at top right) and submit the CONSORT checklist (see link list at top right) with your submission. See author guidelines, under 'Reporting Guidelines'. Please confirm you have submitted this list.	NA
17. For tumor marker prognostic studies, we recommend that you follow the REMARK reporting guidelines (see link list at top right). See author guidelines, under 'Reporting Guidelines'. Please confirm you have followed these guidelines.	NA

F- Data Accessibility

18: Provide a "Data Availability" section at the end of the Materials & Methods, listing the accession codes for data generated in this study and deposited in a public database (e.g. RNA-Seq data: Gene Expression Omnibus GSE39462, Proteomics data: PRIDE PXD000208 etc.) Please refer to our author guidelines for 'Data Deposition'.<br><br>Data deposition in a public repository is mandatory for:<br>a. Protein, DNA and RNA sequences<br>b. Macromolecular structures<br>c. Crystallographic data for small molecules<br>d. Functional genomics data<br>e. Proteomics and molecular interactions	We did not generate any data that needs to be deposited
19. Deposition is strongly recommended for any datasets that are central and integral to the study; please consider the journal's data policy. If no structured public repository exists for a given data type, we encourage the provision of datasets in the manuscript as a Supplementary Document (see author guidelines under 'Expanded View' or in unstructured repositories such as Dryad (see link list at top right) or Figshare (see link list at top right).	We had all the data in Expanded View Figures, Appendix Figures, and a source data file containing the scans of the western blots.
20. Access to human clinical and genomic datasets should be provided with as few restrictions as possible while respecting ethical obligations to the patients and relevant medical and legal issues. If practically possible and compatible with the individual consent agreement used in the study, such data should be deposited in one of the major public access-controlled repositories such as dbGAP (see link list at top right) or EGA (see link list at top right).	NA
21. Computational models that are central and integral to a study should be shared without restrictions and provided in a machine-readable form. The relevant accession numbers or links should be provided. When possible, standardized format (SBML, CellML) should be used instead of scripts (e.g. MATLAB). Authors are strongly encouraged to follow the MIRIAM guidelines (see link list at top right) and deposit their model in a public database such as Biomodels (see link list at top right) or JWS Online (see link list at top right). If computer source code is provided with the paper, it should be deposited in a public repository or included in supplementary information.	NA

G- Dual use research of concern

22. Could your study fall under dual use research restrictions? Please check biosecurity documents (see link list at top right) and list of select agents and toxins (APHIS/CDC) (see link list at top right). According to our biosecurity guidelines, provide a statement only if it could.	no
